# Towards the genomic sequence code of DNA fragility for machine learning

**DOI:** 10.1093/nar/gkae914

**Published:** 2024-10-23

**Authors:** Patrick Pflughaupt, Adib A Abdullah, Kairi Masuda, Aleksandr B Sahakyan

**Affiliations:** MRC WIMM Centre for Computational Biology, MRC Weatherall Institute of Molecular Medicine, Radcliffe Department of Medicine, University of Oxford, Oxford, OX3 9DS, UK; MRC WIMM Centre for Computational Biology, MRC Weatherall Institute of Molecular Medicine, Radcliffe Department of Medicine, University of Oxford, Oxford, OX3 9DS, UK; MRC WIMM Centre for Computational Biology, MRC Weatherall Institute of Molecular Medicine, Radcliffe Department of Medicine, University of Oxford, Oxford, OX3 9DS, UK; MRC WIMM Centre for Computational Biology, MRC Weatherall Institute of Molecular Medicine, Radcliffe Department of Medicine, University of Oxford, Oxford, OX3 9DS, UK

## Abstract

Genomic DNA breakages and the subsequent insertion and deletion mutations are important contributors to genome instability and linked diseases. Unlike the research in point mutations, the relationship between DNA sequence context and the propensity for strand breaks remains elusive. Here, by analyzing the differences and commonalities across myriads of genomic breakage datasets, we extract the sequence-linked rules and patterns behind DNA fragility. We show the overall deconvolution of the sequence influence into short-, mid- and long-range effects, and the stressor-dependent differences in defining the range and compositional effects on DNA fragility. We summarize and release our feature compendium as a library that can be seamlessly incorporated into genomic machine learning procedures, where DNA fragility is of concern, and train a generalized DNA fragility model on cancer-associated breakages. Structural variants (SVs) tend to stabilize regions in which they emerge, with the effect most pronounced for pathogenic SVs. In contrast, the effects of chromothripsis are seen across regions less prone to breakages. We find that viral integration may bring genome fragility, particularly for cancer-associated viruses. Overall, this work offers novel insights into the genomic sequence basis of DNA fragility and presents a powerful machine learning resource to further enhance our understanding of genome (in)stability and evolution.

## Graphical Abstract

**Graphical Abstract:**
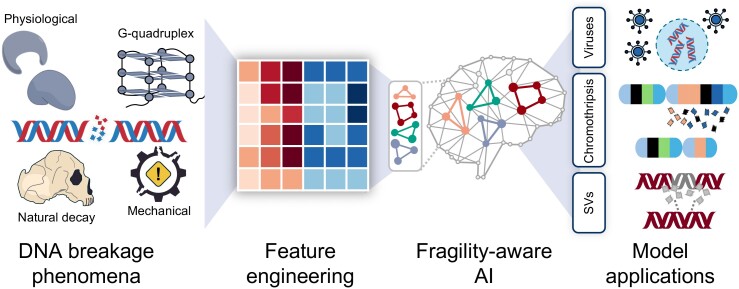

## Introduction

Genomic insertion and deletion alterations, which occur through the formation of DNA strand breaks, are the second most significant DNA modifications after point mutations (1,2). However, while the latter has been studied before (3,4), the short- and long-range sequence context patterns associated with DNA strand breakpoints have not been extensively interrogated through computational means. Nevertheless, several works have found associations of DNA strand breakpoints with non-B DNA conformations (5), cancer genes (6), mutations (7), abasic sites (8), chromatin packing (9), 3D genome organization (10) and the natural DNA decay processes (11). Early predictive models based on hidden Markov models and copy number variation data achieved coarse resolution (∼300 base pairs) for ∼400 breakpoints in the human genome (12). This demonstrates the potential for high-resolution, sequence-based prediction of DNA strand breaks. However, the advent of high-throughput sequencing technologies has enabled genome-wide mapping of DNA strand breakpoints at a nucleotide resolution (13–16), along with the availability of biological big data from both normal and pathological tissues (17–20). As such, advances in machine learning techniques now allow the development of models for studying genome-wide endogenous and disease-related DNA strand breaks reported experimentally (7,21–24).

This work aims to examine the general principles of the influence of genomic sequence on DNA fragility in different physiological and pathological conditions. By analyzing the patterns and commonalities across 100 genomic breakage datasets, we revealed that DNA sequence can be broadly categorized into having three, short-, medium- and long-range, effects within a 1 kb context window. In the short range, we quantified the DNA word (*k*-meric, where *k* is the length of the DNA segment) susceptibilities to various DNA breakage phenomena, as well as to various chromatin, epigenetic and structural alterations. These *k*-meric propensities are summarized into a compendium of features (DNAfrAIlib library), designed for easy integration into a sequence-driven machine learning model where DNA fragility accounting can be useful. Using these features and registered indel sites coming from unstable genomes in cancer, we developed a generalized machine learning model to predict DNA fragility for any sequence in human cell nuclei. Acknowledging that the precise mechanism of DNA breakage may not solely operate at a single-nucleotide level, our developed methodology assigns a breakage probability value to each specific position between adjacent nucleotides, based on its sequence context. Our model reveals distinct sequence-based characteristics that define regions of low, medium and high fragility across the entire human genome. Structural variants (SVs) tend to stabilize regions in which they emerge, and this effect is most pronounced for pathogenic SVs. In contrast, the effects of chromothripsis are seen across regions less prone to breakages. We find that viral integration may host genome fragility, particularly for cancer-associated viruses. Interestingly, the viruses of ectothermic species could be more fragile in a human host as compared to the viruses of other host species. This study offers a general overview of the underlying sequence basis of DNA fragility, provides a machine learning resource along with a range of insights to enhance our understanding in genome (in)stability and evolution, which we hope will be useful for further research.

## Materials and methods

### General notes on the performed calculations

The developed workflows and analyzes in this study employed the R programming language 4.3.2 (http://www.r-project.org/) and Python 3.9.12 (http://www.python.org). The resource-demanding computations were performed on a single NVIDIA RTX A6000 GPU with 40 GB RAM. Figures were created with the R base, ggplot2 3.4.4 (http://ggplot2.tidyverse.org), ggpattern 1.0.1 (http://cran.r-project.org/package=ggpattern), Matplotlib 3.5.1 (http://matplotlib.org) and Seaborn 0.11.2 (http://seaborn.pydata.org) libraries. Handling of the datasets was done by using the R base, tidyverse 2.0.0 (http://cran.r-project.org/package=tidyverse), data.table 1.14.8 (http://cran.r-project.org/package=data.table), purrr 1.0.2 (http://cran.r-project.org/package=purrr), Pandas 1.4.2 (http://pandas.pydata.org) and NumPy 1.26.1 (http://www.numpy.org) libraries. Processing of genomic sequences was done with the Biostrings 2.68.1 (http://bioconductor.org/packages/Biostrings) and plyranges 1.22 (http://www.bioconductor.org/packages/plyranges) libraries.

### General notes on the sourcing of data depicting various DNA breakage phenomena

The available public datasets are of substantial quantity and variability to make the investigation of various DNA breakage phenomena feasible. In any given study that deposited multiple biological replicates as separate datasets, those were merged into one, unless indicated otherwise.

### Sourcing of DNA breakage data

All genomic DNA sequence sources used in this study are of human origin, except the ancient DNA samples that we outline later. Twenty-five mechanically induced breakage datasets *via* ultrasonication frequencies (25) were retrieved from NCBI’s Sequence Read Archive (SRA) (http://www.ncbi.nlm.nih.gov/sra) Run Selector for the Bioproject PRJEB9586. Two nebulization-induced breakage datasets (26) were retrieved from data accession codes NA18794 and NA18795 from http://ftp.1000genomes.ebi.ac.uk/vol1/ftp/data. Five natural DNA decay and fossilization datasets from ancient DNA fragments were retrieved from the Max Planck Institute for Evolutionary Anthropology (http://ftp.eva.mpg.de/neandertal), representing one Denisovan (74–82k years old) (27), one Ust’-Ishim man (45k years old) (28) and three Neanderthal (50–80k years old) (29–31) genomes. We only retained the genomic ranges that pass certain criteria, including a coverage depth of at least ten, no tandem repeats or indels, no poor mappability or mapping quality below 25. These genomic ranges were retrieved as bed files from http://cdna.eva.mpg.de/neandertal/Chagyrskaya/FilterBed for the Chagyrskaya Neanderthal genome and from http://cdna.eva.mpg.de/neandertal/Vindija/FilterBed for the remaining genomes. Sixteen datasets of cell-free DNA (cfDNA) fragments coming from individual human peripheral blood plasma were obtained from NCBI’s SRA Run Selector for the Bioproject PRJNA291063 with the following SRA identifiers: SRX1120757 (healthy donor), SRX1120758 (Crohn’s disease), SRX1120760 (ulcerative colitis), SRX1120762 (systemic Lupus erythematosus), SRX1120768 (invasive/infiltrating ductal breast cancer), SRX1120766 (ovarian cancer), SRX1120767 (skin cancer melanoma), SRX1120769 (lung cancer adenocarcinoma), SRX1120771 (uterine cancer), SRX1120774 (colorectal cancer), SRX1120776 (prostate cancer), SRX1120777 (head and neck cancer), SRX1120779 (bladder cancer), SRX1120780 (liver cancer hepatocellular carcinoma), SRX1120781 (kidney cancer clear cell), SRX1120782 (testicular cancer seminomatous), SRX1120784 (pancreatic cancer ductal adenocarcinoma) and SRX1120793 (oesophagal cancer). Forty-six physiological breakage datasets were retrieved from various tissues and cell lines that arose through various endogenous and exogenous factors that disrupt normal cellular homeostasis (e.g., genomic instability associated with topology, replication, transcription, protein collisions, incomplete repair, etc). Such breakages are heterogeneous *via* the interplay of multiple cellular pathways and components. The datasets were retrieved from various tissues and cell lines: MDA-MB-231 cells (GSE115623), NHEK cells (GSE78172), single strand breaks (SSBs) in post-mitotic neuronal cells (GSE167257), native SSBs genome-wide in human leukemia K562 cells (GSE139011), human hTERT RPE-1 cells (GSE136943), human hematopoietic K562 cells (GSE121742), Caco-2 cells (GSE145594), from WRN helicase depletion experiments (32), from human recombination map (33) and human HL-60 leukemic cells (34). Five enzymatic cleavage datasets were retrieved, where restriction enzymes were added to the cell nucleus inducing single- or double-strand DNA cleavages at specific sequences *in vitro* under controlled conditions. These cleavages are more homogeneous with regards to the pattern of DNA breakages, hence requiring a separate label that distinguishes them from our collected physiological DNA breakage datasets. The retrieved datasets include restriction enzymes used in place of ultrasonication in the fragmentation process before sequencing (SRX7808529), or are specific restriction enzymes expressed inside a cell nucleus: Nt BbvCI (CCTCAGC sites) in H_2_O_2_-treated K562 cells (GSE139011), EcoRV (GATATC sites) in HeLa cells and AsiSI (GCGATCGC sites) in U2OS AID-DIva cell lines treated with 4-hydroxytamoxifen (4OHT) (GSE149709).

### Sourcing of epigenome data

In total, 39 datasets were retrieved from ChIP-seq, DNase-seq, FAIRE-seq, and ATAC-seq experiments, from public databases, including ENCODE and NCBI’s GEO, which were from cell lines for which we have DNA breakpoint data. From ENCODE (http://genome.ucsc.edu/encode), we retrieved MCF-7 (ATAC-seq, FAIRE-seq, DNase-seq), K562 (ATAC-seq, FAIRE-seq, DNase-seq), HCT116 (ATAC-seq, DNase-seq), HeLa-S3 (FAIRE-seq, DNase-seq), Caco-2 (DNase-seq), and HL-60 (DNase-seq) cell lines. We retrieved DNase-seq peaks representing DNaseI Hypersensitivity Clusters (V3) in 125 cell types from ENCODE. Six ChIP-seq datasets were retrieved from glioblastoma tumour-initiating cells for H3K4me1 (GSM5501175 and GSM5501176), H3K4me2 (GSM5501177 and GSM5501178) and H3K4me3 (GSM5501179 and GSM5501180). Four ChIP-seq SUMO (GSM1035424, GSM1035433, GSM1035426, and GSM1035435), two Ubc9 (GSM1035427 and GSM1035436) and two PIASy (GSM1035441 and GSM1035442) datasets were retrieved. Nine histone marks were obtained from GSE29611 and two from GSM945175. We retrieved a genome-wide profile of G-quadruplex sites from (35) as deposited on NCBI’s GEO under the accession identifier GSE63874. Further details are on our GitHub repository.

### Sourcing of transcription factor binding site data

We retrieved 247 core-validated vertebrate transcript factor binding sites (TFBS) from the JASPAR 2024 database (http://jaspar.elixir.no) in the bed file format. We lifted the genomic coordinates to the latest telomere-to-telomere (T2T) genome version using the UCSC CHM13v2 chain files (36) and the liftOver() (http://bioconductor.org/packages/liftOver) function from rtracklayer library 1.62.0 (http://bioconductor.org/packages/rtracklayer).

### Sequencing read alignment and identification of breakpoints

Genomic coordinates of the human reference genomes were retrieved *via* Bioconductor using the R programming language as documented in the associated literature from which we retrieved the DNA breakpoint datasets. The human reference genome hg37d5 was retrieved from http://bioconductor.org/packages/BSgenome.Hsapiens.1000genomes.hs37d5, and hg19 from http://bioconductor.org/packages/BSgenome.Hsapiens.UCSC.hg19.

The datasets obtained for the investigation of DNA breakage phenomena (from ultrasonication, nebulization, natural decay and fossilization, Twist library-enzymatic protocol, and cfDNA from the blood plasma) were already pre-processed, aligned, and deposited on NCBI’s SRA repository as FASTA files. However, we wanted to add an additional layer of quality checks when extracting their DNA breakpoint locations. We have thus first aligned each sequencing read to both the plus and minus strands of the human reference genome corresponding to the version used in the study from which we retrieved the DNA breakpoint datasets. This was done using the edlib library 1.2.7 (37) in C++ and Rcpp library 1.0.11 (http://cran.r-project.org/package=Rcpp), with parameters mode = HW for the infix alignment method, to select the better alignment based on the Levenshtein distance metric, prioritizing those results, where the first two bases matched the reference genome. As our analyzis relies on the DNA breakage between adjacent nucleotides, and we have found several cases where the Levenshtein distance was low (a low value indicates that two sequences are highly similar; a value of zero indicates that they are identical for the particular region compared), yet the first two bases did not result in an exact match to the corresponding region, we felt it was necessary to omit such sequencing reads to eliminate possible false positive DNA breakpoints. This alignment process was done for each autosome separately. The final alignment check was performed by comparing across autosomes, i.e. if the Levenshtein distance for a particular alignment is below the μ + σ deviation of the overall distance across all autosomes, we retained these breakpoint positions. The above gave us confidence that, despite having access to already pre-processed and aligned FASTA sequences, the additional quality checks omitted any potential leftover false positive DNA breakpoint positions.

Next, we subjected all the remaining DNA breakpoint locations to one additional level of filtering by removing any positions that occurred within the defined ENCODE Blacklisted Regions (38) to ensure any artificially high signals would not contaminate our downstream analyzes of the DNA breakpoints. However, we note that the overall genomic sequence context effects on DNA fragility are hardly affected when removing these ENCODE Blacklisted Regions, except for the data coming from ancient DNA samples. The remaining DNA breakage datasets (accessed predominantly as BED files) had already undergone rigorous post-processing pipelines as fully documented in their associated studies from which those were retrieved. The presence of an RMSD peak at the central breakpoint location (Figure 2C; Supplementary Figures S3–S7 and S12) gave us confidence that the analyzed DNA breakpoint locations are correctly aligned and processed, hence not giving us any major concerns for subsequent downstream analyzes.

**Figure 1.**
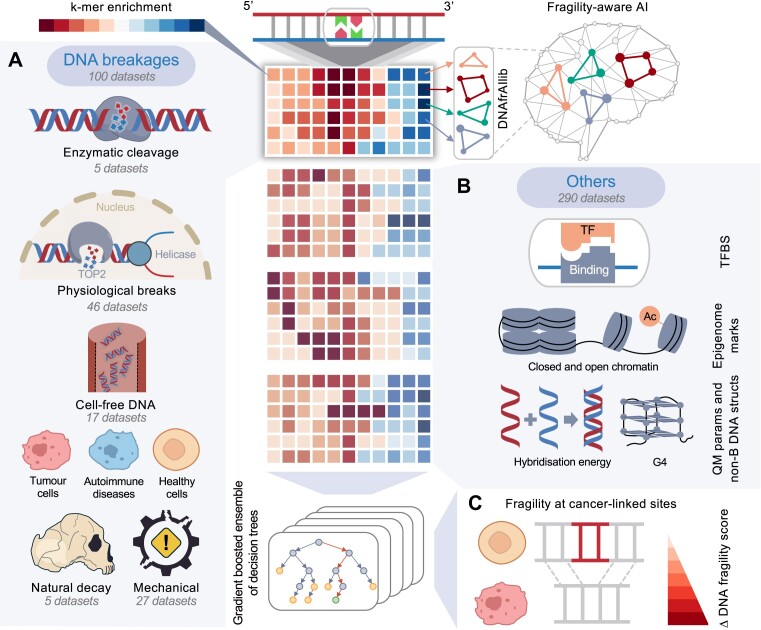
Design and workflow of the study. (**A**) Hundred available public datasets of DNA breakages were retrieved from a wide range of sources. Five samples came from restriction enzymes inducing single- or double-strand DNA cleavages. Forty-six samples came from endogenous double strand breaks (DSBs) through various biological stressors. Forty-nine samples came from high-frequency DSBs, of which 17 are through short fragments of cfDNA floating in the blood plasma, 27 are mechanically induced breakages (ultrasonication and nebulization) and five are caused by the natural decay processes leading to short ancient DNA fragments derived from the following genomes: Denisovan (74–82k years old) (27), Neanderthal (50–80k years old) (29–31) and Ust’-Ishim man (45k years old) (28). We converted the sequence context effects for each sample into parameterized *k*-meric susceptibility scores (DNAfrAIlib library), indicating the enrichment and depletion of a given *k*-mer when exposed to the breakage phenomenon under study. (**B**) We applied the same process as in (A) to extract and quantify *k*-meric enrichment and depletion of various other genomic features linked to genome stability and fragility. This includes the core, validated human transcription factor binding sites (TFBS) from the JASPAR database (http://jaspar.genereg.net), general chromatin features using various ChIP-seq, ATAC-seq, DNase-seq and FAIRE-seq datasets, mainly from the ENCODE database (http://genome.ucsc.edu/encode). We also utilized various structural, energetic and quantum mechanical properties of DNA and its various conformations. By aggregating each processed sample, we enabled an artificial intelligence (AI) engine to capture the DNA fragility phenomena, underlying biology, chemistry, physics and mechanical aspects. (**C**) One of the applications of the AI engine is to study DNA fragility at cancer-linked sites and predict vulnerable genomic loci prone to breakages.

**Figure 2.**
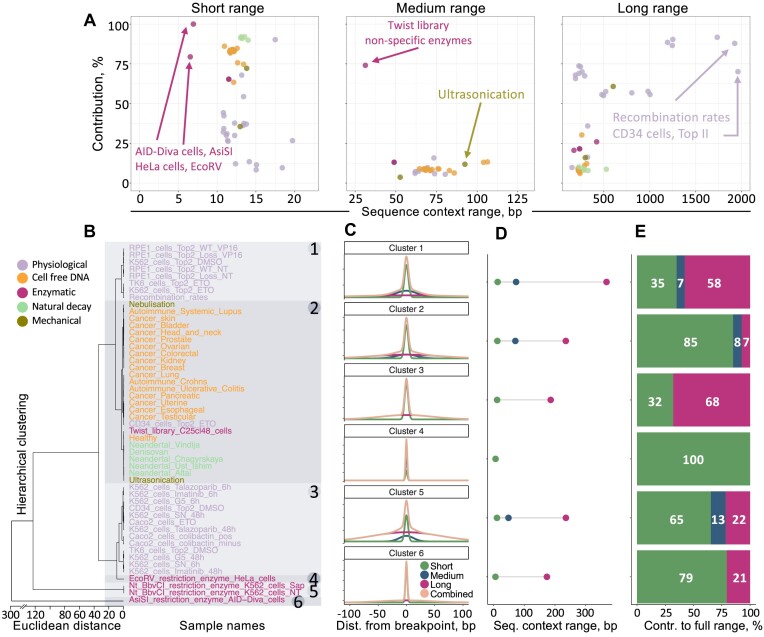
Sequence-based mechanistic insights into different classes of DNA breakages reveal multiple ranges of sequence influence towards the breakpoint formation. (**A**) Each sample represents a type of DNA breakage induced under a specific condition, with the overall sequence context exerting varying levels of influence on breakpoint formation. The influence can come from immediate neighbouring effects representing the short-range influence, medium-range effects and long-range sequence effects. We quantified the influence of each range on breakpoint formation relative to the combined effects of all ranges for each sample. A select few outliers by range (*x*-axis) and contribution (*y*-axis) in each sequence range effect are annotated. We limited the annotation of more outliers to avoid crowding the three plots. Full annotation is in Supplementary Figure S3. The μ ± σ of each sequence range effect is as follows: 13 ± 2 bps (short range), 71 ± 15 bps (medium range) and 473 ± 456 bps (long range). The long-range sequence effects display the highest variation around the 473 base window average range effect. (**B**) We identified six distinct clusters based on the similarities of the full-range sequence context effect. The clusters were determined through hierarchical clustering. Clusters 1 and 3 involve various physiological breakages. Cluster 2 includes all high-fragmentation breakages, including mechanical and cell-free cfDNA of human origin, and the natural decay and fossilization process in forming ancient DNA fragments derived from Denisovan (74–82k years old), Neanderthal (50–80k years old) and Ust’-Ishim man (45k years old), some biologically induced DSBs involving the stabilization of the cleavage complex between a broken DNA segment and Topoisomerase II, and non-specific enzymatic cleavages with the Twist enzyme library. Clusters 4–6 are experiments in which a specific restriction enzyme is expressed inside a cell. (**C**) For each cluster, we averaged the sequence context effects and fitted two or three normal distributions to the average effects, revealing the deconvolution into short-, medium- and long-range sequence-driven effects. The ranges of influences are as follows. Cluster 1: 7 bps (short) and 174 bps (long); cluster 2: 12 bps (short), 49 bps (medium) and 236 bps (long); cluster 3: 12 bps (short) and 186 bps (long); cluster 4: 7 bps (short); cluster 5: 13 bps (short), 71 bps (medium) and 235 bps (long); cluster 6: 14 bps (short), 74 bps (medium) and 368 bps (long). Panel (**D**) shows the sequence context for each range effect and (**E**) shows how much each range contributes to the full-range sequence context effect. Long-range effects are observed in all clusters, except for cluster 4, while medium-range effects are seen across clusters 1, 2 and 5. The colour coding for each sample indicates the general class of breakage it represents, which is used throughout the study.

### Quantifying the ranges for intrinsic sequence influence

We quantified the range of sequence influence by aligning strand-invariant breakpoint positions to the 5’ end of the breakage site on the plus strand of the human genome. For *k*-mers *k* ∈ {2, 4, 6, 8} and their reverse complements, we calculated their frequencies at the breakpoint and within a 1 kb range around it. These frequencies were normalized to the total counts. Adjacent positions were compared using the root-mean-squared deviation (RMSD) across each autosome, then averaged per position. This was done in C++, kseq.h (http://github.com/attractivechaos/klib) for sequence parsing and phmap.hpp (http://github.com/greg7mdp/gtl) for data storage. The incorporation of these two files, commonly used in computational biology, is documented in detail in the README of our GitHub repository (http://github.com/SahakyanLab/DNAFragility_dev), outlining the source and the way to download and install those (with all the links included).

Most RMSD plots could be explained by fitting up to three Gaussian distributions (Supplementary Figure S1) using the nls() function from the base R stats package (http://www.r-project.org) with parameters set to nls.control(maxiter=50000, tol=1e-05) and algorithm=port. The port algorithm was used to allow us to set lower and upper bounds for the Gaussian curves to be fitted within the calculated 1 kb window, while setting the mean of the curve to the breakpoint origin at the zero position. As the convergence is sensitive to the starting conditions, we used a grid search-based approach to optimize the starting values of the coefficients. By default, the optimization was first performed for fitting three Gaussian curves, each potentially revealing independent types of short-, medium- and long-range sequence-driven influences (Supplementary Figure S1 for the diminishing returns of fitting more than three Gaussian curves). When multiple normal distributions were fitted to the underlying distribution, we took the linear combination of those Gaussian curves. Another grid search was performed for fitting two Gaussian curves if any of the following conditions were met: (i) the algorithm failed to converge, (ii) any Gaussian curve contributed <5% towards the linear combination of all fitted curves by the measure of the Gaussian peak as a fraction of the Gaussian peak of all linearly combined curves, (iii) any Gaussian peak coefficient was zero or (iv) any Gaussian standard deviation was zero. An alternative approach to condition (ii) would be to calculate the ratio of the area under the curves (AUC). However, this measure may skew the outcome. As the short-range effect is often a sharp peak with a narrow standard deviation, its AUC would inevitably end up being much smaller than a long-range effect with a shallow peak but spanning a wide region. Given the biological significance of the immediate bases surrounding the location of a breakpoint (see ‘Results and Discussion’ section), the ratio of the Gaussian peaks made more sense as a measure of the contribution towards the signal. To define the different ranges in a statistical and unbiased way, we used the 95% confidence interval. This method excluded datasets from peak calling software.

To demonstrate that the characteristic peak seen across all DNA breakage conditions is indeed a true positive signal of a sequence-based influence, we conducted a negative control study, in which we randomly sampled 30 million breakpoints in each autosome and quantified the sequence effects within a 1 kb window of the central negative control strand break. This experiment was independently repeated ten times with different random seeds. The results show that a negative control breakpoint is not influenced by its sequence context, illustrated by the random fluctuation of the RMSD value and lack of the characteristic peak we observed in true positive DNA breakpoints (Supplementary Figure S2). This fluctuation is not only small in magnitude but also reflects the background noise observed when moving upstream and downstream, away from the central true positive strand break (Figure 2 and Supplementary Figures S3–S7). Hence, when our calculation reveals a characteristic peak at the central position with an asymptotic decay on either side, we can be confident that this likely reveals an intrinsic sequence-based fragility profile.

### Clustering sequence effects

All clustering in this work was done using the standard, agglomerative-based hierarchical clustering on the Euclidean distances between all extracted sequence ranges with the ward linkage method argument. The silhouette method and the total within the sum of squares were both used as guidance. The branches in the resulting dendrogram are distinctly separated, as per the evaluation criteria and their sharp correspondence with the categories of the underlying fragmentation phenomena, making six clusters a reasonable convergence. Instead of pre-labelling the fitted Gaussian curves, we took all the ranges, flattened and log_2_-transformed them, and cut the tree into the notably distinct three groups to represent the short-, medium- and long-range effects (Supplementary Figure S2).

### Quantifying DNA flexibility at EcoRV and Nt BbvCI cleavage sites

We focused on the cleavage sites of Nt BbvCI and EcoRV enzymes in K562 and HeLa cell lines, respectively. Using Hi-C subcompartment data from Xiong and Ma (39), we analyzed the breakage data within A (open) and B (closed) chromatin regions. To examine the DNA shape parameters within the medium to long-range effects, we employed the DNAshapeR library 1.30 (http://bioconductor.org/packages/release/bioc/html/DNAshapeR.html) (40) to calculate four DNA shape parameters: the minor groove width, propeller twist, roll and helical twist in a five-nucleotide sliding window in strides of one nucleotide. We examined the structure-driven effects at G-quadruplex (G4) sites following the methodology from (41) using G4-seq confirmed sites from (35) as deposited on NCBI’s GEO under the accession identifier GSE63874.

### Quantifying DNA fragility *viak*-meric enrichment

To quantify the short-range *k*-meric fragility, we compared the population of broken *k*-mers with a negative control population sampled away from breakage sites to avoid any sequence effects. The control regions varied in size based on the dataset: where possible, we used the long-range sequence influence span plus 1k bases; otherwise, we averaged the top 5% longest breakage regions to set control region boundaries (e.g. epigenome data). We used our in-house kmeRtone programme (http://github.com/SahakyanLab/kmeRtone) to calculate *z*-scores between *k*-mers, as:


(1) \begin{eqnarray*} z_{i} = \frac{X_{i}-np_{i}}{\sqrt{np_{i}(1-p_{i})}} \end{eqnarray*}

where *n* is the total count of a broken *k*-mer, *p* is the proportion control, and *np* is the predicted case distribution for all counts of a *k*-mer in a vector, **X**. Thus, the *z*-score values can determine the propensity of breakage at a given *k*-mer, where a higher *z*-score (*z* > 1) implies high susceptibility towards the breakage phenomenon while a lower *z*-score (*z* < −1) implies intrinsically low susceptibility. The conditional probability of a breakage given a *k*-mer was calculated using Bayes’ theorem:


(2) \begin{eqnarray*} P(break|kmer) = \frac{P(break)*P(kmer|break)}{P(kmer)} \end{eqnarray*}

where the estimate for *P*(*k*mer) is the normalized relative frequency of the control population for each *k*-mer, and the estimate for *P*(*k*mer|break) is the similar frequency, but in the breakage population, for each *k*-mer. Any overlapping regions of breakage sites were merged and any portion of the control region that overlapped with the breakage sites was removed. This allowed us to take a much longer range in the control region for the above-described alternative approach, where we average the top 5% longest breakage regions to set the control region boundaries. If a *k*-mer had insufficient sample size for a *z*-test to be calculated, we attributed a value based on the average *z*-score of the nearest five *k*-mers by sequence similarity.

### Clustering and analyzes of *k*-meric enrichment and depletion

We normalized the *k*-mer fragility scores and performed hierarchical clustering on them using the Ward linkage method. The results were visualized as a heatmap using the gplots library 3.1.3 (http://cran.r-project.org/package=gplots) with ten colour gradients from the RColorBrewer library 1.1-3 (http://cran.r-project.org/package=RColorBrewer) to represent the *k*-meric enrichment (red) and depletion (blue). Hierarchical clustering grouped breakage samples into six distinct clusters. Focusing on the top 1% most significant outliers among their *k*-meric differences (0.5% on either side), we compared the two groups of *k*-meric profiles, first by their sequence logos using the ggseqlogo library 0.1 (http://cran.r-project.org/package=ggseqlogo). However, as no significant motifs were identified through this method, we instead focused on the underlying and more abstracted properties of a sequence, comparing their hybridization energies (42) to obtain aggregate biophysical properties of these extreme groups of *k*-mers. We estimated the full hybridization energies based on the triplet hybridization energies by using a one-nucleotide sliding window and an averaging approach. Any two groups of outlier *k*-mers were compared using the two-sample *t*-test with the ggsignif library 0.6.4 (http://cran.r-project.org/package=ggsignif). We repeated the calculations with the change in heat of formation of octamers in the B-DNA conformation from (43).

### Correlating *k*-meric fragility profiles across DNA breakage and epigenome datasets

**Figure 3.**
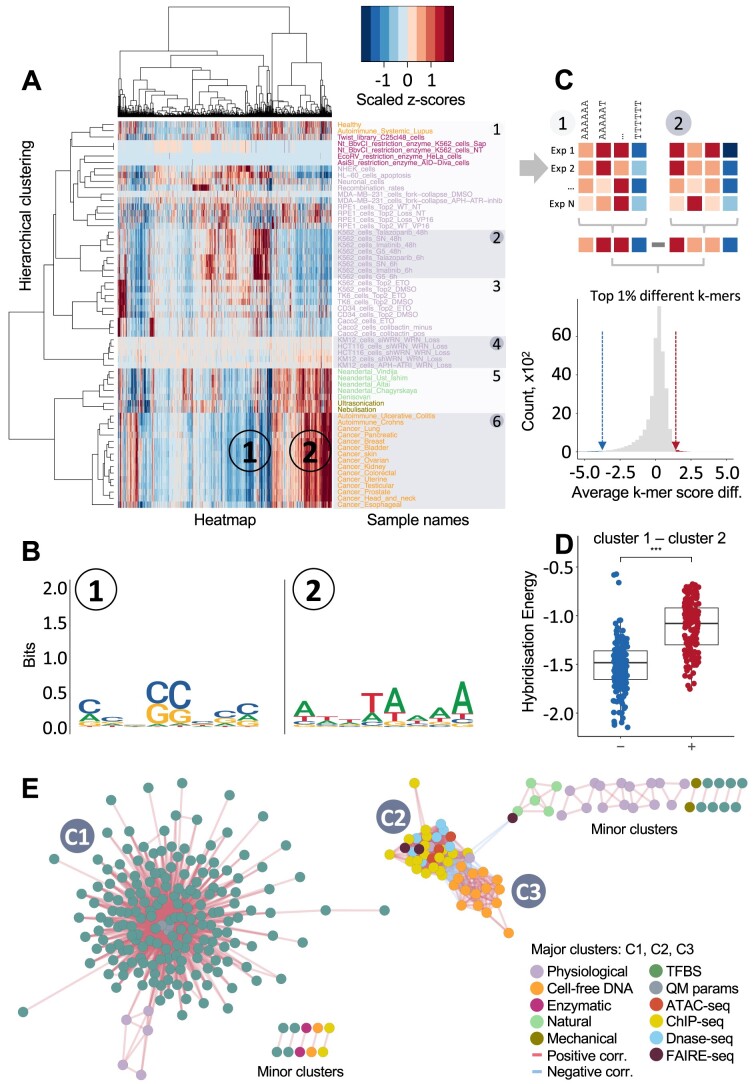
Compendium of short-range intrinsic DNA breakage propensities under various conditions. (**A**) depicts the normalized *k*-meric intrinsic susceptibility scores for each sample. The arrangements of 61 samples (rows) and 32,896 *k*-mers (columns) are determined through hierarchical clustering, as shown in Figure 2. Two regions are highlighted on the heatmap, which harbours a highly depleted cluster of *k*-mers (i) and a highly enriched cluster of *k*-mers (ii), with sequence logos brought in (**B**). The breakage samples (rows) were clustered into ten groups. (**C**) To compare the *k*-meric profiles across each cluster, we averaged the enrichment and depletion values for each *k*-mer, and position-wise subtracted the averaged values from one cluster to another. We then focused on the outliers: the top 0.5% *k*-mer differences on either side denoted by the vertical lines. (**D**) Focusing on the outliers, we compared the hybridization energies in the B-DNA conformation between the two groups of extreme *k*-mer populations to reveal any aggregate characteristics of the DNA structure at the breakpoint site, visualized as a boxplot. The left boxplot (−) represents highly resistant *k*-mers in cluster 1 compared to cluster 6, while the right boxplot (+) represents highly susceptible *k*-mers in cluster 1 compared to cluster 6, brought as an example. Centre lines represent median values; box limits represent the interquartile range; whiskers extend each 1.5 times the interquartile range. A two-sample *t*-test was performed between the *k*-mer populations, revealing a significant difference of *P* = 5.912 × 10^−37^. (**E**) To investigate the relations among each sample, we computed the Pearson correlation coefficient across all DNA breakage samples (Figure 1A) and samples from other chromosomal features (Figure 1B), calculated the Euclidean distances and visualized it using a network graph, where any two nodes connected by an edge denote the correlation between the two samples. To focus on the most important connections, we eliminated the self-correlations by removing diagonal entries and discarding Pearson correlation coefficients that are below absolute 0.7. Notable major clusters are highlighted in C1, C2 and C3. The colour coding for each sample indicates the general class of breakage it represents, which is used throughout the study. While there are some minor clusters, each containing 2–4 elements of similar datasets, we avoided the explicit labelling of all of these minor clusters to prevent (E) from becoming overcrowded and illegible.

We calculated the Pearson correlation coefficient between all normalized *k*-meric fragility scores and used the same hierarchical clustering process as previously described to analyze relationships between datasets. To remove the self-correlation, we eliminated the diagonal entries and discarded the Pearson correlation coefficients that were below absolute 0.7. Correlations were visualized in a network graph using the Cytoscape *via* RCy3 library 2.22.1 (http://bioconductor.org/packages/RCy3). All nodes were shaped as ellipses, however, the labelling was done only for the DNA breakage samples, while the epigenome samples were unlabelled to avoid crowding the plot (Figure 3E). Positive correlations between nodes were visualized as red edges, while negative correlations were visualized as blue edges. The graph was arranged using a force-directed layout with the default spring coefficient set to 0.000003 and the default spring length set to 70. Due to the long names of a select few node labels, we manually separated them to allow better visual appearances, while keeping the overall network clustering intact.

### Employed machine learning techniques and model-tuning

We employed two machine learning models: a logistic classifier and Gradient Boosting Machines (its LightGBM flavour) using Python’s sklearn library 1.3.1 (http://scikit-learn.org) and lightgbm library 4.1.0 (http://github.com/microsoft/LightGBM), respectively.

### Feature extraction for machine learning

For our control breakpoint sites, we used the coordinates of these negative control regions. We deliberately sampled more negative control breakpoints from the control regions as this imbalance is generally seen in practice and to potentially improve the understanding of the region surrounding broken sites. This resulted in 8,424,462 data points with a ratio of 39% true and 61% control breakpoints, and 421 features. The full feature matrix was randomly partitioned (seed = 1234) into 70% and 30% for training and pure test sets, respectively, with the caret library 6.0-94 (http://cran.rproject.org/package=caret).

For any given position, we extract purely sequence-based features corresponding to the window of short-, medium- and long-range sequence context effects as quantified in previous sections. We also ensured that any potential overlap, and by extension, any sequence-based influences, between true and negative control breakpoints are fully avoided by removing overlaps that fall within the short- and medium-range spans. We deemed any overlap within the long-range span acceptable as the signal-to-noise ratio was minuscule beyond the medium-range span.

Within the short-range span, we expanded a breakpoint into an 8-mer sequence, for which we extracted the *k*-meric enrichment and depletion *z*-scores representing the various DNA breakage phenomena, transcription factor (TF) binding sites, the change in the heat of formation of DNA at its various conformations, G4-seq maps, and epigenome marks (Figure 1A and B). For quantifying the potential to form stem-loop structures, we used the viennaRNA library 2.6.4 (http://github.com/ViennaRNA/ViennaRNA) with DNA parameters (44). We identified putative quadruplex sequences (PQSs) *via* the sequence motif *G*_*g*1_*N*_*x*_*G*_*g*2_*N*_*y*_*G*_*g*3_*N*_*z*_*G*_*g*4_, where *g*1, *g*2, *g*3 and *g*4 are integers which can be three or higher; *x*, *y* and *z* are any integers within the range of one to the maximum loop size; and *N* stands for any nitrogenous base, including guanine. The search for PQSs themselves also intrinsically search for *i*-motifs due to the complementary nature of their general motifs. The DNAshapeR library 1.30 (http://bioconductor.org/packages/release/bioc/html/DNAshapeR.html) (40) was employed to calculate four DNA shape parameters: the minor groove width, propeller twist, roll and helical twist in a five-nucleotide sliding window in strides of one nucleotide. Aggregate statistics of the long-range features (G+C content and G+C skew, as defined by [G-C]/[G+C]) were calculated using the letterFrequency() function from the Biostrings library 2.68.1 (http://bioconductor.org/packages/Biostrings/). Our *k*-meric intrinsic susceptibility *z*-scores provided insight into the fragility or resistance of various *k*-mers of the DNA breaking under different conditions. These values were also used within the long-range span as a proxy of sequence context fragility. This allowed us to scale the *k*-mer counts with their corresponding *k*-meric intrinsic susceptibility *z*-scores for each DNA breakage phenomenon.

### Breakage feature-driven model development workflow

Our objective was to maximize the area under the receiver operating characteristic (AUROC) curve in developing our machine learning models, focusing on a logistic classifier with an L2 penalty and a LightGBM model. We used True Positive Rate (TPR) and False Positive Rate (FPR) at varying classification thresholds, with the metric for TPR defined as:


(3) \begin{eqnarray*} TPR = \frac{TP}{TP + FN} \end{eqnarray*}

and for FPR as:


(4) \begin{eqnarray*} FPR = \frac{FP}{FP + TN} \end{eqnarray*}

The logistic classification model with L2 penalty was subjected to a Bayesian optimization hyperparameter search using the Optuna algorithm (version 3.4.0) (45) with 50 hyperparameter combinations trialled. For reproducibility, we followed the documentation suggestions, fixing the seed in PYTHONHASHSEED to 0. Briefly, Optuna explores broadly within the specified hyperparameter ranges, before the Gaussian process of Bayesian optimization takes over. Here, Optuna uses the tree-structured Parzen Estimator (TPE) to sample the next hyperparameter values based on the history of previously evaluated trials. It does this by calculating the ‘expected improvement’, which expresses how much the objective function can improve based on the suggested input values. Additionally, Optuna employs a ‘Hyperband’ pruning strategy that stops the underperforming trials early, allowing more focus on promising trials. The optimization process selects parameters across each of the following ranges of hyperparameter values. The regularization strength: from 1 × 10^−2^ to 1, tolerance: from 1 × 10^−6^ to 1 × 10^−3^ and maximum number of iterations: from 100 to 1000. The hyperparameters that yielded the best performance were found to be the following. The regularization strength: 1.629 × 10^−1^, tolerance: 4.551 × 10^−4^ and maximum number of iterations: 995.

The LightGBM model architecture was also subjected to the same Bayesian optimization hyperparameter search (45), sampling from the following search grid. The regularization terms were uniformly sampled in a logarithmic scale, biasing the search towards smaller values, but the resulting values were re-converted back to the original domain. The alpha: from 1 × 10^−8^ to 10.0 (log-scale), lambda: from 1 × 10^−8^ to 10.0 (log-scale), number of leaves: from 2 to 100, learning rate: from 0.001 to 0.1, number of estimators: from 1000 to 10000, feature fraction: from 0.1 to 1.0 and bagging fraction: from 0.1 to 1.0. The hyperparameters that yielded the best performance were found to be the following. The alpha: 7.483, lambda: 1.474, number of leaves: 83, learning rate: 9.836 × 10^−2^, number of estimators: 9952, feature fraction: 0.347 and bagging fraction: 0.475.

We evaluated the final trained model on unseen test data with the predict_proba(), roc_curve() and auc() functions from Python’s sklearn library. To calculate the accuracy, the predicted probabilities assigned to each class were converted to binary classification using a default probability threshold of 0.5. In each model, we evaluated the relative importance of each of the used features. For the logistic classification model, we obtained the magnitude and sign of each feature’s coefficient as a proxy for its importance. For the LightGBM model, we obtained them directly from the decision tree structure inherent to these models.

### DNA breakpoints at cancer-linked sites

We retrieved all the somatic mutation data of both the non-coding and coding regions associated with cancer from the Catalogue of Somatic Mutations in Cancer (COSMIC) database (http://cancer.sanger.ac.uk/cosmic, Non-Coding Variants, Coding Variants, Cancer Gene Census, Structural Variants [SVs], and Classification datasets obtained from release v98, May 2023). We excluded all single-nucleotide polymorphisms (SNPs), leaving us with insertions, deletions, deletion-insertions, duplications and inversions, where we treated each breakpoint independently. Each mutation was uniquely identified by combining the following identifiers: chromosome location, breakpoint location, primary tissue, primary cancer, mutation type and sample ID. The final dataset was comprised of 2,154,251 unique entries, including 441,790 SVs, 1,506,397 non-coding and 206,064 coding entries.

The filtered dataset held 194 unique pairs of primary tissue (T) and primary cancer (C) types, which we examined in combination as a cancer type identifier for each mutation. However, the distribution of the TC pairs was not balanced within the dataset. As a result, we focused our analyzis only on those pairs that constituted 95% of our filtered dataset entries. The resulting 19 TC pairs are outlined in Supplementary Table S2, where any subsequent analyzis was done on these pairs. Grouping the data based on the chromosome and position of a break, we found that certain positions tend to be persistently (breakage persistence, Bp) broken across multiple TC pairs.

### Lifting genomic coordinates to the telomere-to-telomere human genome assembly

The COSMIC-reported strand breaks were based on the hg38 genome assembly. We lifted them over to the more comprehensive T2T human genome assembly, which includes an additional 8% of the genome comprised of highly repetitive DNA sequences, and generally improving the quality of previous sequencing errors (36). Thus, to have a more comprehensive genomic and genic landscape, we lifted the hg38 genomic coordinates over to the T2T genome version using the UCSC CHM13v2 chain files *via* the liftOver() function from the rtracklayer library.

### Annotation of genomic and genic features on the T2T assembly

We obtained gene and repeat annotations for the T2T genome from the T2T Consortium (http://github.com/marbl/CHM13), housekeeping genes from the HRT Atlas (http://www.housekeeping.unicamp.br), and chromosomal fragile sites from HumCFS (http://webs.iiitd.edu.in/raghava/humcfs). We also included specific repeat classes, cancer driver genes from COSMIC release v98, May 2023 http://cancer.sanger.ac.uk/cosmic, CpG islands and isochores from the UCSC Genome Browser Table (http://genome.ucsc.edu/cgi-bin/hgTables). All the downloaded datasets that were not mapped to the latest T2T human genome version were subsequently lifted over following the aforementioned process.

### The generalized DNA fragility model

The LightGBM model was subjected to the same Bayesian optimization with 100 hyperparameter trials with 3-fold cross-validation (CV) using the StratifiedKFold() function from Python’s sklearn library 1.3.1 (http://scikit-learn.org). The 3-fold CV process, in its single fold, allocates 2/3 of the training dataset for the main training purposes, while 1/3 of the dataset was designed for internal testing. However, this internal testing should not be confused with the separate 30% of the data set aside for external testing. The optimal set of hyperparameters were: alpha 0.229, lambda 0.785, number of leaves 93, learning rate 9.271 × 10^−2^, number of estimators 9941, feature fraction 0.334, and bagging fraction 0.178.

### Calculating the probability of strand breaks per base

For any genomic feature, we calculated the total number of predicted DNA strand breaks across all autosomes, separately for each of the four thresholds applied. The percent of overlap was obtained by dividing this result by the total length of the genomic or gene annotation in focus, to obtain the probability per base. To quantify the relative fragility, we divided each probability by the maximum probability.

### Hot and cold fragility zones in the human genome

The human genome was binned into non-overlapping 1,960 base, 10 kb and 20 kb intervals (as region resolutions). We classified these intervals into high, low, and medium fragility zones, for each resolution, based on the distribution of predicted fragility by taking the top 5%, bottom 5% and the remaining ones, respectively.

### Sampling control strand breaks for hot and cold fragility zones

We randomly generated 10 million control strand breaks across the 22 autosomes, examining their intersection with the three fragile zones in each binned interval. We calculated the proportion of control strand breaks within each of the low, medium and high fragile zones as a fraction of the total number of control strand breaks.

### Analyzing ClinVar structural variants

We obtained SVs from ClinVar (http://www.ncbi.nlm.nih.gov/clinvar) accessed on 6 December 2023 that had a clinically associated pathogenic or benign label. We processed a 1,960 base sequence context centred on the SV occurrence. Each SV type was carefully processed to extract the ‘before’ and ‘after’ genomic sequences. For SVs, we predicted the sequence context fragility at every base position in a sliding window of one, while for single-nucleotide variants (SNV), we predicted its fragility centred on the location of the SNV (see our GitHub repository for details).

### Viral sequence fragility

We retrieved 1,376,446 DNA virus sequences from the Reference Viral Database’s (RVDB) clustered nucleotide sequence file, accessed on 6 December 2023 (46). We processed the retrieved dataset to only keep sequences that were annotated as a complete genome or sequence, and removed viral sequences that had keywords associated with various genomic or partial sequences of the complete virus. The final table contained 10,841 individual entries of DNA virus sequences. Each sequence was padded and analyzed using our fragility model to predict its DNA fragility. We categorized these viruses based on whether they infect endothermic and ectothermic animals (47), using species names mapped to common names through the taxonomizr library 0.10.6 (http://cran.r-project.org/package=taxonomizr). The detailed filtering process can be found in our GitHub repository.

## Results and discussion

### Design and workflow of the study

The first stage of the research aims to extract the most comprehensive sequence-based features of different DNA breakage phenomena from various processes (spontaneous, induced, physiological, pathological, and the natural decay and fossilization process from ancient DNA fragments), tissues and cell lines into *k*-meric properties that are fully independent of any genomic location. As such, there are no limitations on the number or distribution of samples used across the different DNA breakage phenomena, as feature selection and machine learning will handle this. Hence, for this purpose, we retrieved all the publicly available datasets, 100 in number (Figure 1A). To examine the role of the range of sequence context in DNA fragility, we aligned strand breaks to their cleavage origins within these datasets. We then compute normalized frequencies of *k*-mers and assess the variation between adjacent positions using the RMSD metric. This reveals the *k*-meric variation (sequence-based patterns) in the region surrounding the breakpoint. We hypothesize that sequence composition and context significantly influence DNA fragility. This is evidenced by an RMSD peak at the central breakpoint that heralds inherent compositional preferences, which gradually decay into background levels further away from the breakpoint. This decay is crucial, as it signifies the absence of sequence patterns influencing DNA fragility at farpoints. It also represents the background noise to which the signal is expected to converge at a certain distance from the central breakpoint location. Focusing on the *k*-mers that define the RMSD peak, we evaluated their breakage susceptibility and compared these patterns across various DNA fragility phenomena. We applied a similar methodology to extract *k*-meric propensity scores from chromatin, epigenetic and structural data (Figure 1B). These *k*-meric properties are summarized into a comprehensive feature library (DNAfrAIlib), designed for seamless integration during the feature generation phase of sequence-based machine learning models. The introduction of such engineered features can also enable these models to become, in part, interpretable, with insights on biochemical, mechanical and physical factors contributing to DNA fragility in a sequence-dependent way, offering novel insights into the genomic code of our genome with potential for broader applications (Figure 1C).

### Common patterns in DNA fragility

We found that all types of DNA breakages show a distinctly shaped RMSD signal within a 1 kb window around the breakpoint site, indicating non-random sequence-driven influences on DNA breakages (Figure 2 and Supplementary Figure S3). To validate the presence of a true positive RMSD signal in a negative control study, we randomly sampled breakpoints (Supplementary Figure S4), showing that the RMSD peak is absent, hence pointing to the non-random nature of DNA breakpoints and their genomic sequence determinants. However, the value of the RMSD is less important than the direct contrast between a strong signal and its background consolidation. Notably, the RMSD value converges to the background level well within the 1 kb genomic context window, indicating that extending the analyzis beyond this range is not necessary for the purpose of extracting the sequence range effects.

Within a 1 kb window, these patterns can be explained by three decoupled range effects, short-, medium- and long-range, by fitting up to three separate normal distributions to the signals (Supplementary Figure S1). The cumulative outcome of these effects forms the full-range sequence influence, where the peak of each curve defines its contribution to their combined effect (Figure 2; Supplementary Figures S3, S5–S9). The dominant sequence effect on DNA fragility is the short-range one, within a 13 ± 2 (μ ± σ) base window around the breakpoint, expectedly pronounced in the cases of enzymatic cleavages (Figure 2A, left). These effects are likely due to intrinsic sequence properties of the immediate vicinity to the breakage location, as well as to the DNA–protein interactions that normally involve a DNA segment of a similar span (48). Medium-range effects vary in range (μ ± σ is 71 ± 15 base window) but not significantly in their contribution to the full-range effects (Figure 2A, middle). They may prevalently be associated with the potential of a DNA to form various secondary structures (49), and with its hybridization dynamics that may also influence DNA fragility. Long-range effects (μ ± σ is 473 ± 456 base window), though less contributory (Figure 2A, right), are observed in various physiological processes and may relate to regional DNA packing, exposure and nucleosome positioning (50). However, the sequence range effects are highly dependent on the process, condition and cell type under which the DNA strands break. Hence, the aggregate results, as in the case of the long-range sequence effects, can display a large variance around the 473 base window average range effect. As such, the three range effects should be discussed in a general context instead of summarizing them into a specific value, unless discussing similar DNA breakage phenomena.

Beyond the distinctly different biological underpinnings within each range effect, the decoupled ranges also serve as reference points for extracting meaningful sequence-based features for subsequent downstream modelling tasks. Hence, range segmentation is a necessity for feature engineering. As we demonstrate in later sections, the individual ranges are nested, meaning that features derived from longer sequence range dependence capture the shorter segments of the same sequence.

We identified six distinct clusters based on their full-range sequence effects (Figure 2B) and found that each cluster can be described as being influenced by 2-3 decoupled range effects (Figure 2C). Clusters 1 and 3 represent physiological breaks and cluster 2 includes high-frequency breaks, including mechanical, natural decay of ancient DNA and cfDNA. As expected, the enzymatic cleavages form standalone clusters (clusters 4–6) given the nature of the induced single- or double-stranded DNA cleavages at specific sequences, often performed *in vitro* under controlled conditions. These cleavages are mainly driven by the short-range effects, which are also the most significant across all fragility phenomena (Figure 2D–E). Medium-range effects are notable in some physiological breaks, high-frequency breaks and the Nt BbvCI endonuclease in cluster 5, while long-range effects are observed in all conditions except those induced by the EcoRV enzyme. These results reinforce the necessity of separating the labels of specific enzymatic cleavage datasets from other physiological DNA breakages when performing aggregate analyzes, though the compendium of our features is not dependent on breakage class labels (see later sections).

### DNA shape explains contrasting sequence effects in enzymes

Out of the six unique clusters, three clusters are defined by single-member enzymes. The EcoRV enzyme, which targets the GATATC sites, shows a unique short-range effect in contrast to the broader influence of the Nt BbvCI endonuclease, which recognises CCTCAGC sites (Figure 2C, clusters 4 and 5, respectively). Given that enzymes mostly employ a ‘bind-slide-hop’ mechanism to interact with DNA (51,52), the contrasting sequence effects may reflect different DNA dynamical properties in the regions they encounter. By comparing the regional flexibility of the enzyme binding sites with the DNAShapeR software (40,53,54), we found that the Nt BbvCI enzyme operates in more flexible DNA regions, while EcoRV acts in more structurally stable areas (Supplementary Figures S10– S11).

G-quadruplex (G4) structures contribute to genomic instability by, among many effects, blocking transcription, resulting in replication fork stalls, and interfering with DNA–protein interactions (reviewed in (55)). Their co-localization with the medium-range GC-rich sequences associated with the Nt BbvCI cleavage sites presents a potential influence on the formation of DNA cuts. By isolating the effect of the G4 structures on the enzymatic cleavage propensity, we found that both enzymes show higher cleavage propensity near G4 structures in the A (open) genomic compartment, though more pronounced effects are seen by the EcoRV enzyme (Supplementary Figure S12). Thus, the contrasting sequence effects between EcoRV and Nt BbvCI enzyme may be influenced by the DNA sequence context, structural features (G4s), and their intrinsic cleavage properties.

### Intrinsic sequence effects are not influenced by gene elements

Physiological strand breaks often occur near gene elements, influenced by various protein machinery (reviewed (56)), which may be brought while involved in gene regulation. To isolate the sequence effects from any potential add-on influence from gene elements, we removed strand breaks coinciding with promoter sequences from the UCSC Known Genes dataset (57) (Supplementary Figure S13). As we quantified the sequence effects within a 1 kb window of the central breakpoint location, we also removed any overlaps that fell within this range (Supplementary Figure S11). Focusing on only the remaining breaks, we find that a characteristic peak at the breakpoint site remains (Supplementary Figure S14). We also repeated the same process for one representative example of each breakage class and revealed similar results. Thus, our results demonstrate that sequence context remains a key driver of DNA fragility, independent of gene elements.

### Compendium of short-range sequence propensities to DNA fragility under various conditions

Given the strong influence of the short-range sequence effect on DNA breakpoints, we narrowed our focus on this range effect. Hence, core to this study, we quantified the short-range breakage propensities and revealed six unique clusters based on similar fragility scores across various physiological, pathological and spontaneous conditions. These *k*-meric properties are summarized into a comprehensive feature library (DNAfrAIlib), designed for easy integration during the feature generation stage of any sequence-based machine learning task. We focused on processing and detailing a maximum short-range effect of eight bases (even-numbered for breakages), given the previously observed significance of the heptameric range on spontaneous mutation rates (3,4,43). We ensured computational efficiency in processing all possible *k*-mer combinations (65,536 octamers in total or 32,896 octamers after accounting for strand symmetry). To that end, we quantified the intrinsic fragility by comparing the population of broken *k*-mers with a negative control population sampled beyond the long-range sequence effects of the broken genomic loci to minimize sequencing biases. To account for regional sequencing variations, we corrected for potential biases while still sampling nearby regions to capture local non-uniformities (see ‘Materials and Methods’ section). An alternative approach would be to compare the broken *k-*mer population with the genome-wide background *k*-mer frequencies; however, this would significantly hamper the accuracy and significance of the resulting *z*-score values. If no sequence context effect is found, no *k*-meric sequence would have any significance associated with it. An example of this is the lack of an RMSD signal in Supplementary Figure S2, which would equally have no intrinsic fragile *z*-score value. Intriguingly, despite a strong correlation in *k*-meric fragility scores within replicas of the same experiment, a comparison of their exact breakage sites revealed a mere 3% average overlap (Supplementary Figure S15). These results highlight a consistent, probabilistic bias for certain DNA sequences to be more prone to breakages.

We created a heatmap to show the *k*-meric profiles across all types of DNA breakages (Figure 3A) revealing two main findings. First, *k*-mers with similar fragile properties are grouped (Figure 3A, columns), showing consistent profiles across various conditions (Figure 3A, rows). Second, *k*-mers that are fragile under one group of conditions can be highly resistant under another. For example, GC-rich k-mers resist breakages in highly fragmented DNA breakage conditions (mechanical, ancient DNA and cfDNA) (cluster 1 in Figure 3A and B) but are vulnerable to some physiological breaks (cluster 2 in Figure 3A and B). Here, we found six clusters with distinct *k*-meric fragility scores in DNA and revealed common patterns in the hybridization dynamics of DNA sequences. We focused on the top 1% of *k*-meric differences in fragility scores and compared their hybridization energies, revealing that fragile *k*-mers require less energy to de-hybridize than the most resistant ones (Figure 3C and Supplementary Figure S16). Our findings establish a link between DNA fragility, thermodynamic stability and conformational flexibility, with consistent results across all clusters except for 5 and 6, which share similar high-fragmentation breakage mechanisms. We performed quantum mechanical (QM) calculations to obtain the change in the heat of formation energy of duplex B-DNA in different sequence contexts (43). We used these results as a proxy for the DNA hybridization energies. In comparison, the experimental triplet hybridization energies (42) were approximated to the octameric sequence length *via* a one-nucleotide sliding window and averaging approach. Both approaches lead to highly correlated results (Pearson and Spearman *R* at 0.89, Supplementary Figures S16 and S17), suggesting that our QM approximations are consistent for hybridization energies.

Next, we quantified the *k*-meric susceptibility scores from various chromatin, epigenetic and structural features (Figure 1B), and correlated them against various DNA fragility phenomena in a network graph, revealing three major clusters and some minor clusters. Cluster C1 is the largest cluster which expectedly groups most of the transcription factor binding sites (TFBSs) together (Figure 3E). Interestingly, five DNA breakage samples are associated with this cluster, in which the depletion of WRN helicase led to (TA)_n_ dinucleotide repeat expansion and subsequent chromosome shattering (32). Cluster C2 (Figure 3E) associates TFBS with specific adenine/thymine-rich motifs and the change in the heat of formation energy of duplex B-DNA, suggesting that certain TFs may prefer DNA regions with higher conformational flexibility (58), facilitating easier access to their binding sites and enabling more efficient gene regulation in response to cellular signals. Cluster C3 (Figure 3E) shows a correlation between cfDNA from cancer cells and chromatin features, including chromatin accessibility, regulatory regions and histone marks, suggesting that higher nucleosome density protects DNA from degradation (50).

### Sequence-based model development from DNAfrAIlib features

As an example and a usability test, we incorporated unique features from short-, medium- and long-range effects to develop a machine learning model and classify DNA fragility. These data are obtained from DMSO-treated, endogenous DNA breakages in K562 cells (59), which is a similar dataset also used in a previous study (23), at a single-base resolution. We employed two different model architectures, the logistic classifier and the tree-based gradient boosting machines (LightGBM flavour of GBMs), to first examine how model complexity influences the predictive power of our feature library. The gradient boosting process entails an ensemble of learners that is developed with each iterative learner focusing on the residual of the ensemble of prior learners. GBMs offer an improved performance, and a wide range of tunable hyperparameters, compared to traditional tree-based methods (e.g. decision trees and random forests) due to the combination of the decision tree component (maximum interaction depth and number of leaves) and the gradient boosting component (number of boosting rounds, learning rate, bagging fraction and feature fraction). Unsurprisingly, tree-based GBMs are amongst the top-performing machine learning methods, particularly for feature-based and tabular data (60,61), as seen in many machine learning competitions, such as Kaggle (www.kaggle.com). GBMs are also attractive because we can extract information regarding the relative importance of each input feature in predicting DNA sequence fragility, thus enabling better interpretability of the model.

We extracted sequence-based features across the different ranges, avoiding any overlap between the true and negative control breakpoints (see ‘Materials and Methods’ section). Short-range features included the intrinsic *k*-meric susceptibility scores from various DNA fragility phenomena, TFs, changes in the heat of DNA duplex formations, G4-seq confirmed structures and epigenome marks. For this example and usability test, we removed the *k*-meric fragility scores from the feature set to avoid any potential data leakage. Medium-range features included DNA shape parameters and the potential to form G4 structures *via* a simple regular expression search, which also searches for *i*-motifs due to the complementary nature of their motifs. Finally, the features of the long-range effects included the G+C content, GC skew and triplet *k*-mer counts. The final feature matrix contained 421 purely sequence-based features with 39% true and 61% negative control breakpoints as the response value, which we partitioned into 70% training and 30% testing sets.

### Strong features can improve predictive power in sparse data environments

Here, as a demonstration, we show that our *k*-meric fragility scores are more predictive, and arguably more interpretable, than simple *k*-mer counts as features for machine learning. We trained the logistic and LightGBM classifiers on the same training data, tested them on the same test data and compared the *k*-meric fragility scores to triplet *k*-mer counts within identical sequence contexts. We first examined the effect of data sparsity, by downsampling the training data from 1.5k to 500k samples, showing the consistently improved performance from the *k*-meric fragility scores over triplet counts across both model types (Supplementary Figure S18, average differences: logistic classifier 1.9% AUROC and 1.1% accuracy; LightGBM 0.7% AUROC and 0.6% accuracy).

Next, we combined all features within the short-, medium- and long-range sequence effects to demonstrate the overall strong performance. We optimized each model using a Bayesian optimization hyperparameter search *via* Optuna (45) (see ‘Materials and Methods’ section). The optimized logistic model achieved an AUROC of 0.811 and an accuracy of 0.755, with *k*-meric fragility scores contributing significantly to feature importance (Supplementary Figure S19). Similarly, the optimized LightGBM model reached an AUROC of 0.853 and an accuracy of 0.785, with *k*-meric scores forming the majority of influential features, while triplet counts had a minor contribution (Supplementary Figure S20). These results further highlight the robustness of our generalized feature engineering approach and its suitability as a supplement for any future modelling endeavours, where accounting for DNA fragility may be useful.

### Towards a generalized fragility model from all cancer-associated DNA strand breaks

Mourad *et al.* developed a random forest model, which utilises a combination of epigenomic and chromatin markers to predict double-strand break (DSB) tracks with a resolution of under 1 kb (22). This model highlighted the role of chromatin accessibility, activity, and long-range contacts in determining DSB sites while using such extra information directly in the modelling process. Overall, machine learning techniques are increasingly being leveraged to understand structural variations (SVs) within the genome given their prevalence in various types of cancer. As such, genome-wide studies of SV breakpoints have been carried out in the past (7,21), but the application of machine learning in modelling DNA DSB susceptibility to study SV has been limited. Addressing this gap, Ballinger *et al.* examined the human genome in large non-overlapping intervals of 50 kb windows and used DSB frequency data of three separate cell types to predict genome-wide susceptibility to DSBs, offering new insights into the genomic landscape of SVs and their implications (23).

Here, we used the same LightGBM modelling approach that we adopted above, this time training on diverse cancer-associated strand breaks, hence moving beyond any specific cell line or mechanism, to help us better understand the intrinsic biophysical, chemical and mechanistic properties of sequence fragility. Most of the studies and deposited genomic information in cancer do not represent a uniform sampling and representation of the complete human genome but rather have ‘focus spikes’ at genomic regions of functional and historic interest. Here, by flattening the accumulated cancer-associated DNA breakage data and developing a general sequence-driven model, we aim to assess the fragility throughout the whole human genome, adding information for the regions not well covered by sequencing in cancer.

We first analyzed the cancer-associated DNA strand breaks across deletions, insertions, indels (insertions and deletions), duplications, inversions and chromosomal translocations from the Catalogue of Somatic Mutations in Cancer (COSMIC) database (http://www.sanger.ac.uk/cosmic). Interestingly, we found persistent breaks forming across various tissue and cancer (TC) combinations, for instance, with up to 25 unique combinations sharing the identical breakage location on chromosome 17 (Supplementary Figures S21–S22 and Supplementary Table S2). We also found that approximately 80% of strand breaks are located within 10 base pairs of each other (Supplementary Figure S23). These results show promising implications for the following machine learning phase, enabling the extraction of distinct features from strand breaks and the setting up of negative control regions far enough to avoid any potential sequence context influence.

We made some modifications to the feature extraction process to balance granularity with computational feasibility. Within the medium-range span, computationally intensive DNA shape parameters and secondary structure calculations were omitted for efficiency. Within the long-range span, we substituted triplet *k*-mer counts with pentamer counts, and used hexameric intrinsic fragility scores. The final feature matrix consisted of 2,681,326 data points with a balanced ratio of 44% true and 56% negative control breakpoints and 640 features. The model underwent the same Bayesian optimization hyperparameter search grid with 3-fold CV, aiming to maximize the AUROC (see ‘Materials and Methods’ section, Supplementary Figure S24). The model achieved strong predictive performance on the unseen test data with an AUROC of 0.899 (Supplementary Figure S25). However, we ought to stress that the AUROC of the model only indicates the potential effectiveness in binary classification tasks. Our goal is to employ the model for the entire human genome sequence, and any sequence or sequence modification in the human cell nucleus, where the experimental data, as used in the training and testing phases, might not be readily available. By default, the machine learning model assigns probabilities to each class, employing a standard 0.5 threshold for each class. To that end, we set four different stringency thresholds based on the target FPR from the testing phase (Supplementary Table S1). The top 25 features are mainly driven by the *k*-meric breakage susceptibility scores (96% contribution) and the top 20% of features are equally contributed by the *k*-meric breakage scores and pentamer counts (Supplementary Figure S25). This demonstrates the capacity of the model to leverage complex feature interactions for high predictive power.

### Sequence-driven fragility of various genomic features

Here, we first examine the COSMIC-reported strand breaks coinciding with genomic features in the human genome. We found that the coding sequences (CDS) have the highest relative fragility, followed by low complexity regions, overall exons (UTRs and CDS together) and CpG islands, while non-coding regions and various repetitive elements showed lower fragility (Supplementary Figure S26). However, these observations could be influenced by the inherent sampling bias in the COSMIC database focusing on particular genes, genomic regions (many studies from exome-only sequencing) and cancer phenomena.

In contrast, while deploying our model to predict sequence-driven fragility across the entire human genome, we can note that the genomic DNA regions corresponding to transcripts, telomeres, genes related to cancer and G-rich sequence spans in the form of heavy isochore regions, CpG islands and G4 sites, exhibit the highest sequence fragility relative to other genomic features (Supplementary Figure S26). The genomic DNA regions corresponding to transcripts may be particularly fragile due to the persistent stress induced by transcription and replication machinery known to elevate recombination and mutagenesis, as well as the formation of R loops (62). Interestingly, the H3 isochores and CpG islands, appear to be more fragile than G4 sites, suggesting that G-rich sequences, when formed into G4 structures, might become more stable, while the surrounding regions of these G4 structures seem to be where breakages are more likely to occur in line with experimental evidence (63,64). Our analyzis of the COSMIC database reveals that tumour suppressor genes, cancer driver genes and chromosomal fragile sites are more fragile than housekeeping genes, possibly reflecting the inherent sampling bias in the database, which already encompasses a comprehensive record of cancer driver and passenger mutations. In contrast, our model shows a reversal of these trends, with housekeeping genes being generally less prone to a break than the cancer-associated genes, even when we use high classification thresholds (Supplementary Figure S26). However, the proportion of these highly fragile sites is mostly a relatively small percentage, when compared to the overall fragility of each genomic feature, with most of them being close to their expected value.

### Insights into hot and cold fragility zones in the human genome

Here, we binned the genome into 20 kb intervals to examine the distribution of genomic features in hot and cold break zones. We find that regions that are rich in heavy isochores, G4 structures, and the overall CDS have a larger share of hot break zones, though their proportions are relatively small when compared to the overall fragility of each genomic feature (Supplementary Figure S27). Interestingly, a high proportion of tRNA loci are in hot break zones. These loci are known for their high mutation rates (65), necessitating extensive repair mechanisms that might contribute to DNA strand breaks. Genes within chromosomal fragile sites, which are large genomic regions susceptible to breakage under replication stress and visible as cytogenetic gaps or breaks in metaphase chromosomes (66,67), also showed increased fragility. Despite the overall high fragility of telomeres, their fraction in hot break zones is considerably lower compared to other genomic features with G-rich motifs, considering that telomeres have a highly conserved G-rich repetitive DNA sequence: the (TTAGGG)_n_ motif (68). These can form G4 structures that protect telomere ends and regulate telomerase access. The telomerase enzyme is expressed in normal cells but is notably dysregulated in cancer (reviewed in (69)).

### Sequence-driven fragility of genes, including those of transcription factors

Here, we examined the sequence fragility within cancer-related genes, focusing on driver genes, oncogenes and tumour suppressor genes. A comparative analyzis of genes across housekeeping genes, oncogenes, and tumour suppressor genes revealed that housekeeping genes carry significantly less fragile regions compared to oncogenes and tumour suppressor genes (Supplementary Figure S28). We also found that TSC1 is amongst the most fragile cancer driver genes (Supplementary Figure S29), which, when mutated, becomes hypersensitive to the accumulation of unfolded or misfolded proteins within the endoplasmic reticulum, impairing normal cellular functions and leading to cellular apoptosis (70). NOTCH1 gene is also particularly fragile (Supplementary Figure S29), which tends to be overexpressed in cancer-associated fibroblasts and suppresses ATM activation, allowing it to bypass the DNA damage repair pathway (71). PSIP1 is also similarly fragile (Supplementary Figure S29), where overexpression of the gene is associated with breast cancer by possibly modulating the interaction of RNA polymerase II with cell cycle gene promoters, thereby potentially enhancing their transcription (72).

Next, we extended our analyzis to 247 TF genes from the JASPAR 2024 database (73), crucial for regulating gene activity and maintaining genomic stability (74) (Supplementary Figure S30). Here, we aim to identify the top 5% of TFs that are most susceptible to DNA breakage, based on the predictions from our fragility model. Our model identified SOX10 as a highly fragile TF gene (Supplementary Figure S30), which is associated with glioblastoma and melanomas (75), and its deletion is believed to cause Waardenburg syndrome (76). The E2F TF family, which controls cell cycle progression, differentiation, metabolism and development, also emerges among the most fragile ones. E2F activity is normally controlled by the RB tumour suppressor, and inactivation of the RB protein or mutations in E2F can lead to an overexpression of E2F activity (77).

### The effect of sequence variants on regional fragility changes

**Figure 4.**
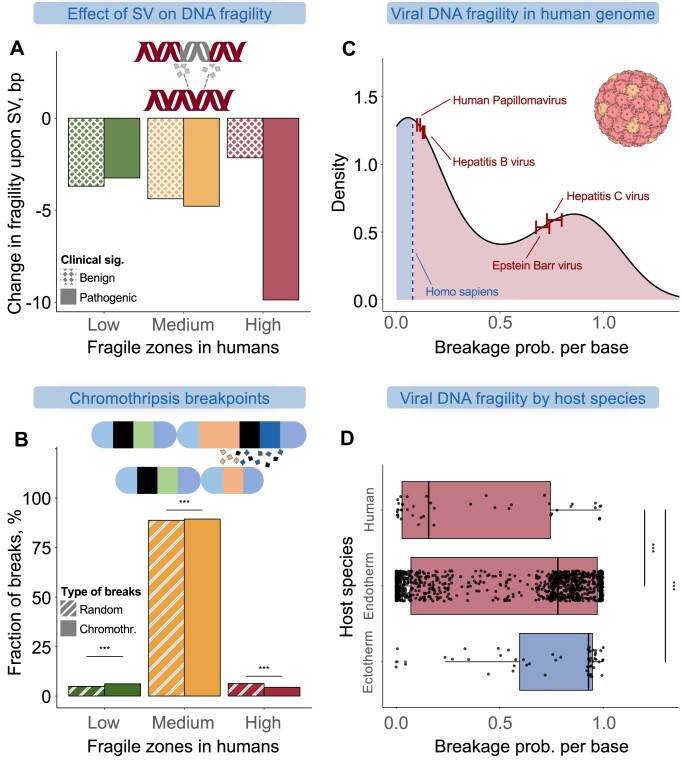
DNA fragility model applications for SVs, chromothripsis events, and viral sequences in host DNA. (**A**) All SVs seem to contribute to the stabilization of the given region, as indicated by the bar plots representing the average change in fragility upon SV occurrence. Interestingly, pathogenic SVs found in highly fragile zones decrease the regional fragility the most, potentially lowering the susceptibility to further SV occurrences. (**B**) Chromothripsis events tend to happen more in less fragile zones compared to highly fragile ones, particularly when compared to randomly sampled strand breaks across the human genome. We performed a *z*-test for a difference in the proportions between the chromothripsis breakpoints and randomly sampled negative control breakpoints. (**C**) Most RNA and DNA viruses in their DNA form exhibit significantly higher fragility while integrated into the human host, including the four cancer-associated viruses shown labelled on the plot. The blue shaded area under the curve, before the dashed vertical line, corresponds to DNA viral species that are more stable in the human host genome as compared to the human genome average, while the red shading, after the dashed vertical line, illustrates increased fragility upon integration into the human host. The crystallographic structure of the HPV (PDB entry 3j6r) is illustrated in the top-right corner. (**D**) Viruses originating from ectothermic species, if those were to be transfected to endothermic humans, tend to be highly fragile. We used all the available viruses that infect either endothermic organisms (baboon, blackbird, bulbul, canary, chimpanzee, crow, dog, douroucouli, finch, galago, giant panda, gibbon, gorilla, guereza, hedgehog, human, lark, loris, macaque, magpie, marmoset, mesia, monkey, munia, orangutan, pigs, rat, robin, serin, shrew, sifaka, sparrow, starling, tamarin and thrush) or ectothermic organisms (butterfly, crocodile, dragon, fish, frog, python, shrimps, snake, toad and turtle). The ectothermic and endothermic annotation of viruses is based on (47), where we mapped species names to the common names *via* the taxonomizr library in R. A two-sample *t*-test was performed between the virus fragilities in humans, for the viruses designated for humans (red) versus those designated for ectothermic (blue) and endothermic (red) animals. The statistical significance is annotated over the barplot (B) and boxplot (D) (***P < 0.001).

Here, we show that SVs tend to decrease the sequence fragility of the given region and contribute to their stabilization upon emergence. We analyzed unique 390,817 SVs from the ClinVar database (http://www.ncbi.nlm.nih.gov/clinvar), encompassing insertions, deletions, duplications, inversions, and translocations, and their effects on DNA fragility. Each SV’s surrounding 980 bp sequence was compared before and after the variant occurrence. This sequence context was chosen to stay consistent with the long-range sequence influence used in our earlier machine learning model development. The majority (67%) of these SVs were pathogenic, and the analyzis revealed that the difference between the pathogenic and benign SVs, though small, is statistically significant (Supplementary Figure S31). We also find a slight depletion of pathogenic SVs in low fragile regions compared to both benign and randomly sampled strand breaks (Supplementary Figure S32). When examining the distribution of clinically significant SVs across the genome, we find that, on average, all SVs stabilise the area upon emergence (Supplementary Figures S33 and S34). Notably, pathogenic SVs in highly fragile regions primarily drive this stabilization, whereas benign SVs tend to contribute similarly to the stabilization across all fragile zones (Figure 4A and Supplementary Figure S34). Importantly, these average behaviours are statistically significant for benign SVs in the high fragile zones, while pathogenic SVs are also significant in the low fragile zones. Interestingly, we find an inverse relationship between pathogenic and benign SVs in highly fragile regions, wherein pathogenic SVs tend to contribute increasingly more to regional stability while benign ones tend to increasingly destabilise it. We extended this analyzis to 1,975,113 SNVs, finding that most SNVs do not affect their fragility (Supplementary Figure S35), possibly due to our limited sensitivity of the model in isolating the impact of a single base within a nearly 2 kb considered region. The ones that are indeed captured by our model are likely a reflection of the most significant differences in the *k*-meric breakage susceptibility scores. However, further analyzing SNVs by their clinical significance reveals that pathogenic and benign SNVs tend to have, on average, twice as many SNVs that result in decreased fragility.

### Chromothripsis breakpoints within hot and cold fragility zones

Here, we show that chromothripsis sites, characterized by extensive chromosomal rearrangements in a destabilized genome (78), occur mostly in less fragile regions of the genome, as compared to randomly sampled DNA strand breaks (Figure 4B). Despite its significance (Figure 4B), it may not appear as striking. However, we note a consistent trend of chromothripsis breaks residing within less fragile zones across all four model classification thresholds (Supplementary Figures S36 and S37). As such, these results potentially allude to an evolutionary driving force for keeping chromothripsis events away from highly fragile sites. This analyzis was done using data from ChromothripsisDB, which includes over 400 documented chromothripsis events (79). Next, we categorized chromothripsis cases by disease and cancer type and showed that the most fragile chromothripsis events are associated with thyroid cancer (Supplementary Figures S38 and S39). Interestingly, chromothripsis-associated breaks in thyroid cancer are located on chromosome 9 and are rich in AT sequences, which tend to be associated with chromosomal fragile sites and are prone to form non-B DNA structures (32,80).

### Sequence fragility of various DNA and RNA viruses

Here, we studied the sequence fragility of 10,841 virus species from the Reference Viral Database (46) and found many that exhibit significantly higher fragility when integrated into a human host genome, including the known cancer-associated viral species (Figure 4C; Supplementary Figures S40 and S41). On average, this pattern still holds when also considering that multiple strains of the same virus are evaluated with similar but varying degrees of sequence fragility. Importantly, our model is trained on the complete human genome, hence, employing this model on viral sequences, therefore, assesses their potential fragility within the context of a human genome host. As such, all virus species were processed in their corresponding DNA sequence, allowing us to examine their general fragility and compare them to the human genome average, independently of the source virus and the mechanism of genomic integration. Viral infections are implicated in up to 20% of cancers (81), with notable examples including the Epstein-Barr virus, which can increase cell proliferation (82), the human papillomavirus (HPV) can inactivate tumour suppressor pathways (83), hepatitis C virus implicated in some B-cell non-Hodgkin lymphomas (84), and hepatitis B virus often integrates near the TERT gene (85), a site associated with high fragility.

Moreover, if a viral species is more fragile simply due to its longer sequence span, its integration into the host genome may contribute to a higher fragility than a short sequence. To that end, comparing the absolute number of strand breaks versus the relative fragility scores of each viral species shows that a number of viral species are not directly associated with any cancer types but are predicted to be highly fragile (Supplementary Figure S41).

We also found that viruses from ectothermic animals, if those were to be transfected into endothermic humans, tend to be significantly more fragile than those naturally evolved to infect (hence, thrive in) humans (Figure 4D and Supplementary Figure S42). The increased fragility could be evolutionarily advantageous for viruses, allowing rapid adaptation (reviewed in (86)) if within optimal pathogenicity for transmission and integration without immediate lethal effects on the host genome. This balance is crucial for the virus survival and propagation. Given that the development and progression of cancer far supersede the timescales of viral transmission and integration into host organisms (87), it is thus feasible for a DNA virus to evolve towards increased sequence fragility without hampering its survival or transmission efficacy. The bimodal nature (Figure 4C) likely reflects the variation of body temperatures in the endothermic species, which may be further clarified upon the availability and analyzis of body temperature data for a wide variety of species.

In conclusion, we identified three separable levels of sequence-based influences on DNA fragility: short-, medium- and long-range effects. We quantified the *k*-meric susceptibility to breakage, chromatin, epigenetic, and structural alterations. We then summarized these into the DNAfrAIlib feature library for seamless integration into machine learning models. Using these features, we developed a generalized DNA fragility model and found that SVs, particularly pathogenic ones, tend to stabilize the regional fragility upon emergence, while chromothripsis events tend to occur in less fragile regions. Finally, we found that viral integration into the human genome can potentially increase regional fragility, especially in the case of cancer-associated viruses and those originating in ectothermic species.

Our study provides a foundation for understanding the underlying sequence basis of DNA fragility, genome (in)stability and evolution. The deposited library of features can improve sequence-to-feature translation that emulates multitudes of DNA properties in machine learning initiatives. Overall, our study enables more targeted exploration and exploitation of intrinsically unstable regions in the human genome, particularly in the pursuit of revealing the underlying molecular mechanisms and potential treatments of various genetic disorders and cancers.

## Supplementary Material

gkae914_Supplemental_File

## Data Availability

All source data associated with this manuscript are publicly available as described in Materials and Methods. The developed DNAfrAIlib library of *k*-meric fragility features for machine learning is publicly available *via* the http://github.com/SahakyanLab/DNAfrAIlib GitHub repository. The kmeRtone programme is written and implemented in R and is freely available *via* the http://github.com/SahakyanLab/kmeRtone GitHub repository or from R CRAN (http://cran.r-project.org/package=kmeRtone). The computer code, necessary to process the DNA breakage datasets, calculate the intrinsic sequence influences and quantify the intrinsic *k*-meric propensities can be accessed through the following GitHub repository: http://github.com/SahakyanLab/DNAFragility_dev. All the scripts for the development of the machine learning models in this study can be accessed through http://github.com/SahakyanLab/DNAFragility_ML. The code has been additionally preserved in the Zenodo repository (88).
