# Supplementary material for: Towards the genomic sequence code of DNA fragility for machine learning
Source: Nucleic Acids Res. 2024 Oct 23;52(21):12798–816. doi: 10.1093/nar/gkae914 (PMC11602142; doi:10.1093/nar/gkae914)
Supplement: gkae914_Supplemental_File [file gkae914_supplemental_file.pdf]

Supporting Information

Towards the genomic sequence code of DNA fragility for machine learning

Patrick Pflughaupt, Adib A. Abdullah, Kairi Masuda, and Aleksandr B. Sahakyan

Contents

Supplementary Figures and Tables **2**

Supplementary References **42**

Supplementary Figures and Tables

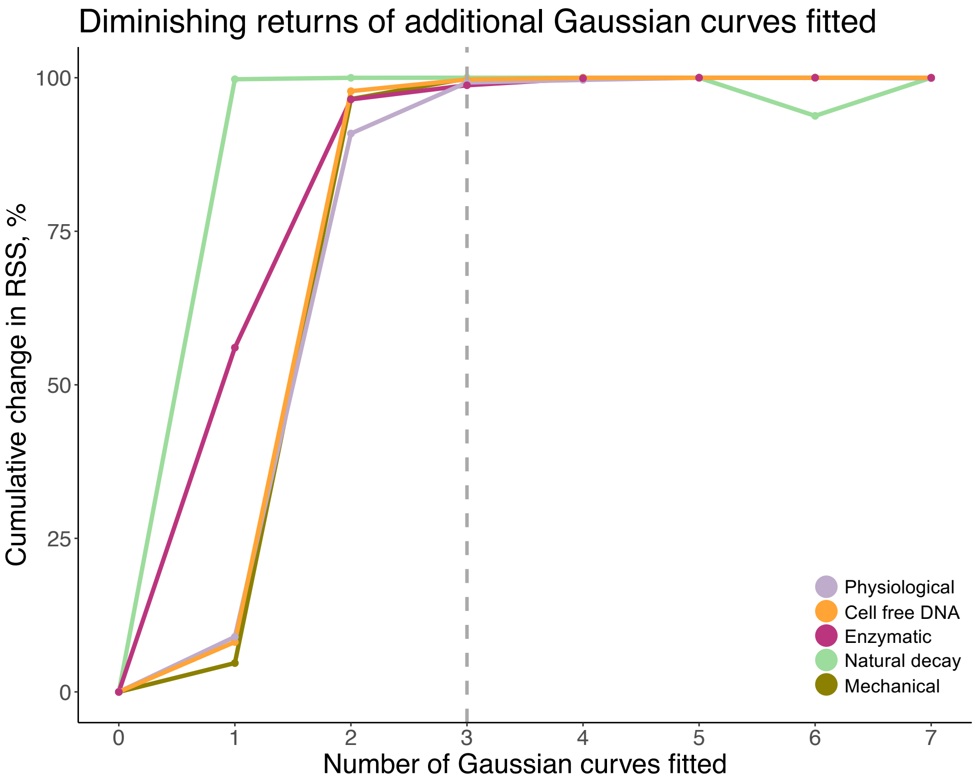

Figure S1. Diminishing returns of fitting additional Gaussian curves. *We selected one representative example dataset for each breakage class [1–5], and within this dataset, we iterated through one to eight Gaussian curves to fit within the defined range of -500 to +500, with the mean set at the breakpoint origin (see Materials and Methods for further details). As the initial conditions impact the convergence outcomes, we employed a grid search-based approach to refine the starting parameters for the coefficients. Upon convergence, we recorded the residual sum of the squares (RSS). To demonstrate the effectiveness of incorporating additional Gaussian curves, we computed the cumulative percent change in RSS. The results reveal the diminishing returns of fitting more than two or three curves, which is consistent with the expected biological effects in various short-, medium-, and long-range sequence-driven influences.*

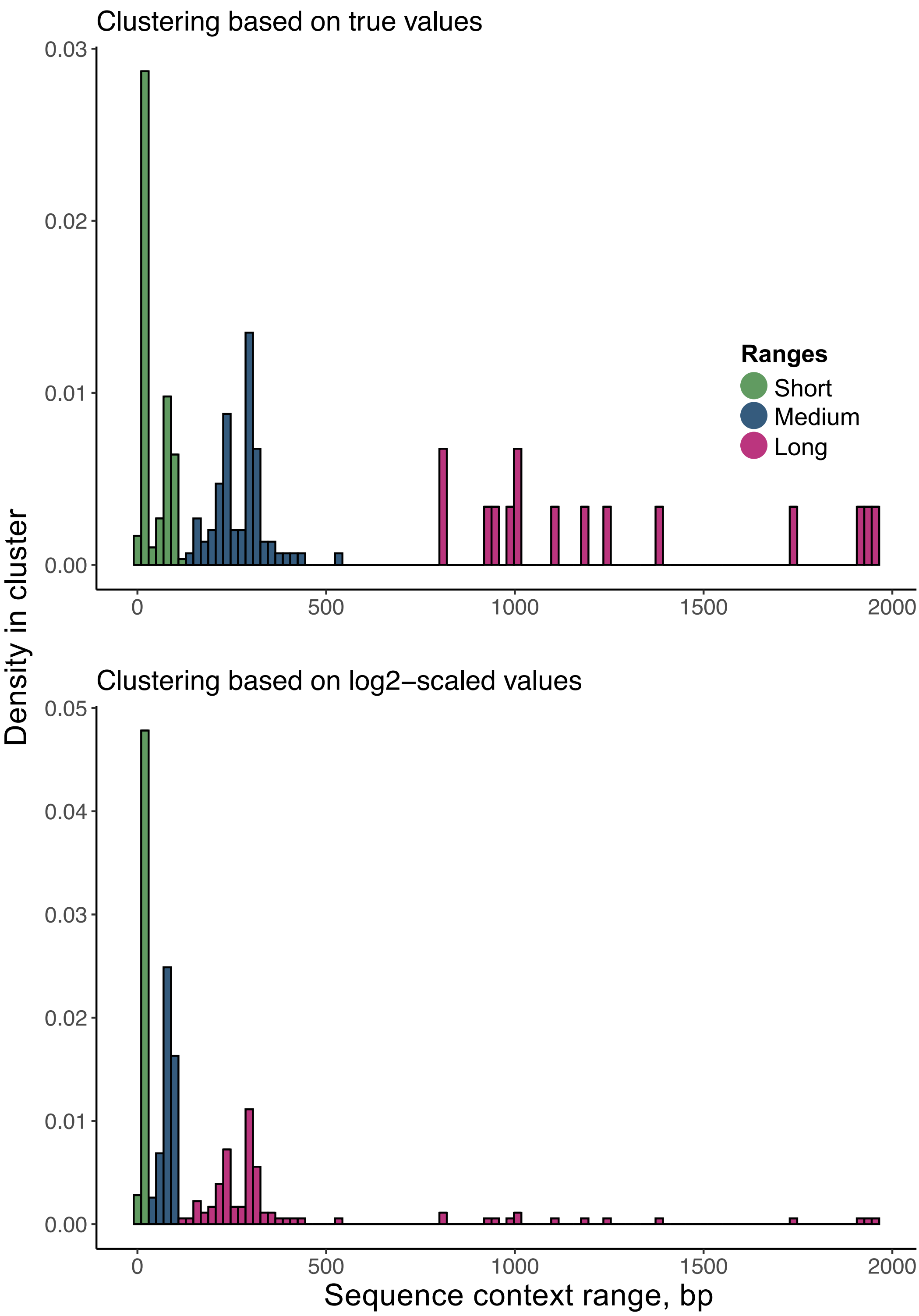

Figure S2. Clustering Gaussian curves into ranges of biological importance. *We fitted Gaussian curves to each unlabelled dataset, after which we normalised the data ranges. We then conducted hierarchical clustering using the Euclidean distance matrix with the `Ward` linkage method, and the dendrogram was divided into three distinct clusters to delineate short-, medium-, and long-range sequence-driven effects (**top**). Initially, the within-cluster value ranges lacked biological relevance. To address this, we followed the same clustering process using the log_2-transformed data and mapped them back to the true range (**bottom**).*

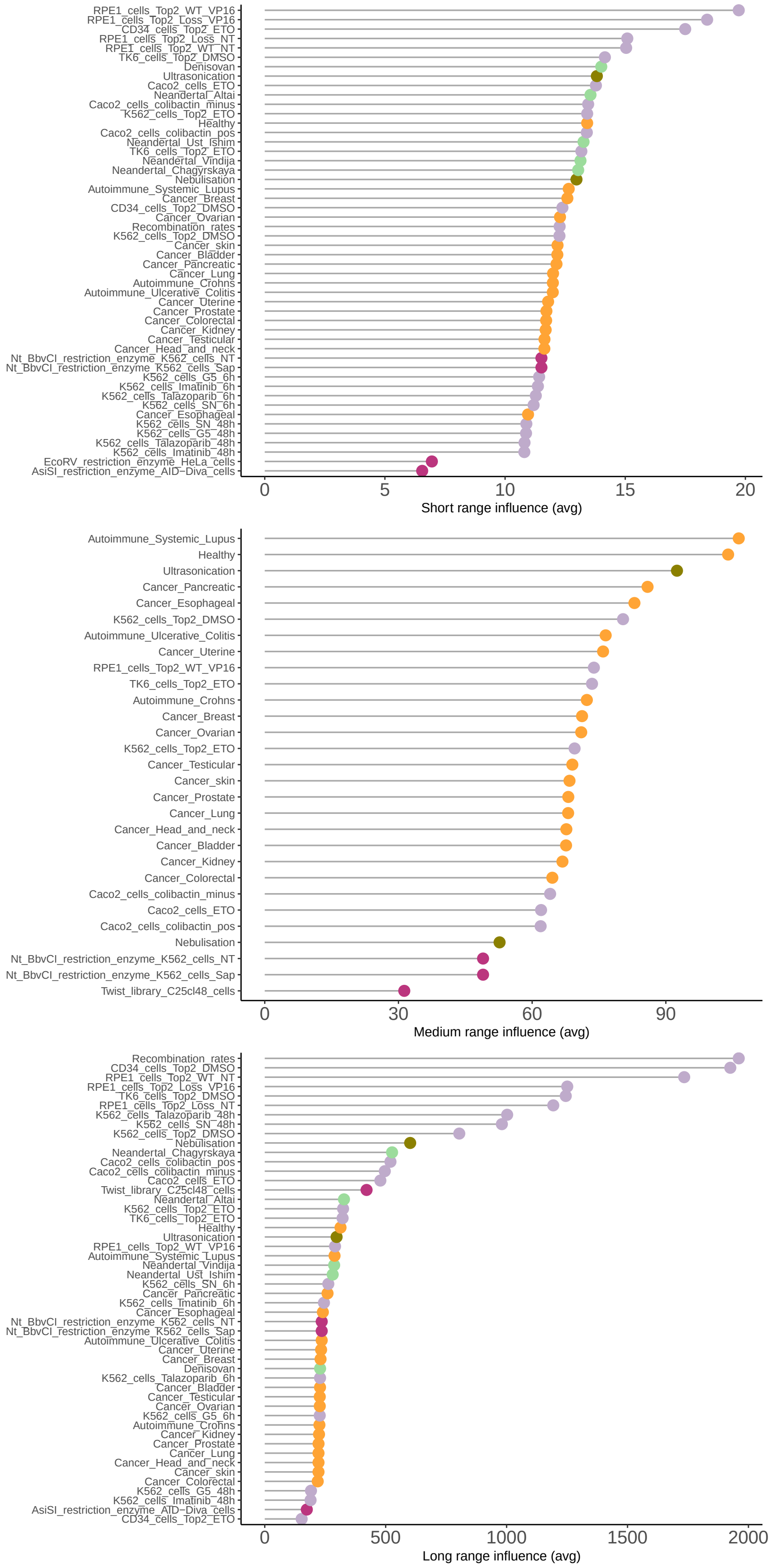

Figure S3. Sequence context influence of DNA fragility under various conditions. *The influences can come from immediate neighbouring effects representing the short-range effects **(top)**, medium-range effects **(middle)**, and long-range effects **(bottom)**.*

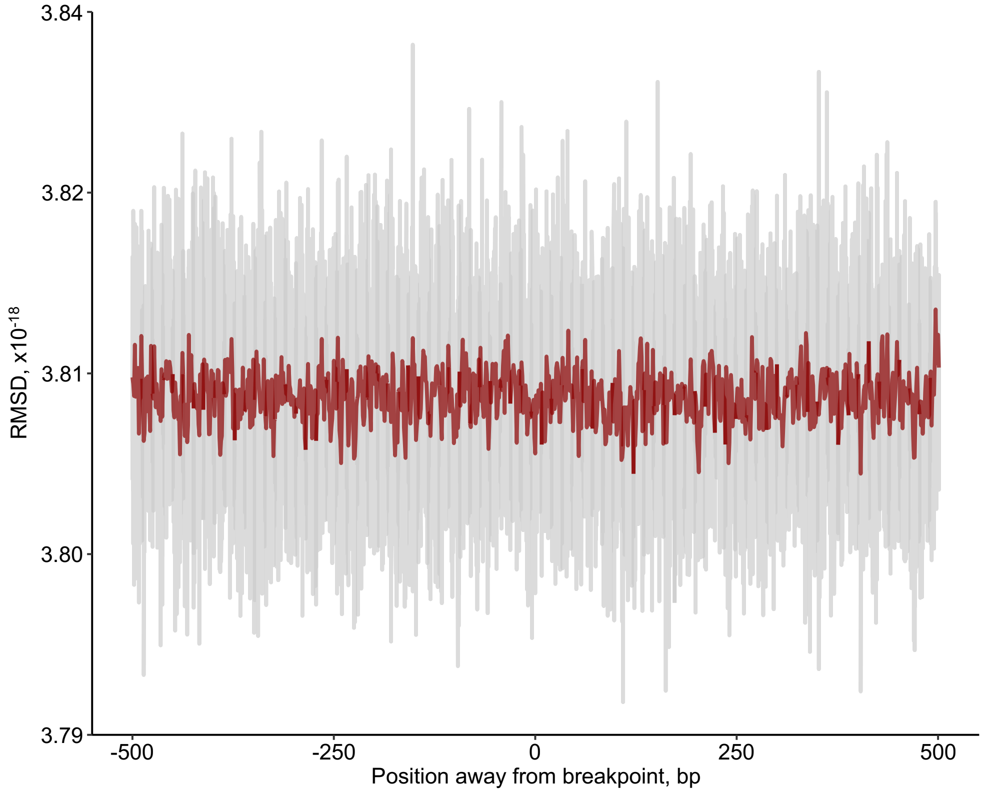

Figure S4. Characteristic peak is not present for negative control DNA strand breaks. *We randomly sampled 30 million negative control breakpoint positions and quantified their sequence effects within a span of -500 to +500 bases relative to the control breakpoint at the zeroth position. This was independently repeated ten times and visualised as the grey line with the position-wise averaged values in red. No signal is detected, suggesting that when the characteristic peak with asymptotic decay is indeed observed, then this indicates true positive sequence-based effects.*

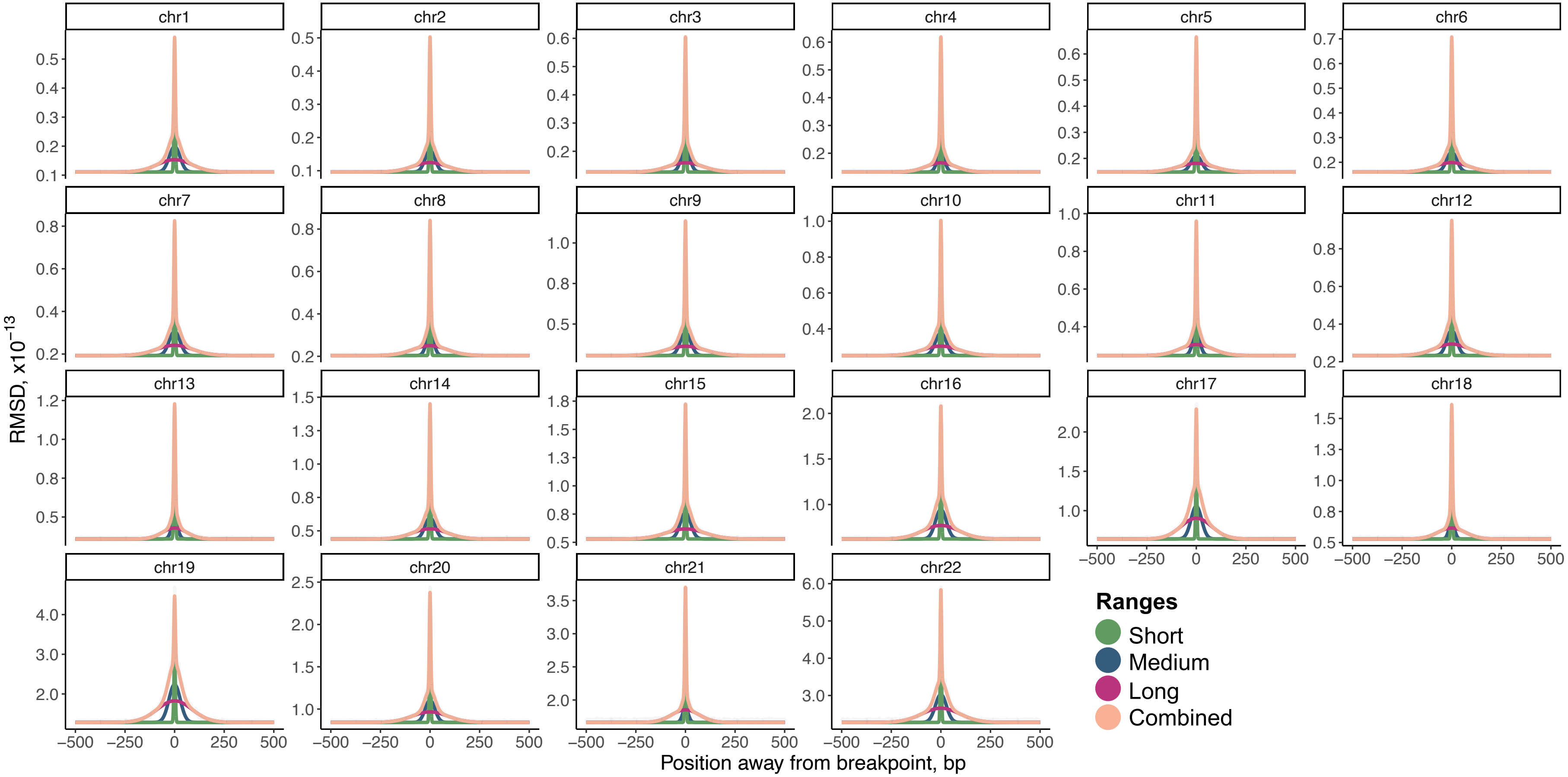

Figure S5. Quantifying the span of sequence-based influences for all autosomes of mechanical fragility. *We selected one representative example dataset for the mechanical breakage class [5], and calculated the range of sequence influence assessed from -500 to +500 bases relative to the origin of the breakpoint at the zeroth position. Gaussian curves were fitted to quantify the range of sequence influence per chromosome (see Materials and Methods for further details).*

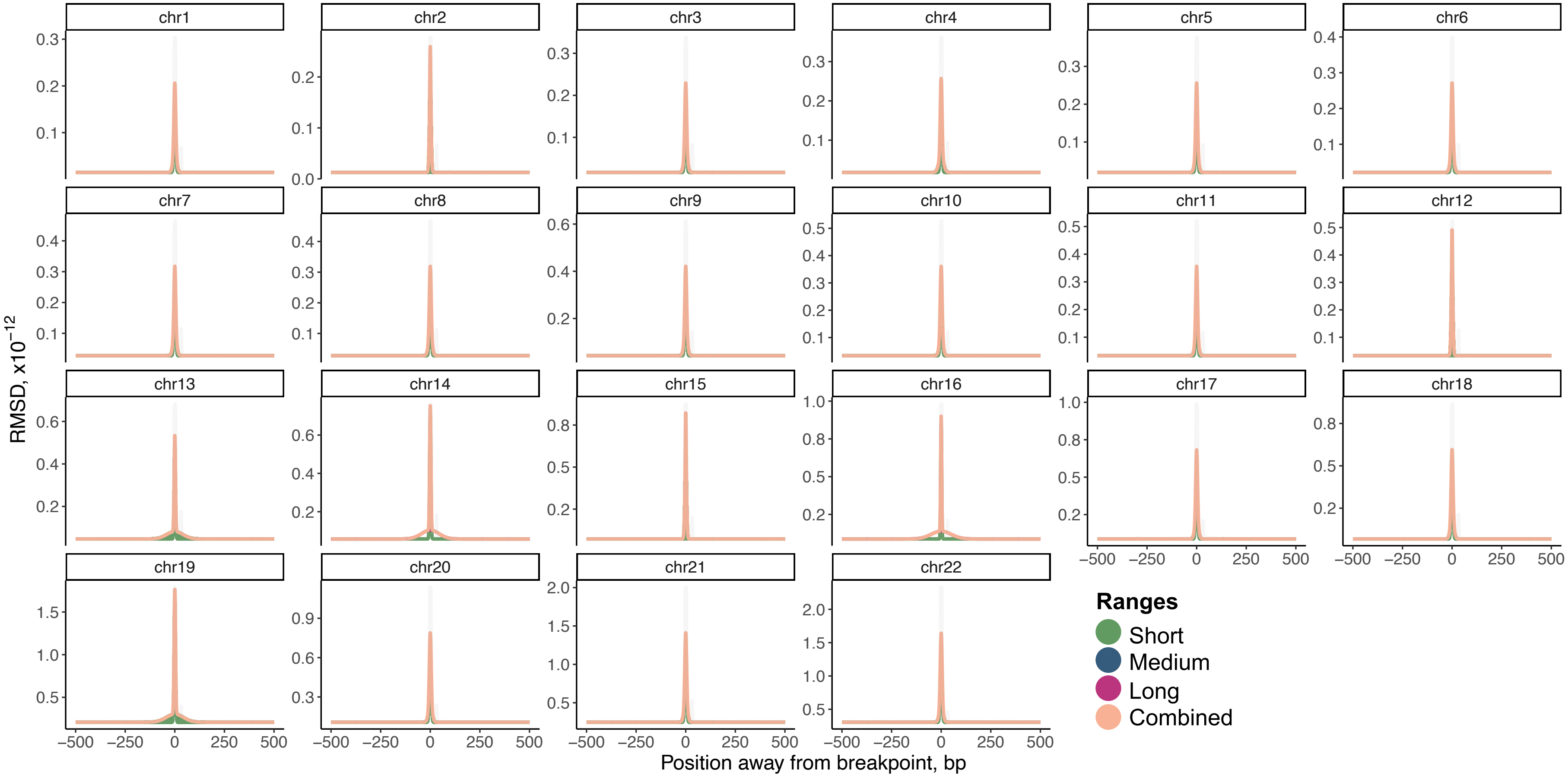

Figure S6. Quantifying the span of sequence-based influences for all autosomes of natural decay and fossilisation. *We selected one representative example dataset for the natural decay breakage class (Altai Neanderthal genome) [3], and calculated the range of sequence influence assessed from -500 to +500 bases relative to the origin of the breakpoint at the zeroth position. Gaussian curves were fitted to quantify the range of sequence influence per chromosome (see Materials and Methods for further details).*

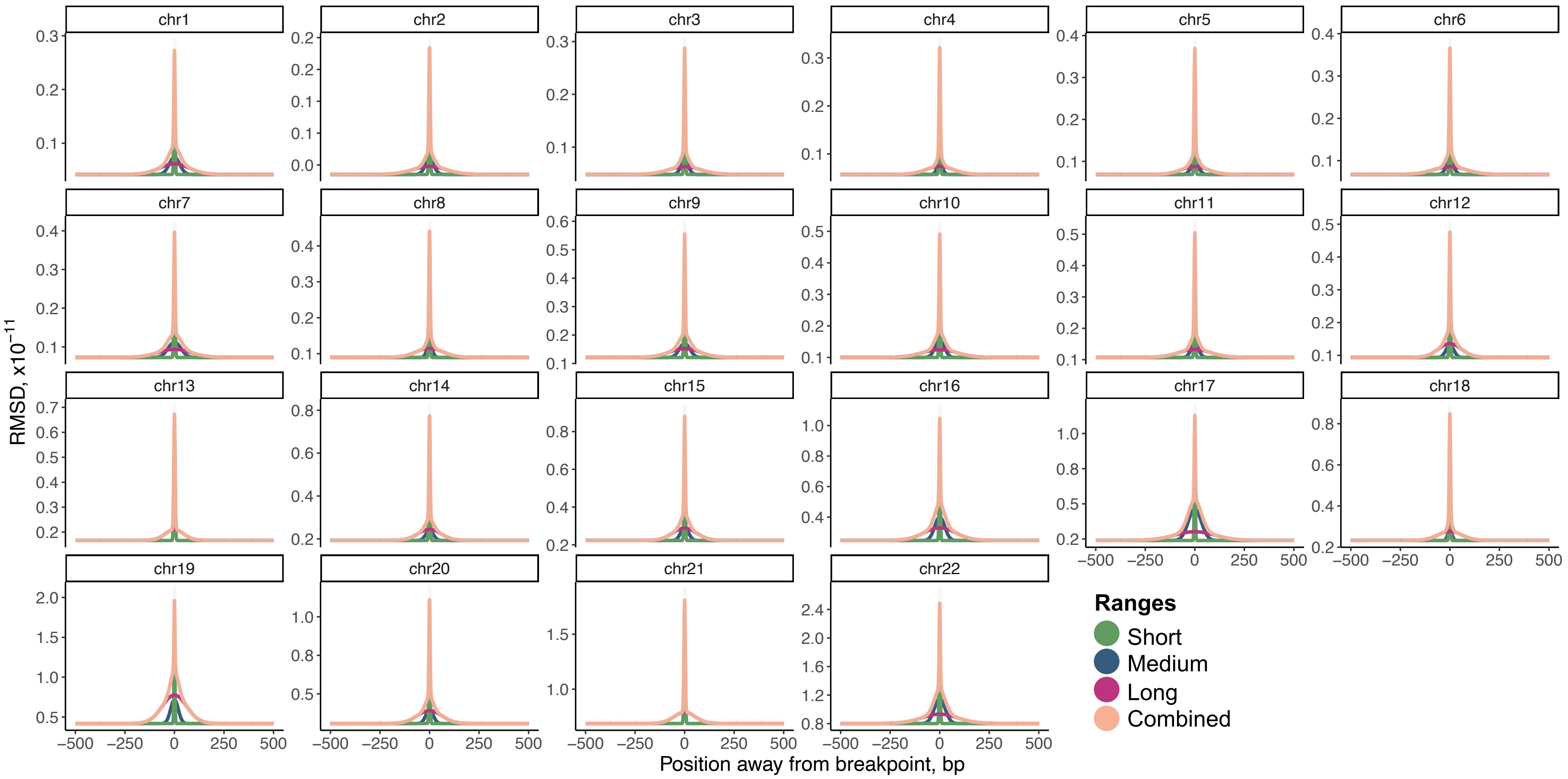

Figure S7. Quantifying the span of sequence-based influences for all autosomes of cell free DNA fragments. *We selected one representative example dataset for the cell free DNA breakage class (ovarian cancer) [2], and calculated the range of sequence influence assessed from -500 to +500 bases relative to the origin of the breakpoint at the zeroth position. Gaussian curves were fitted to quantify the range of sequence influence per chromosome (see Materials and Methods for further details).*

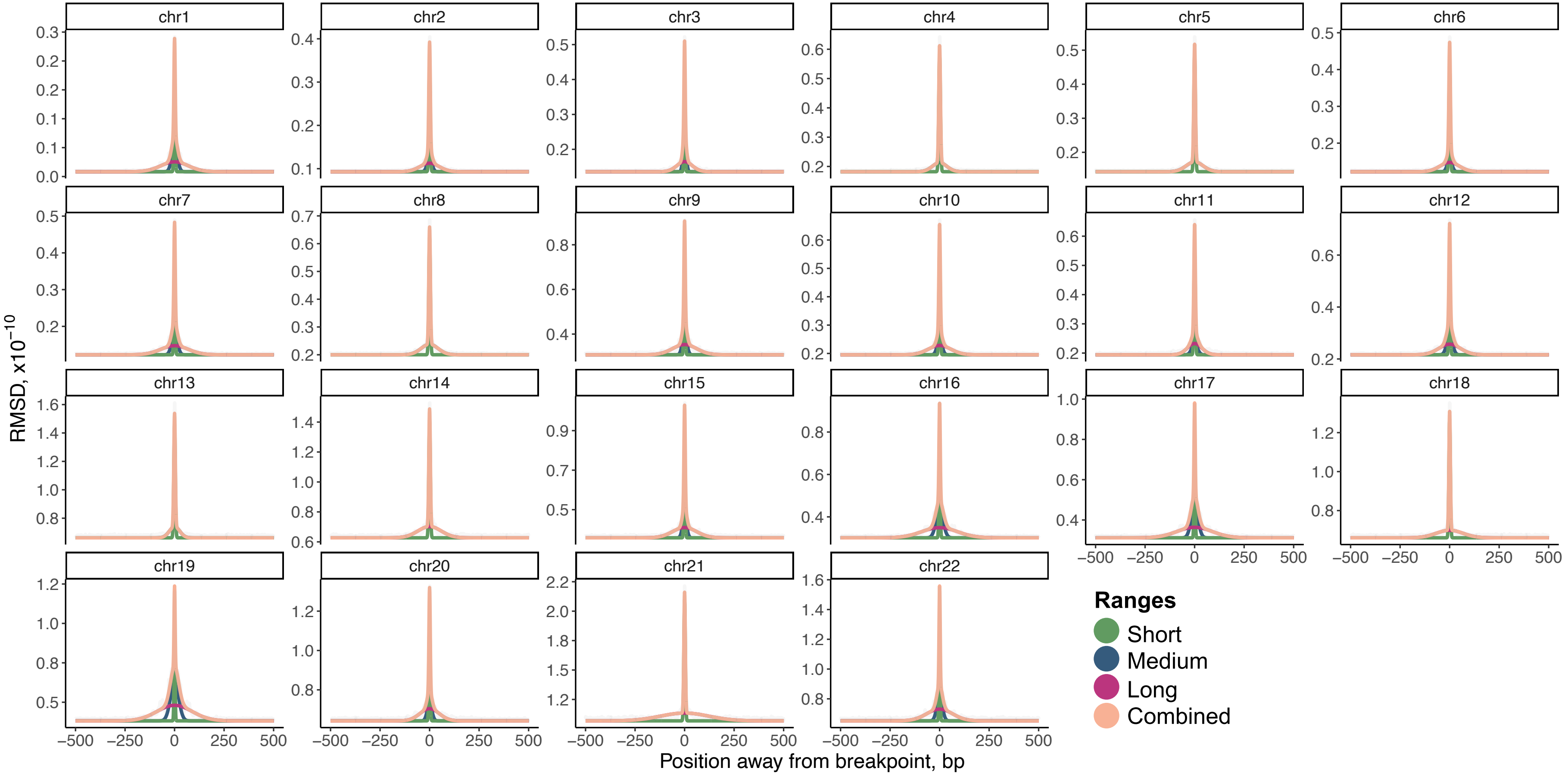

Figure S8. Quantifying the span of sequence-based influences for all autosomes of physiological and endogenous fragility. *We selected one representative example dataset for the physiological breaks (DNA fragility in K562 cells) [1], and calculated the range of sequence influence assessed from -500 to +500 bases relative to the origin of the breakpoint at the zeroth position. Gaussian curves were fitted to quantify the range of sequence influence per chromosome (see Materials and Methods for further details).*

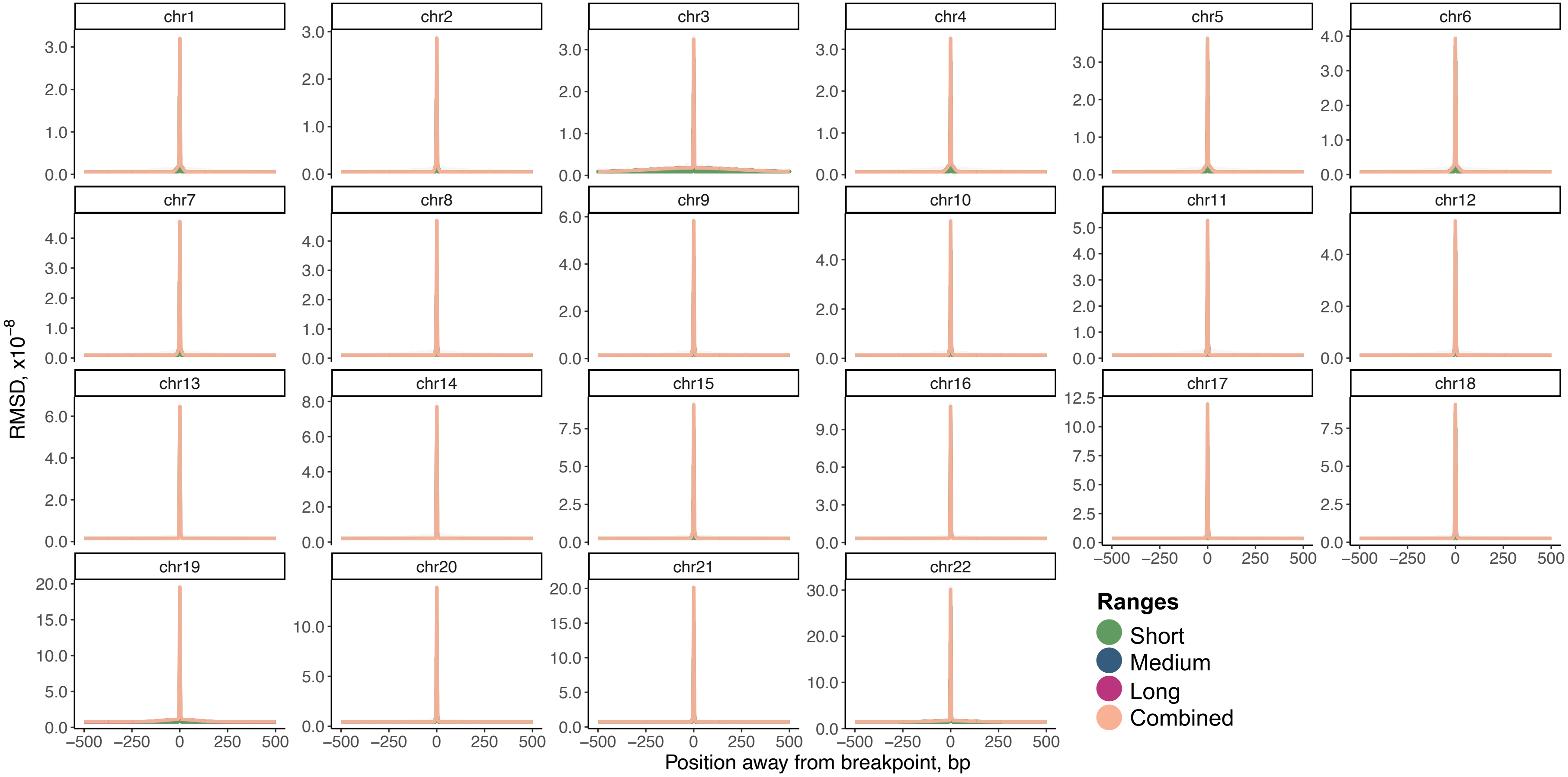

Figure S9. Quantifying the span of sequence-based influences for all autosomes of enzymatic cleavages. *We selected one representative example dataset for the enzymatic cleavage class (EcoRV restriction enzyme) [4], and calculated the range of sequence influence assessed from -500 to +500 bases relative to the origin of the breakpoint at the zeroth position. Gaussian curves were fitted to quantify the range of sequence influence per chromosome (see Materials and Methods for further details).*

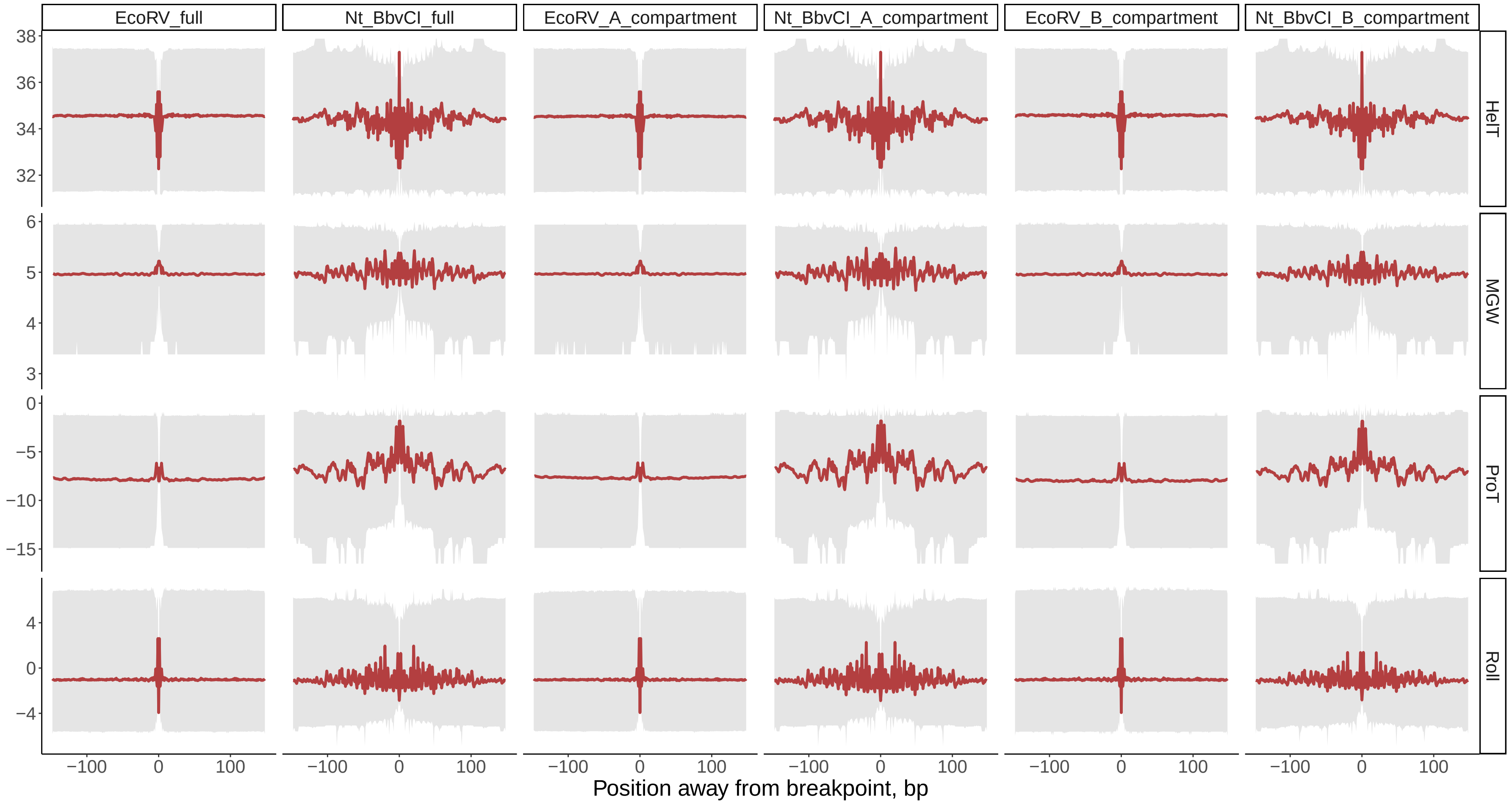

Figure S10. Comparing DNA shape predictions between EcoRV restriction enzyme [4] and Nt BbvCI endonuclease [6]. *The DNAShapeR library was used to predict four DNA shape parameters: the helical twist (HelT), minor groove width (MGW), propeller twist (ProT), and roll in a five-nucleotide sliding window in strides of one nucleotide. The general DNA shape characteristics of the Nt BbvCI endonuclease (**bottom**) appear to be highly oscillating throughout the medium range, and much more so compared to the EcoRV restriction enzyme (**top**), albeit the average behaviour is not too deviating from normal B-DNA conditions. The helical twist and propeller twist parameters seem to have the greatest rate of change around the breakpoint origin, which likely accommodates the binding of the enzyme's active site.*

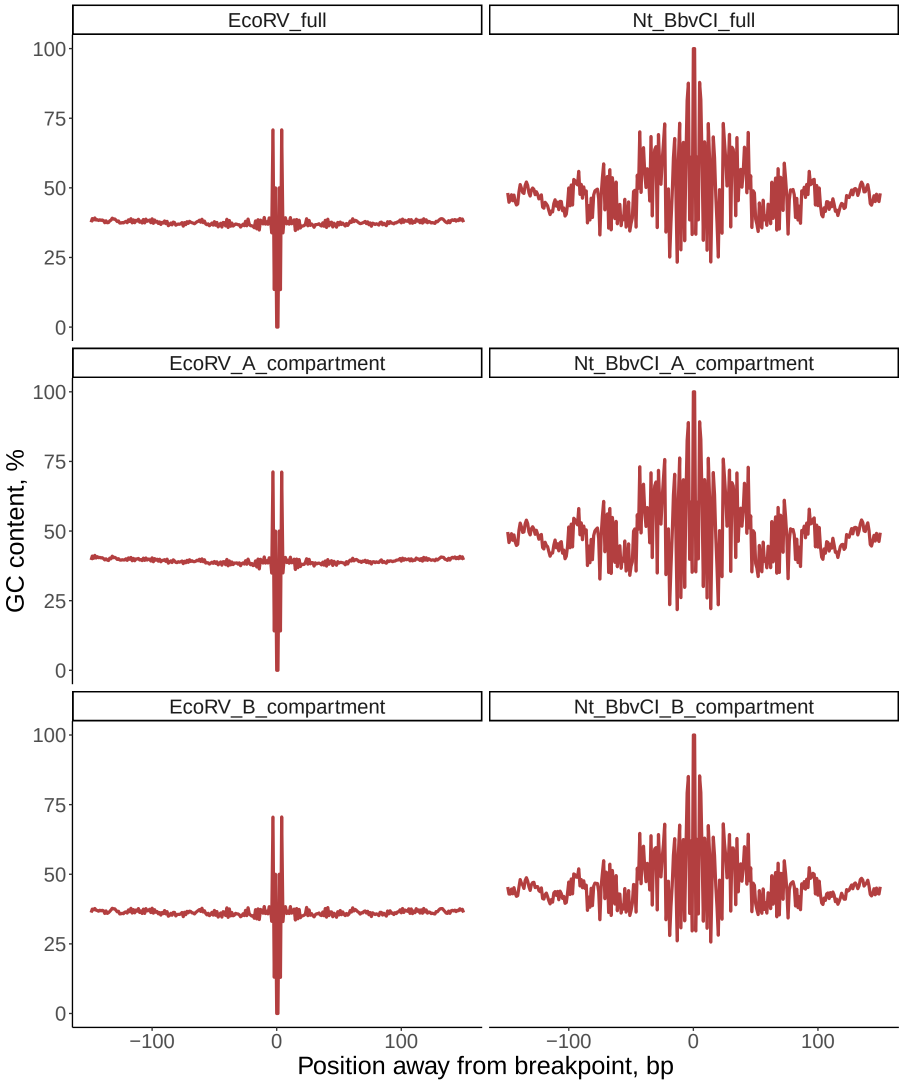

Figure S11. Comparing the GC content between EcoRV restriction enzyme [4] and Nt BbvCI endonuclease [6]. *The 1-base rolling window of the GC content displays greater oscillation within the medium-range effect and most significantly surrounding the location of the breakpoint. Similar to the observations in* ***Figure S10****, the GC content deviations are much greater in Nt BbvCI endonuclease (****bottom****) compared to the EcoRV restriction enzyme (****top****).*

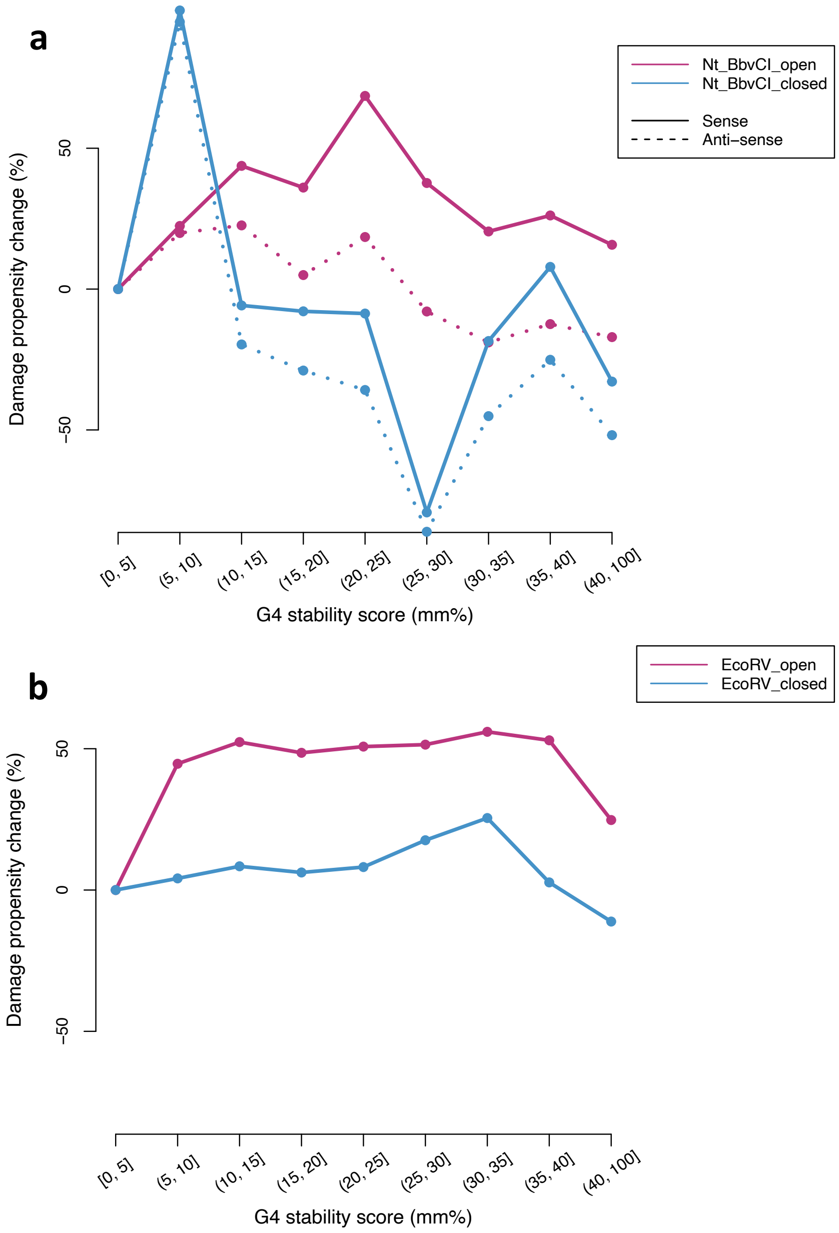

Figure S12. Comparing the structure-driven effects on genomic DNA breakage propensity at G-quadruplex sites between EcoRV restriction enzyme [4] and Nt BbvCI endonuclease [6]. *We studied the cleavage phenomenon within the A and B compartments separately (see Materials and Methods for further details). (**a**) G-quadruplex (G4) structures appear to have a de-protective effect on the strand they form, evidenced by the higher damage propensity (up to a +60% change) on the open/A compartment. This behaviour is similar, but more consistent for the EcoRV cleavage sites (**b**). The G4 structures exert a slight protective effect in the closed/B compartments in both enzyme cleavage sites. The damage propensity difference between sense and anti-sense strands (**a**) is likely due to the single-stranded breakage nature of the Nt BbvCI endonuclease.*

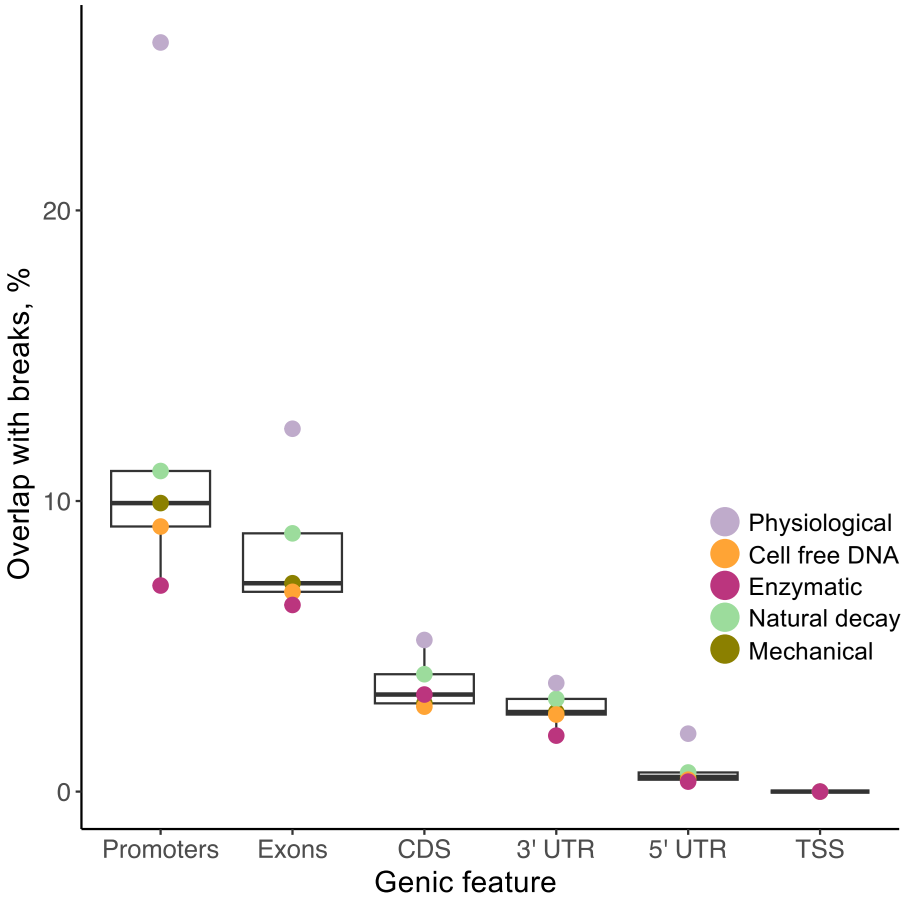

Figure S13. DNA fragility at sites excluding gene effects. *We selected one representative example dataset for each breakage class [1–5], and calculated the percent of overlaps of the exact breakpoint sites of an experiment within the full range of the given genic feature.*

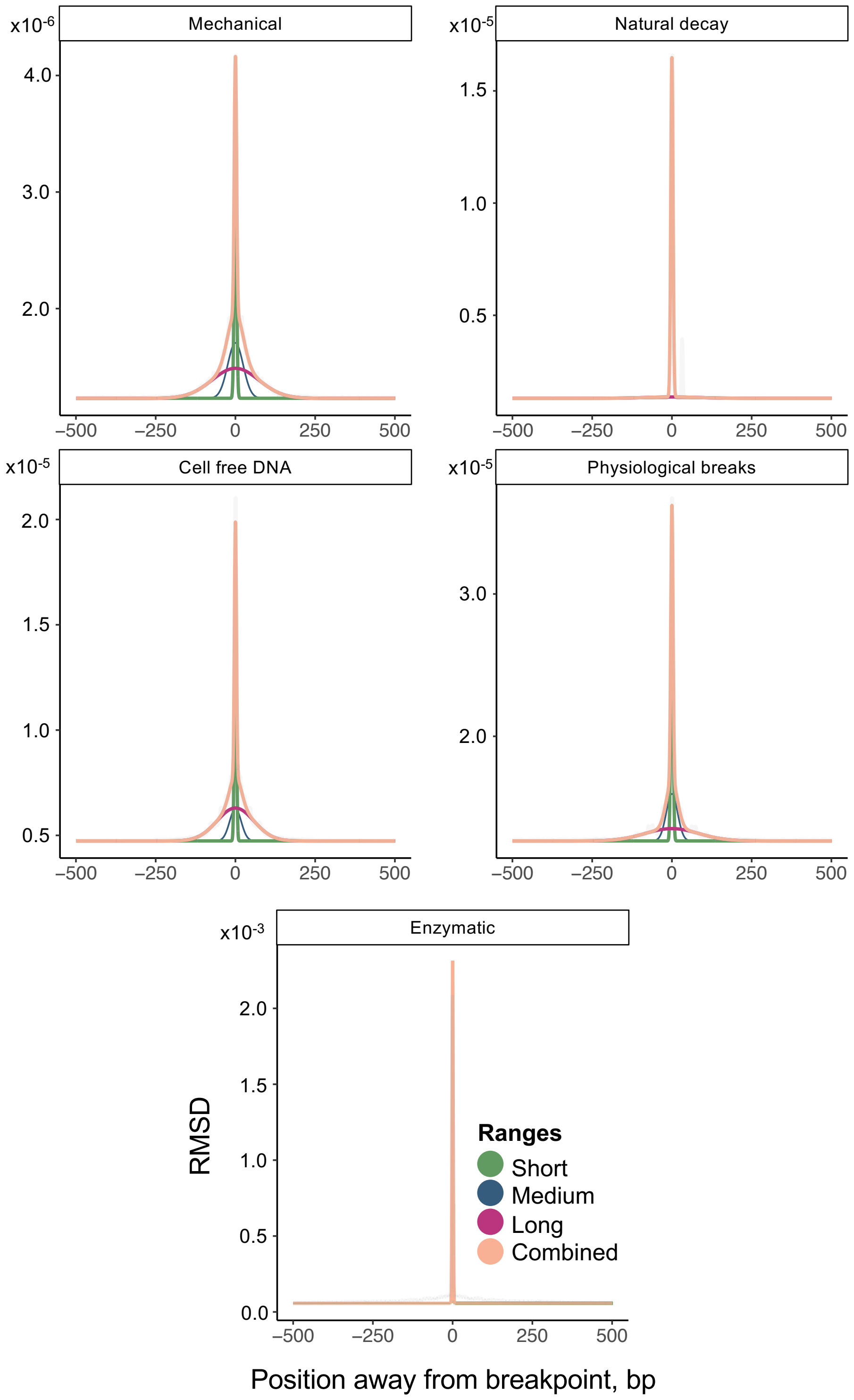

Figure S14. Quantifying the range effects excluding promoter regions. *We selected one representative example dataset for each breakage class [1–5], and removed the exact breakpoint sites of an experiment that fell within a promoter region. We calculated the span of intrinsic sequence influence within -500 to +500 positions of the breakpoint site located at the zeroth position and subsequently fitted Gaussian curves to the underlying RMSD values (see Materials and Methods for further details). Despite promoter regions exhibiting the highest percentage overlap with breakpoint sites, particularly under physiological and endogenous conditions, among the six genic features assessed (as shown in* ***Figure S13****), the removal of breakpoints occurring within these regions confirms the persistence of pronounced sequence-driven influences.*

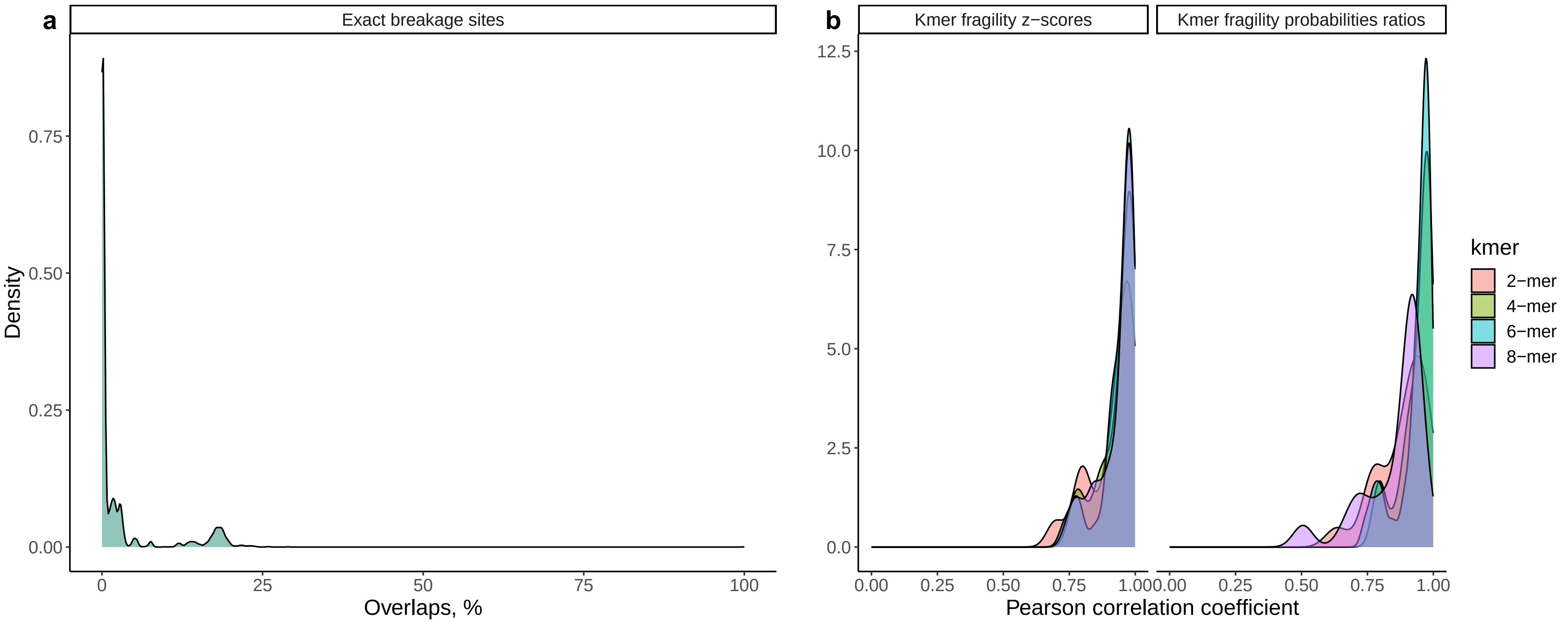

Figure S15. Quantifying the short-range intrinsic susceptibilities of DNA fragility. *We examined the percentage of exactly matched breakpoint sites shared across any replicas of the same experiment in which DNA strand breaks were induced by any of the breakage classes under study. While the highest percentage overlaps is nearly 30% between a 45k years-old Ust'-Ishim man and a ~50k years-old Neanderthal species found in the Altai mountains, the average across all of our collected and processed datasets is, on average, only 3% (**a**), suggesting that the breakage phenomenon follows a stochastic nature. (**b**) However, quantifying the k-meric enrichment through the probability ratios and intrinsic susceptibility z-score metrics, we find that the k-meric breakage propensities for all sizes k $\in$ {2,4,6,8} are highly correlated between replicas of the same experiment*

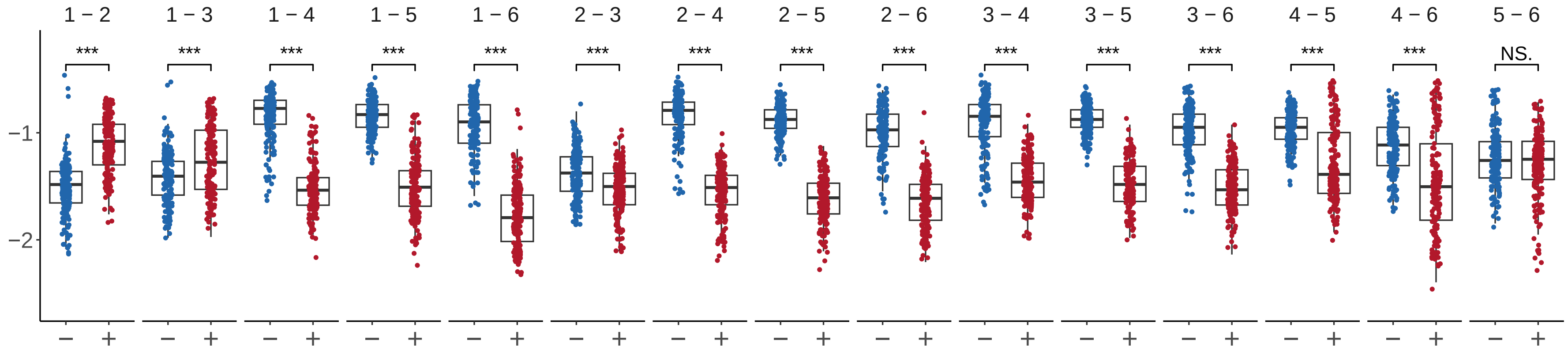

Figure S16. Comparing the hybridisation free energies in the B-DNA conformation. *We focused on the top 0.5% most fragile and top 0.5% most resistant k-meric breakage propensities between any two clusters from (**Figure 2**). We compared the hybridisation free energies in the B-DNA conformation between these two clusters to reveal any aggregate characteristics of the DNA structure at the breakpoint site. The result is visualised as a boxplot. There are 15 unique cluster comparisons where the plot title indicates the direction of comparison. For example, the left-most result shows cluster one minus cluster two. Here, the left boxplot (blue scatter points, -) represents highly resistant k-meric breakage propensities, while the right boxplot (red scatter points, +) represents highly fragile k-meric breakage propensities. A two-sample t-test was performed between the k-meric populations, revealing the statistical significance over the boxplot (******* $p < 0.001$, **NS.** not significant).*

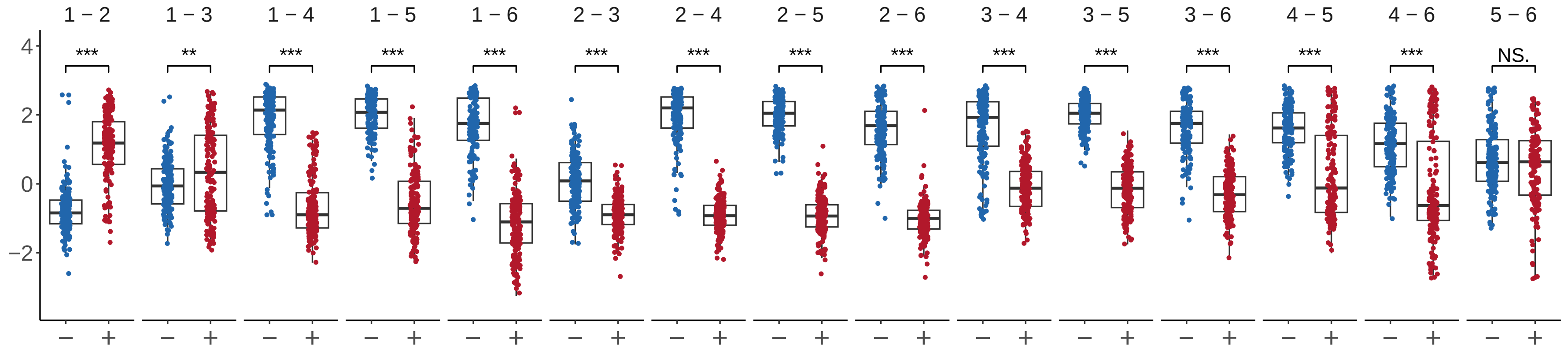

Figure S17. Comparing the delta heat of formation in the B-DNA conformation. *We focused on the top 0.5% most fragile and top 0.5% most resistant k-meric breakage propensities between any two clusters from (**Figure 2**). We compared the delta heat of formation in the B-DNA conformation between these two clusters to reveal any aggregate characteristics of the DNA structure at the breakpoint site. The result is visualised as a boxplot. There are 15 unique cluster comparisons where the plot title indicates the direction of comparison. For example, the left-most result shows cluster one minus cluster two. Here, the left boxplot (blue scatter points, -) represents highly resistant k-meric breakage propensities, while the right boxplot (red scatter points, +) represents highly fragile k-meric breakage propensities. A two-sample t-test was performed between the k-meric populations, revealing the statistical significance over the boxplot (**** $p < 0.001$, ***NS.*** *not significant).*

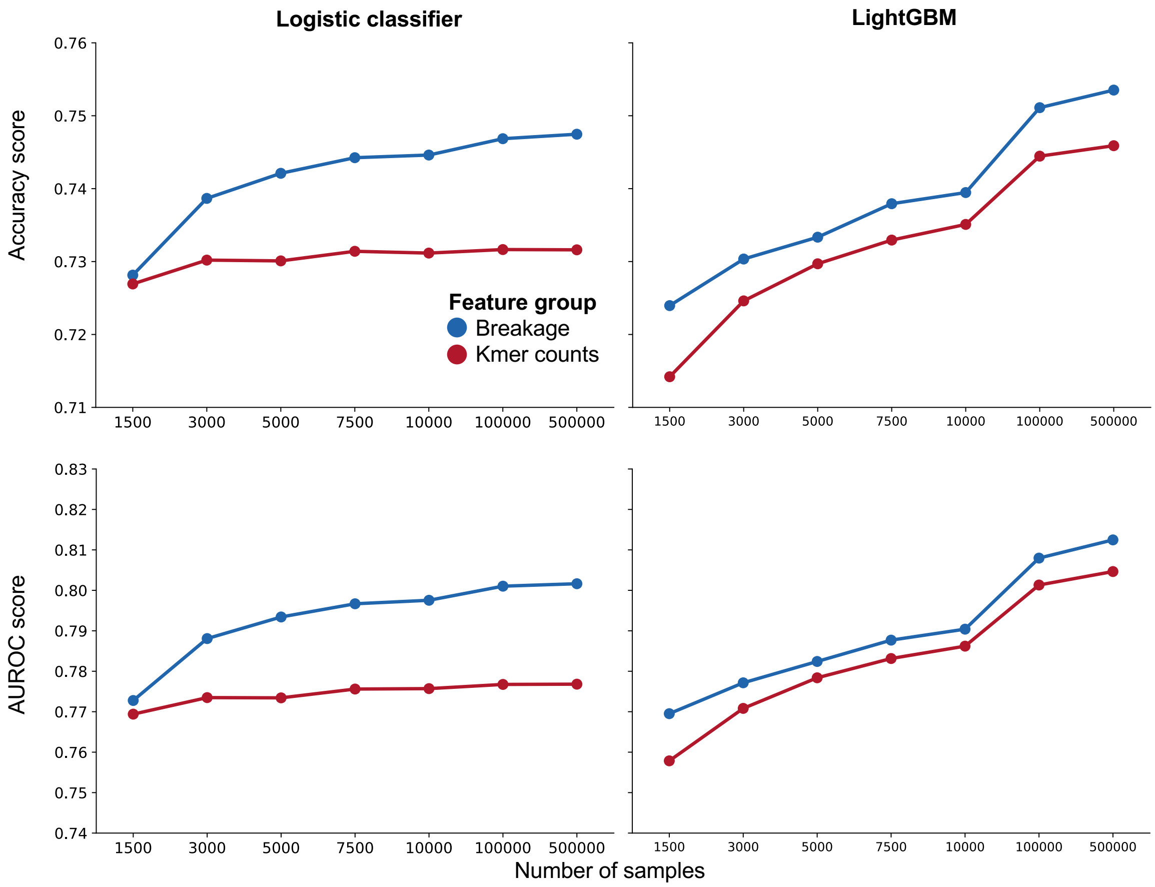

Figure S18. Comparing the predictive power from k-meric breakage susceptibility scores with k-mer counting metrics as feature groups. *The full dataset is first split into 70% training and 30% testing sets. The training set was randomly downsampled to only contain 1.5k, 3k, 5k, 7.5k, 10k, 100k, and 500k samples. In each subset of the training data, we trained a logistic classifier on only the k-meric breakage susceptibility scores and separately only the triplet k-meric counts, and repeated this for the lightGBM model. The models are applied to the 30% testing data, where we recorded the area under the receiver operating characteristic (AUROC) curve and the accuracy metrics. The results show that our k-meric breakage susceptibility scores consistently deliver enhanced predictive power compared to simple counting metrics.*

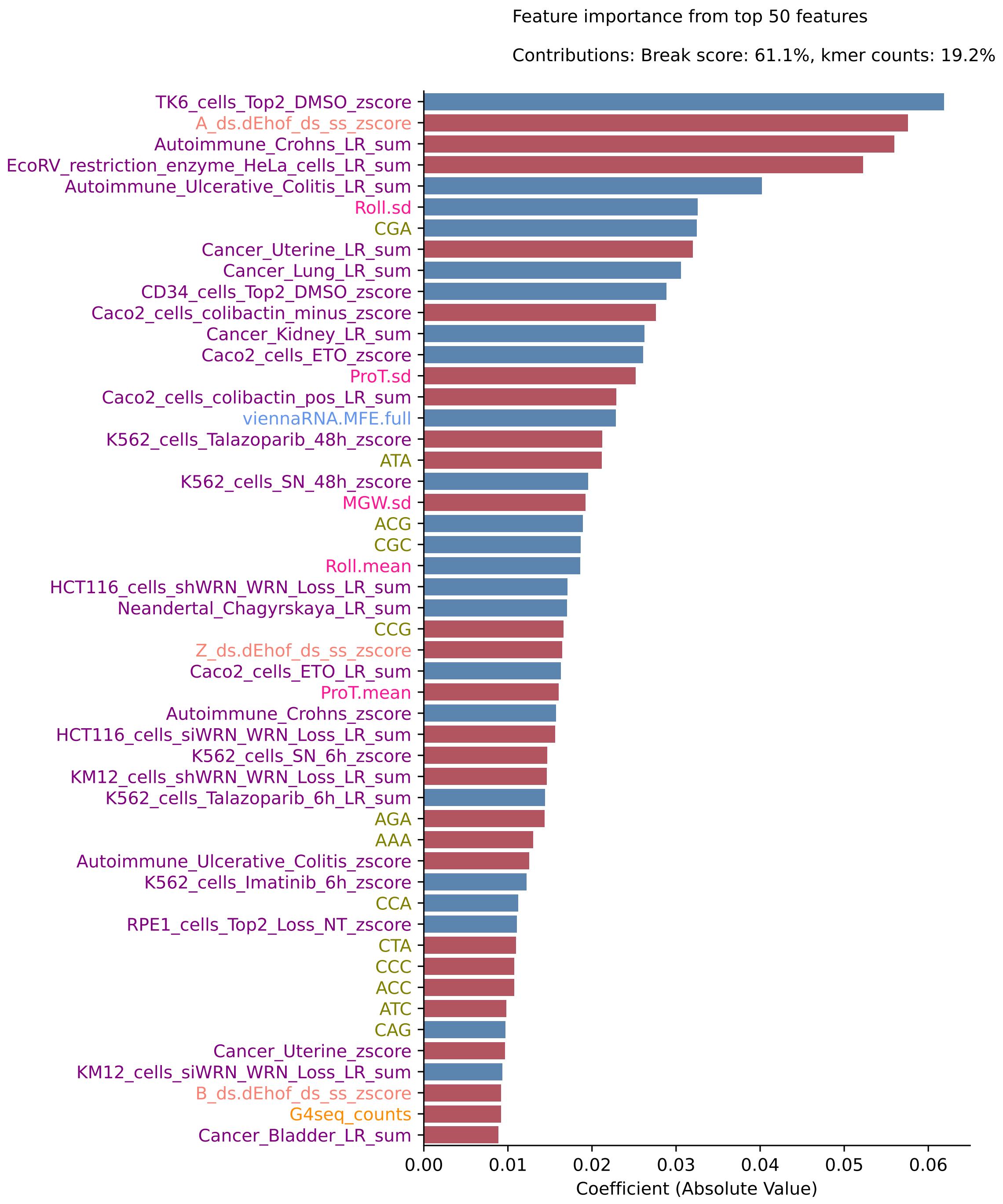

Figure S19. Top 50 features by coefficient magnitude from the logistic classification model. *The absolute coefficient of each feature was taken as a proxy for its importance in driving the model's predictive performance, and sorted from highest to lowest. We present the top 50 features as a bar plot, where each feature's line segment is coloured based on the sign of the coefficient as blue (positive) or red (negative). Each feature on the y-axis is coloured based on the feature group. Here, the features with the highest coefficients, and thus, higher relative importance, are a mix of k-meric breakage susceptibility scores, DNA shape parameters, quantum mechanical parameters, and some triplet counts.*

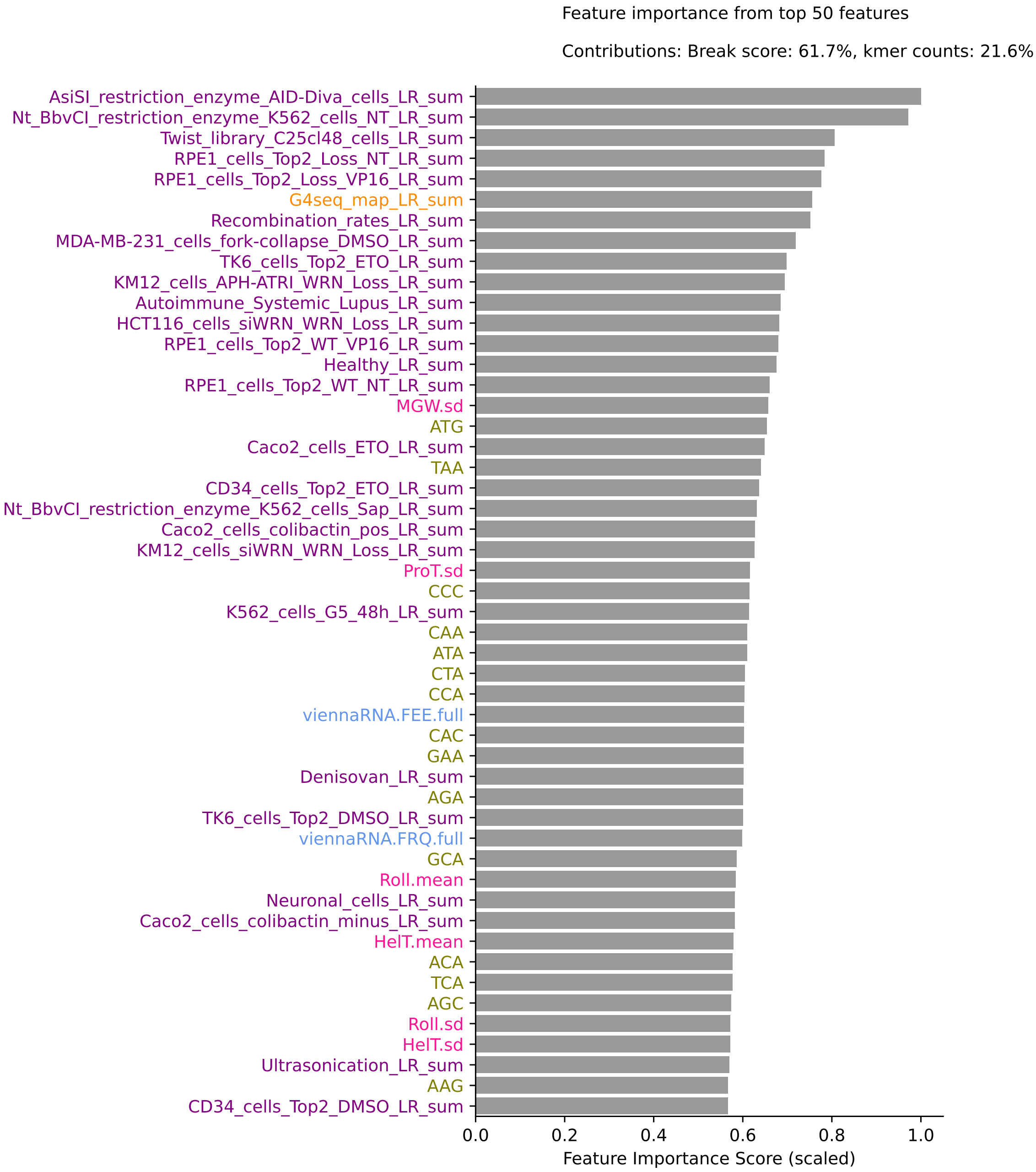

Figure S20. Top 50 features by coefficient magnitude from the tree-based, light gradient boosting machine learning model. *The absolute coefficient of each feature was taken as a proxy for its importance in driving the model's predictive performance, and sorted from highest to lowest. We present the top 50 features as a bar plot. The k-meric scores form the majority of influential features, while triplet counts had a lesser contribution.*

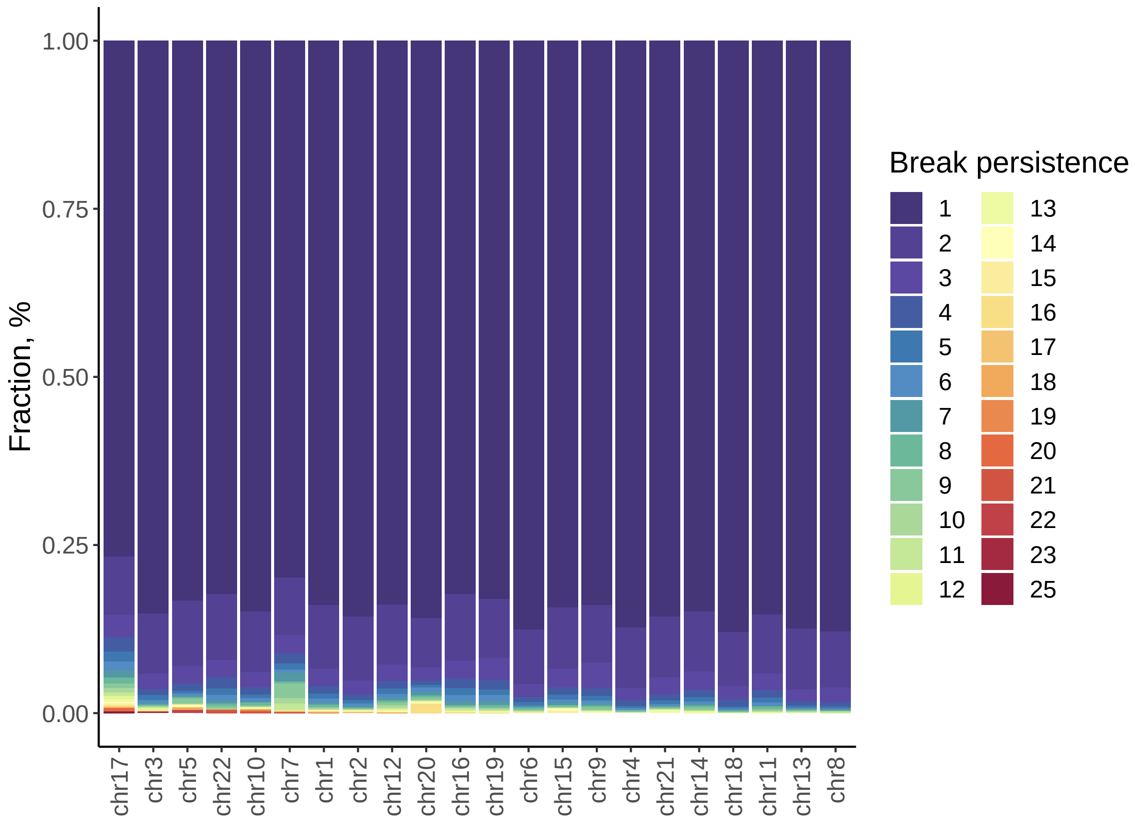

Figure S21. Fraction of cancer-associated break persistence for each autosome. *We took the COSMIC database-reported cancer-associated DNA strand breaks across deletions, insertions, indels (insertions and deletions), duplications, inversions, and both inter- and intra-chromosomal translocations. As a given chromosomal position may break across multiple unique tissue and cancer (TC) combinations, we counted the number of TC types that shared the identical broken chromosomal position. We present the fraction of break persistence for each autosome.*

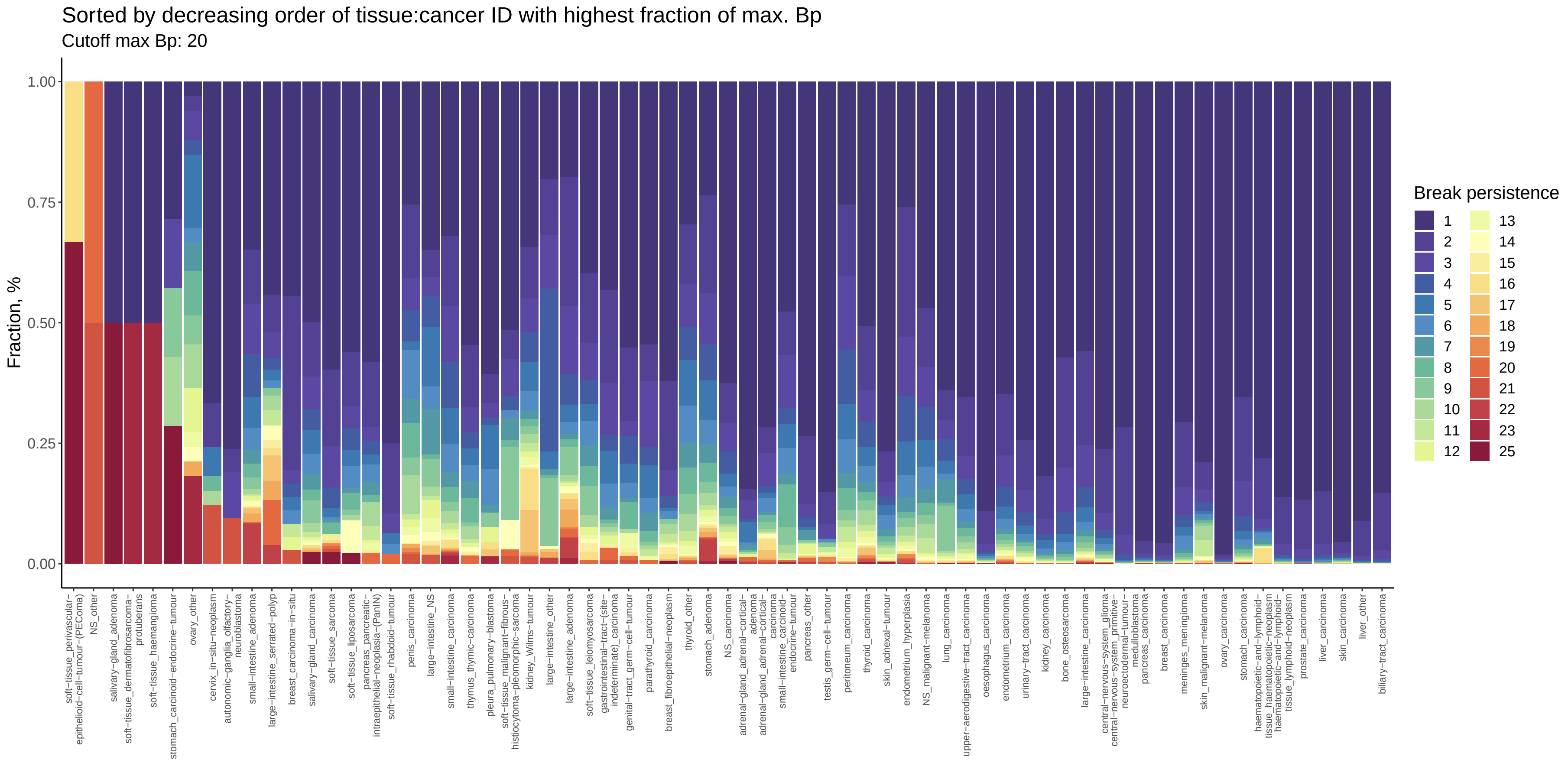

Figure S22. Fraction of cancer-associated break persistence of the highest occurring unique tissue and cancer combinations. *Zooming into the top 19 unique tissue and cancer (TC) types that make up 95% of all unique TC combinations, we next explored the contribution of each persistent break by TC type. Our results show that various cancers in soft tissue have a high frequency of persistent breaks, with perivascular epithelioid cell tumours, dermatofibroma sarcoma protuberances, and haemangioma having at least 50% of their DNA strand breaks occurring at chromosomal positions that are also shared with 22 or more other unique TC types.*

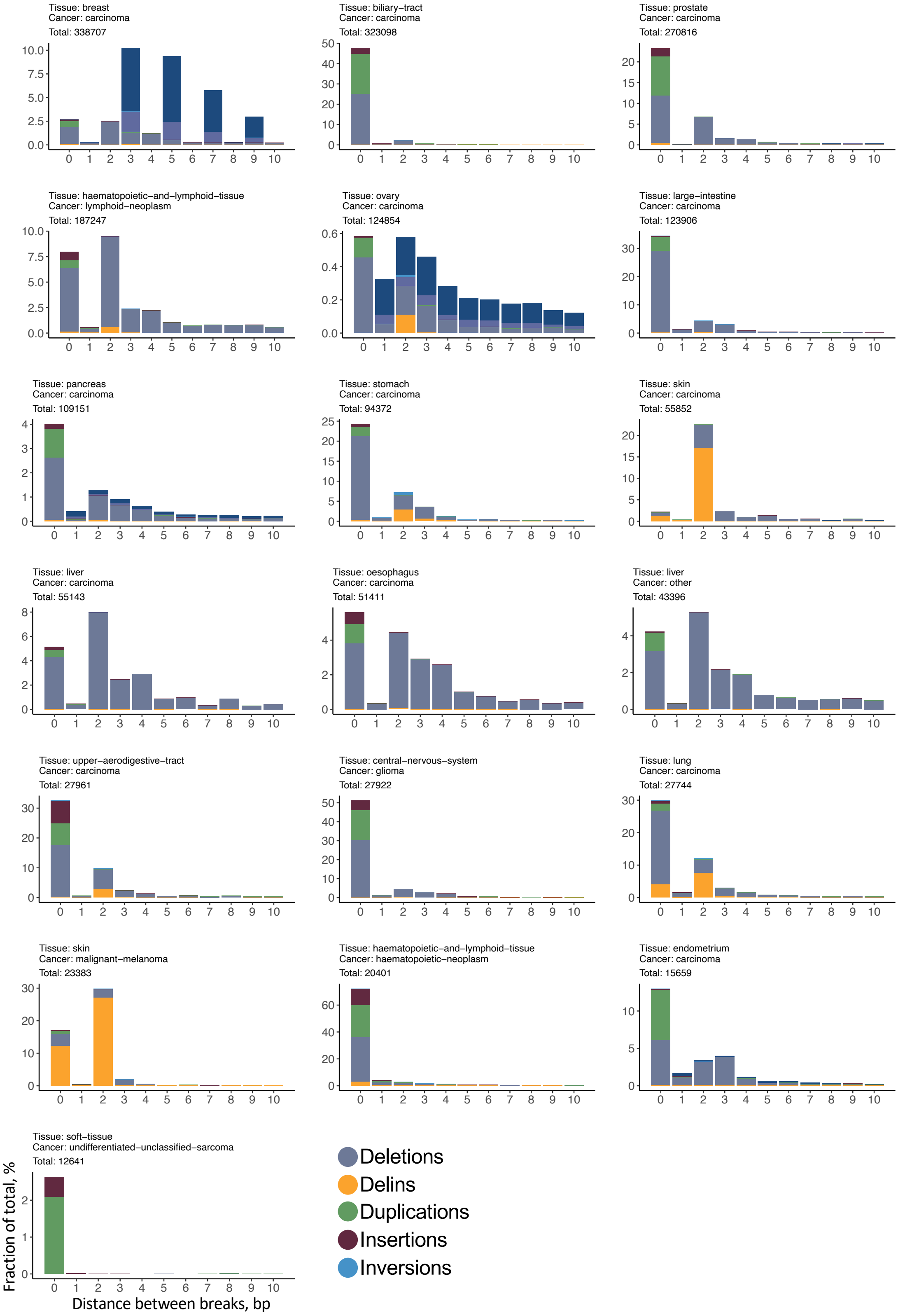

Figure S23. Distance between the DNA breakpoint position for each unique tissue and cancer combination by breakage type. *Zooming into the top 19 unique tissue and cancer (TC) types, we examined the number of bases between each type of break, separately for each TC type. The majority of the breaks are located in close proximity, with an average of 80% of strand break types occurring within 10 base pairs apart.*

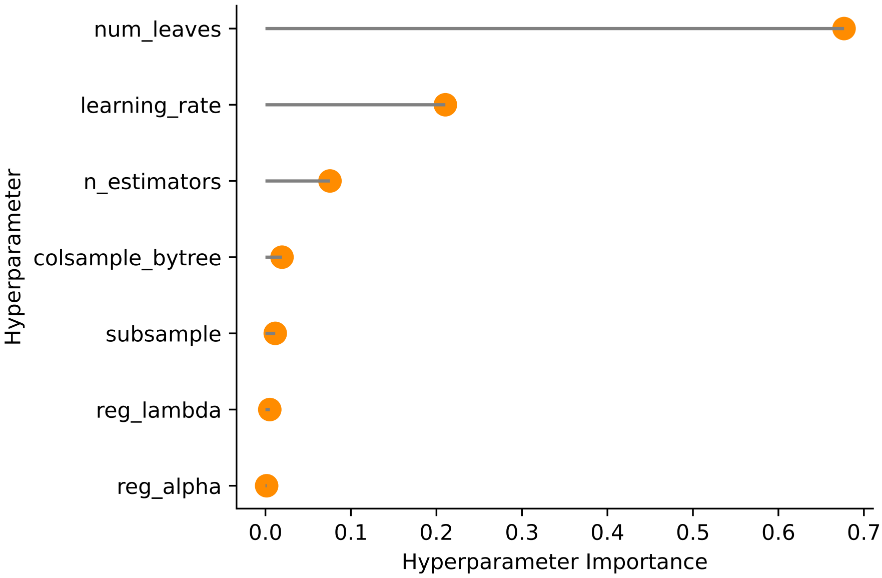

Figure S24. The LightGBM hyperparameter importance based on completed trials in the Optuna study*. The most powerful hyperparameters to influence the nature of the LightGBB model tuning process are `number of leaves`, and the `learning rate`, followed by the `number of estimators`. The `number of leaves` sets the model's complexity and allows the model to learn more granular patterns with more complex decisions but it comes at the cost of higher computational requirements and the risk of over-fitting. Meanwhile, the `learning rate` determines the speed and granularity with which the model learns. The effectiveness of the `number of estimators` is contingent upon the `learning rate`; they reciprocally influence each other.*

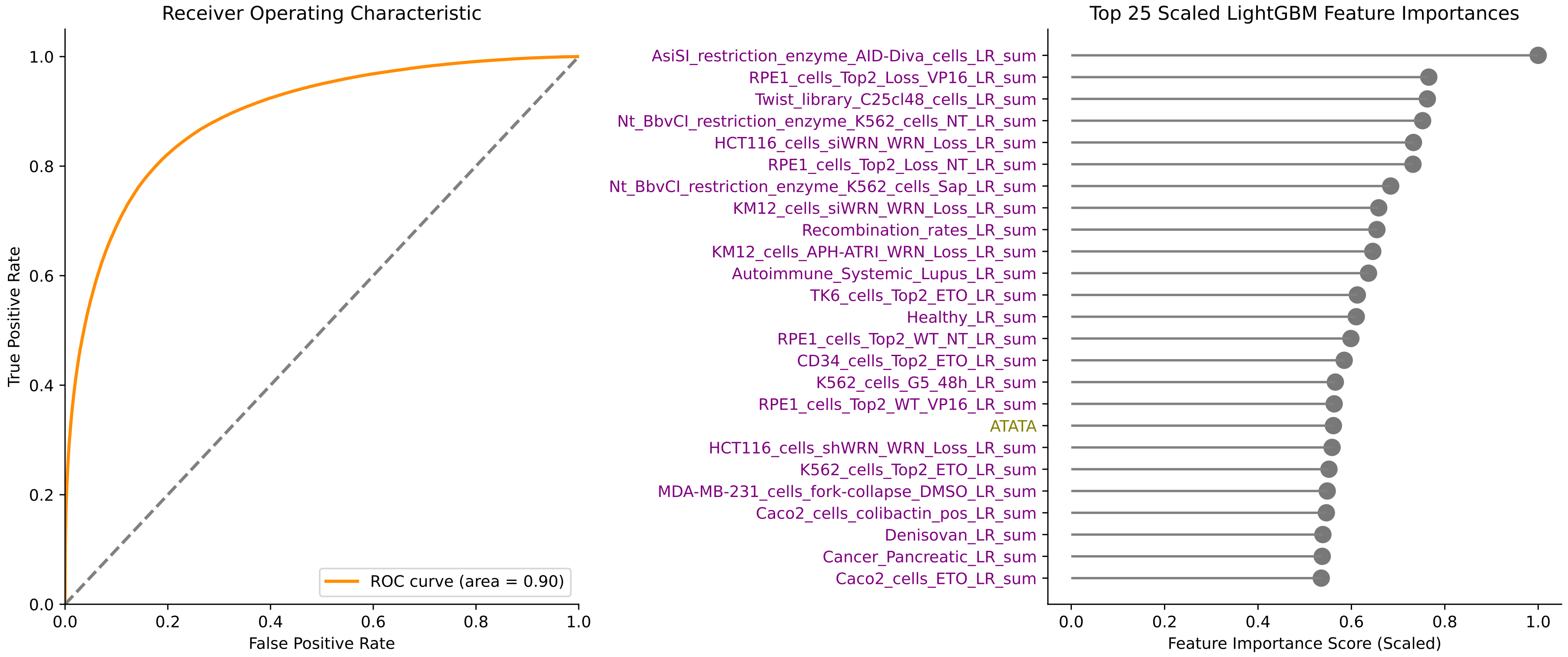

Figure S25. Generalised DNA fragility model performance. *(**a**) Area under the receiver operating characteristic (AUROC) curve showing the performance of the LightGBM classifier for the presence or absence of a DNA strand break. (**b**) The top 25 features ranked by relative importance for the achieved prediction quality. The importance of each feature is normalised with respect to the highest-ranked feature (GC skew).*

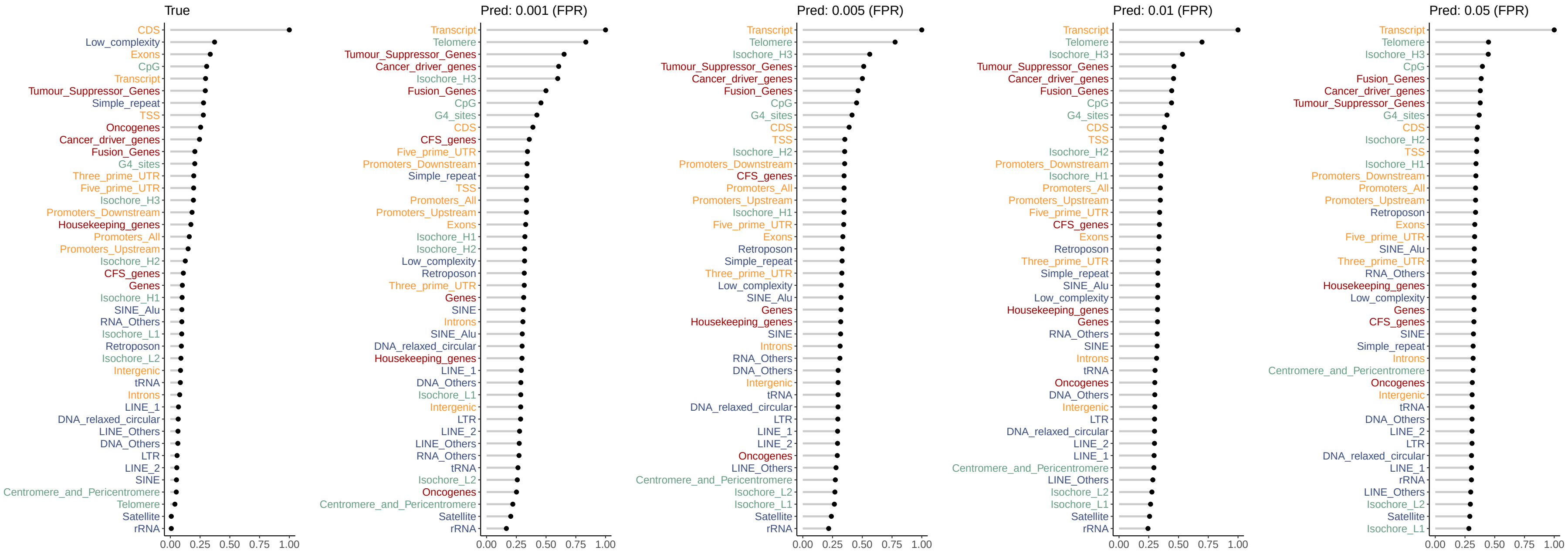

Figure S26. Predicted and COSMIC DNA strand break positions coinciding with annotated genes and other genomic features associated with the T2T human genome assembly. *The y-axis represents the relative fragility, calculated as the total number of COSMIC or predicted DNA strand breaks coinciding with the region of the given feature normalised by the total length of that feature. All lollipop plots show the relative fragility normalised for the most fragile feature to have a value of one. Hence, all the remaining features represent the fractions of the fragility from the most fragile feature. The left-most plot is associated with COSMIC DNA strand breaks, whereas the remaining three plots are associated with DNA strand breaks predicted by the LightGBM model, ordered by the increasing rate of false positives, illustrated at the top of each plot. We specifically highlight the Kolobok, Crypton, and Merlin DNA repeat families because these subclasses of DNA repeats only appear 410, 307, and 246 times, respectively, in the table, far less than other subclasses, including LINE1 and LINE2 elements.*

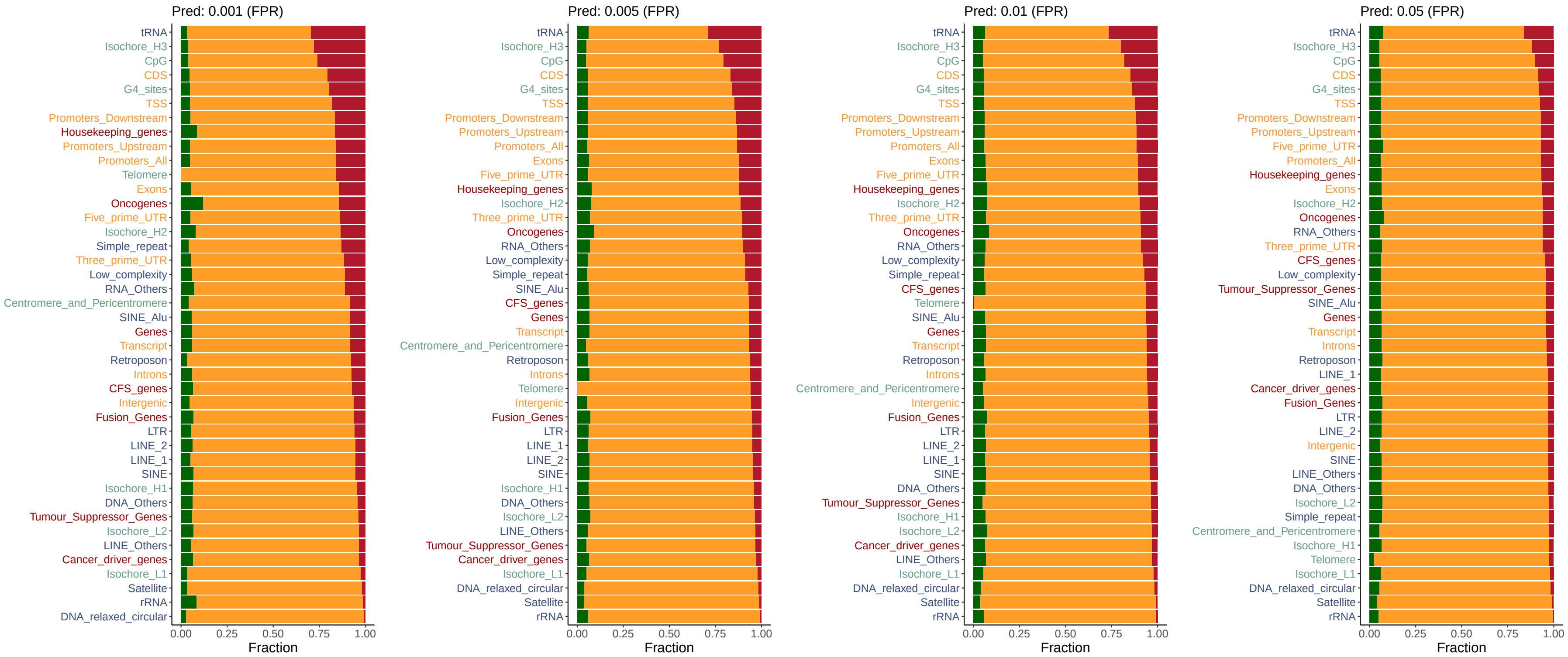

Figure S27. Predicted DNA fragility classified into low, medium, and high fragility coinciding with annotated genes and other genomic features associated with the T2T human genome assembly. *The y-axis represents the relative fragility, calculated as the total number of predicted DNA strand breaks coinciding with the region of the given feature normalised by the total length of that feature. The stacked barplot shows the percentage of each of the low (green), medium (orange), and high (red) fragility. The genomic features on the y-axis are ordered from the highest to lowest percentage share of highly fragile sites compared to the remaining low and medium fragile sites. The four plots are associated with DNA strand breaks predicted by the LightGBM model, ordered by the increasing rate of false positives, illustrated at the top of each plot.*

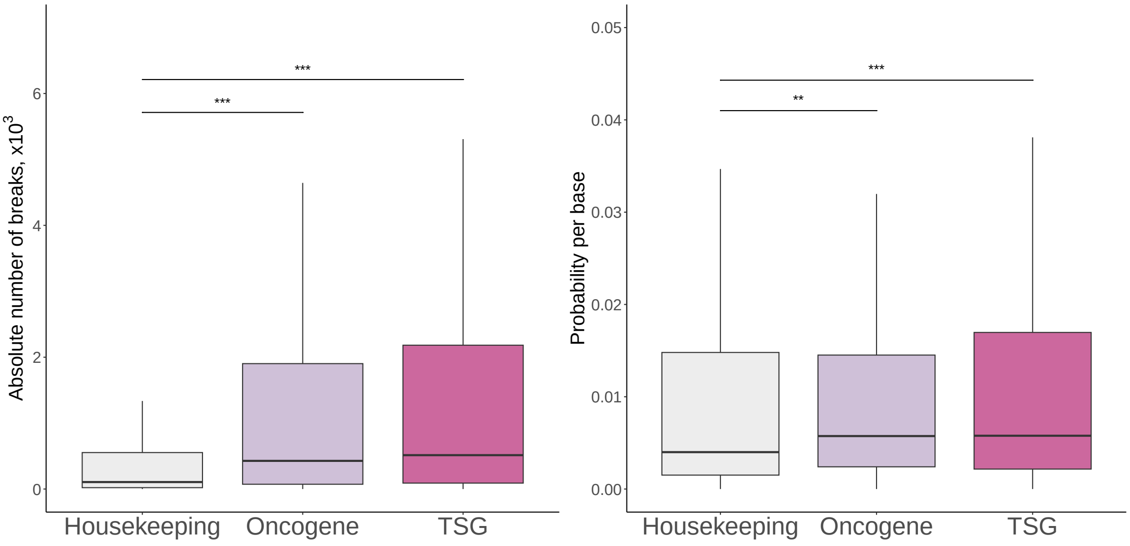

Figure S28. Predicted DNA fragility coinciding within genes related to housekeeping, oncogenes, and tumour suppressor genes. *(**left**) The y-axis represents the absolute number of breaks calculated as the total number of predicted DNA strand breaks coinciding with the region of the given gene. (**right**) The relative fragility on the y-axis is calculated in the same process as the **left** plot but additionally normalises the value by the total length of this gene. The results are presented in a boxplot with the statistical significance annotated at the top (**** $p < 0.001$*, *** $p < 0.05$*). Both plots only show the model's predictions using the most conservative threshold.*

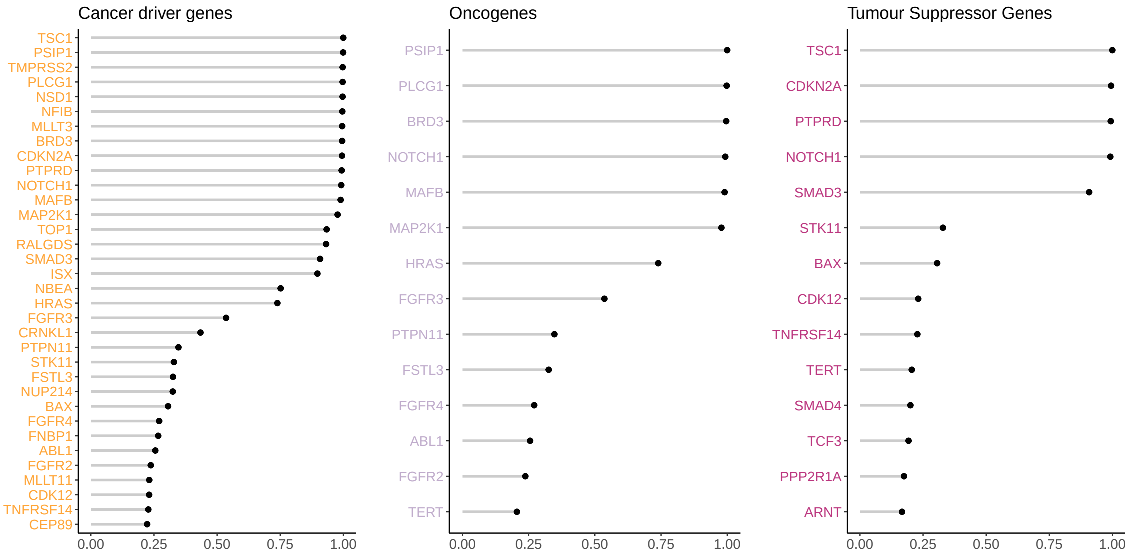

Figure S29. Predicted DNA fragility coinciding within genes related to cancer driver genes, oncogenes, and tumour suppressor genes. *The x-axis represents the relative fragility which is calculated as the total number of predicted DNA strand breaks coinciding with the region of the given given normalised by the total length of this gene. The relative fragility is then normalised by the most fragile gene on the list and ranked. The plot only shows the model's predictions using the most conservative threshold.*

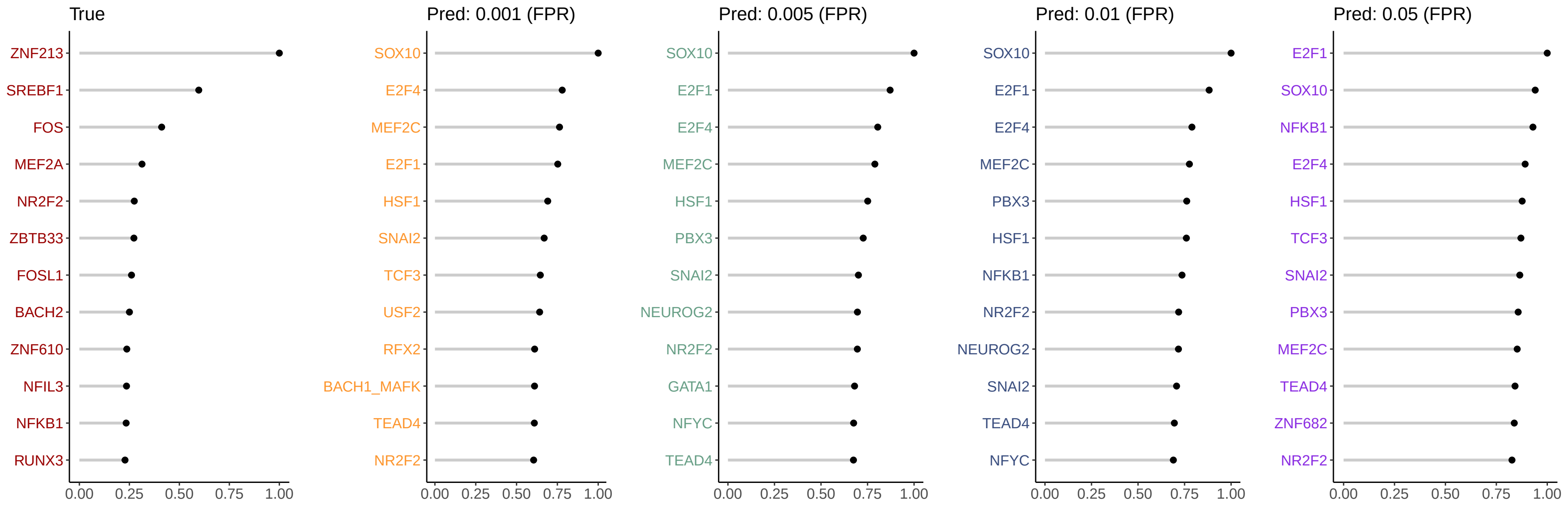

Figure S30. Predicted and COSMIC DNA strand breaks coinciding with 247 transcription factors from the JASPAR 2024 database. *The x-axis represents the relative fragility, calculated as the total number of COSMIC or predicted DNA strand breaks coinciding with the region of the given transcription factor (TF) normalised by the total length of that TF across all binding sites. All lollipop plots show the relative fragility normalised for the most fragile TF genes to have a value of one. Hence, all the remaining TFs represent the fractions of the fragility from the most fragile TF genes. The left-most plot is associated with COSMIC DNA strand breaks, whereas the remaining three plots are associated with DNA strand breaks predicted by the LightGBM model, ordered by the increasing rate of false positives, illustrated at the top of each plot.*

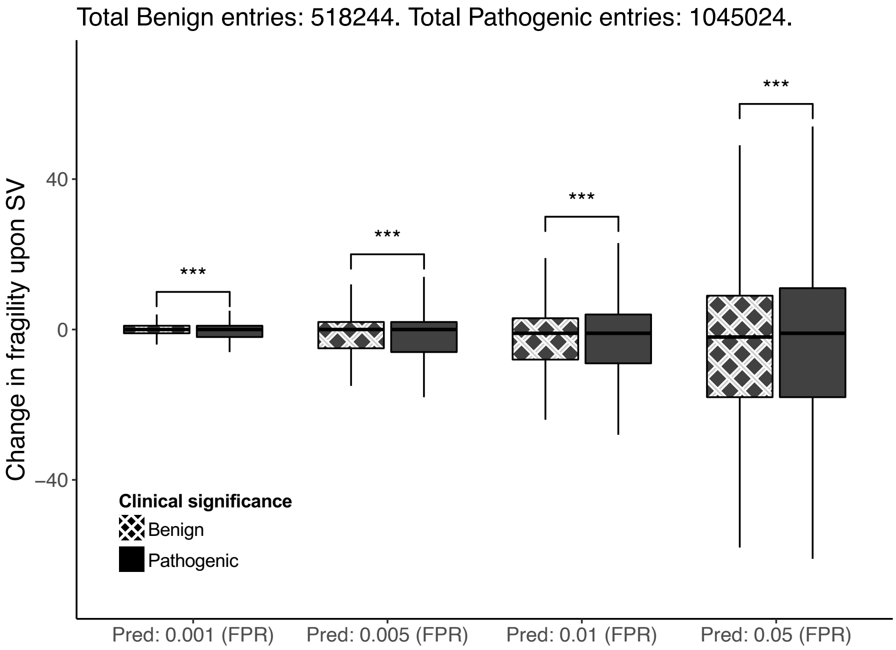

Figure S31. Predicted DNA strand breaks at clinically significant structural variant (SV) sites. *The y-axis represents the relative delta fragility, calculated as the difference between the total number of predicted DNA strand breaks of the sequence after and before the sequence variant and normalised by the length of the sequence. The results are aggregated into a boxplot for each of the four different thresholds applied to our model's predictions on the x-axis. As such, the y-axis shows the total number of DNA breakpoints "before" and "after" the SV occurrence within this fixed context range. We performed a two-sample t-test between the benign and pathogenic normalised delta fragilities, separately for each of the four model's thresholds, revealing the statistical significance over the boxplot (**** $p < 0.001$.*)*

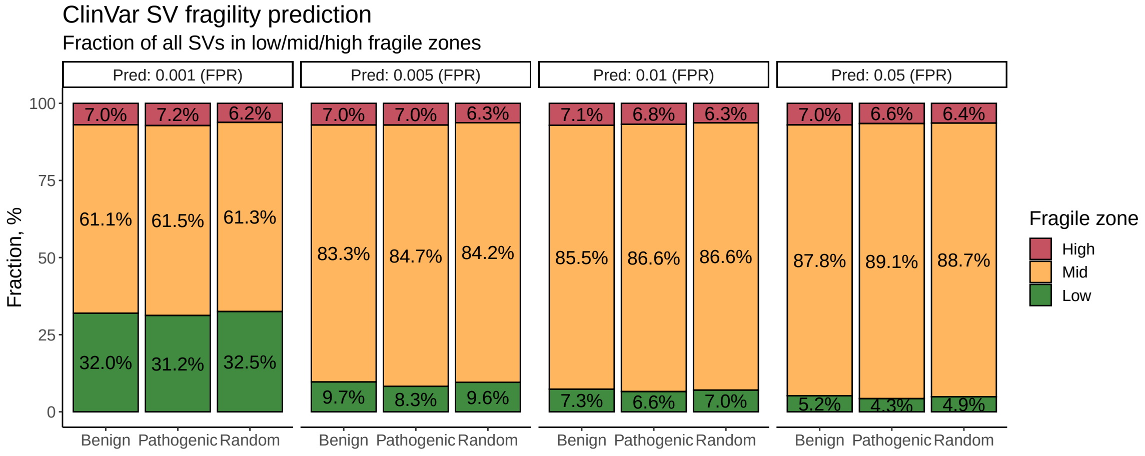

Figure S32. Fraction of all structural variants coinciding within low, medium, and highly fragile regions of the human genome. *We binned the entire human genome into non-overlapping 1,960 bases-long regions (see Materials and Methods for further details). In each of these bins, we counted the total number of predicted DNA strand breaks and classified the bins into three fragility levels: the bottom and top 5% of the distribution of breaks as "low" and "high" fragility, respectively, as an indicator of cold and hot break zones, and the remainder as "medium" fragility. We calculated the proportion of all clinically significant structural variants (SVs) coinciding with each of these three regions illustrated on the y-axis as a fraction. The results are presented as a stacked barplot separately for benign and pathogenic SVs for each of the four different thresholds applied to our model's predictions on the x-axis.*

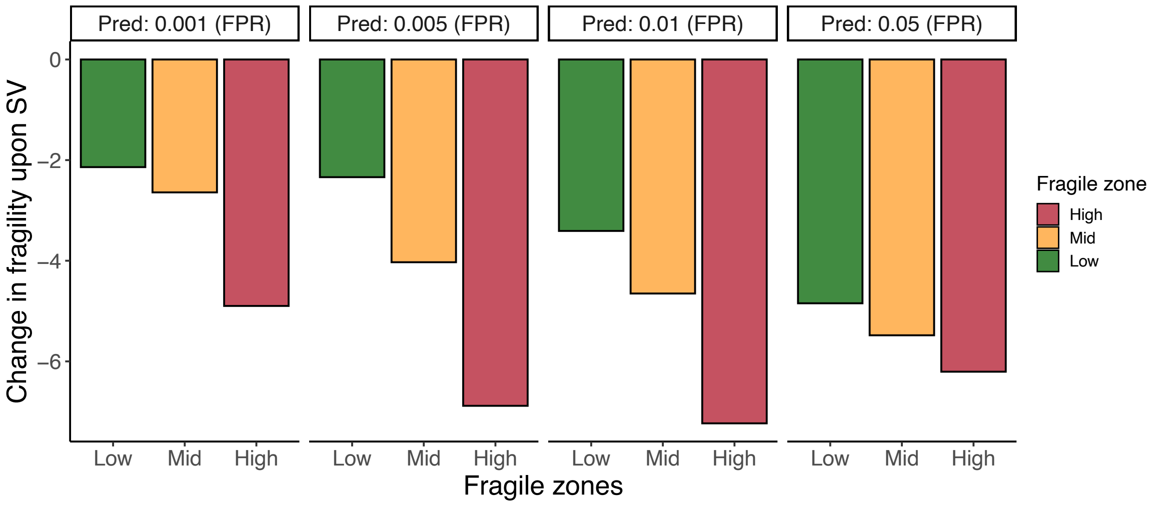

Figure S33. Average change in sequence fragility of structural variants found in low, medium, and high fragile regions. *We binned the entire human genome into non-overlapping 1,960 bases-long regions (see Materials and Methods for further details). Within each of these bins, we counted the total number of predicted DNA strand breaks and classified the bins into three fragility levels: the bottom and top 5% of the distribution of breaks as "low" and "high" fragility, respectively, as an indicator of cold and hot break zones, and the remainder as "medium" fragility. The results show that SVs found in highly fragile regions tend to contribute to their stabilisation upon emergence. Similar patterns are observed in SVs found in medium fragile regions. In contrast, SVs in low fragile regions of the human genome tend to destabilise the region by increasing the regional fragility, potentially increasing susceptibility to further SV occurrences.*

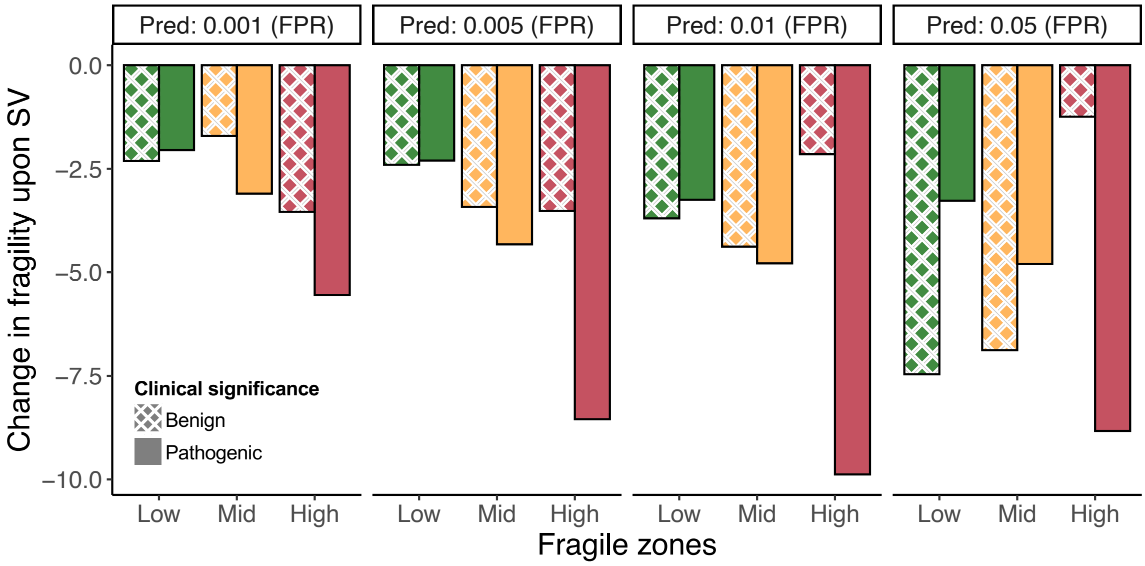

Figure S34. Average change in sequence fragility of structural variants found in low, medium, and high fragile regions. *We binned the entire human genome into non-overlapping 1,960 bases-long regions (see Materials and Methods for further details). Within each of these bins, we counted the total number of predicted DNA strand breaks and classified the bins into three fragility levels: the bottom and top 5% of the distribution of breaks as "low" and "high" fragility, respectively, as an indicator of cold and hot break zones, and the remainder as "medium" fragility. The results show that SVs found in highly fragile regions tend to contribute to their stabilisation upon emergence. Similar patterns are observed in SVs found in medium fragile regions. In contrast, SVs in low fragile regions of the human genome tend to destabilise the region by increasing the regional fragility, potentially increasing susceptibility to further SV occurrences. Each SV is further divided into its benign or pathogenic clinical label. We find that the overall trend is similar across both clinical SV types but strongly contrasts in SVs found within low fragile regions. Interestingly, pathogenic SVs found in highly fragile zones decrease the regional fragility the most, potentially lowering the susceptibility to further SV occurrences.*

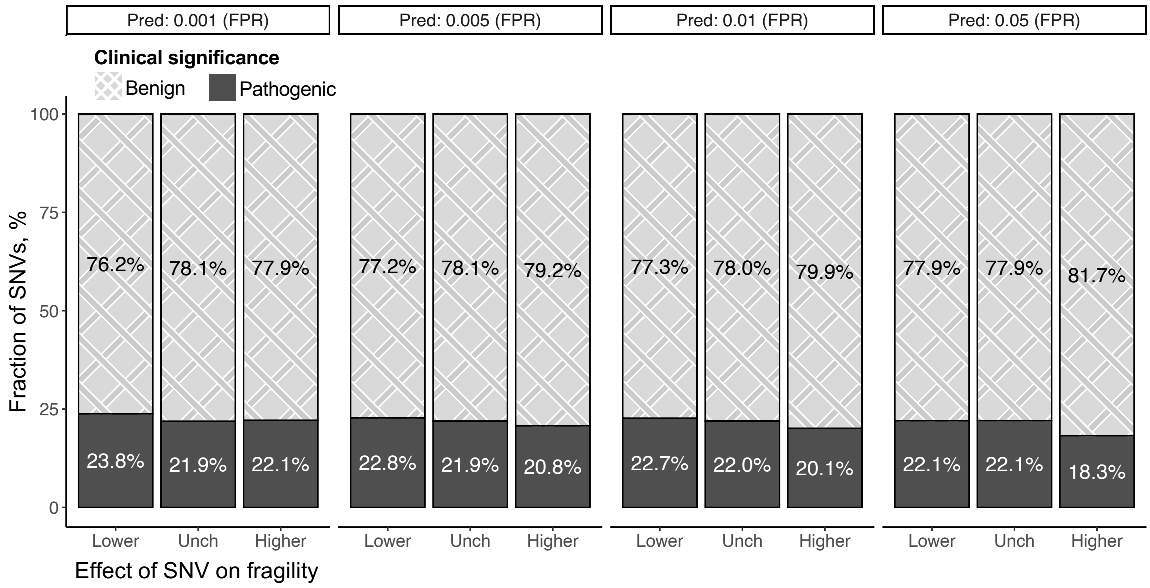

Figure S35. Predicted DNA strand breaks at clinically significant single nucleotide variant (SNV) sites. *For each SNV, we predicted its fragility. Then, by comparing the sequence pre- and post-SNV, we compared whether the SNV led to a break or not. Separately for benign and pathogenic variants, we plot for each effect on the x-axis its fractional presence on the y-axis. This was done independently for each of the four different thresholds applied to our model's predictions.*

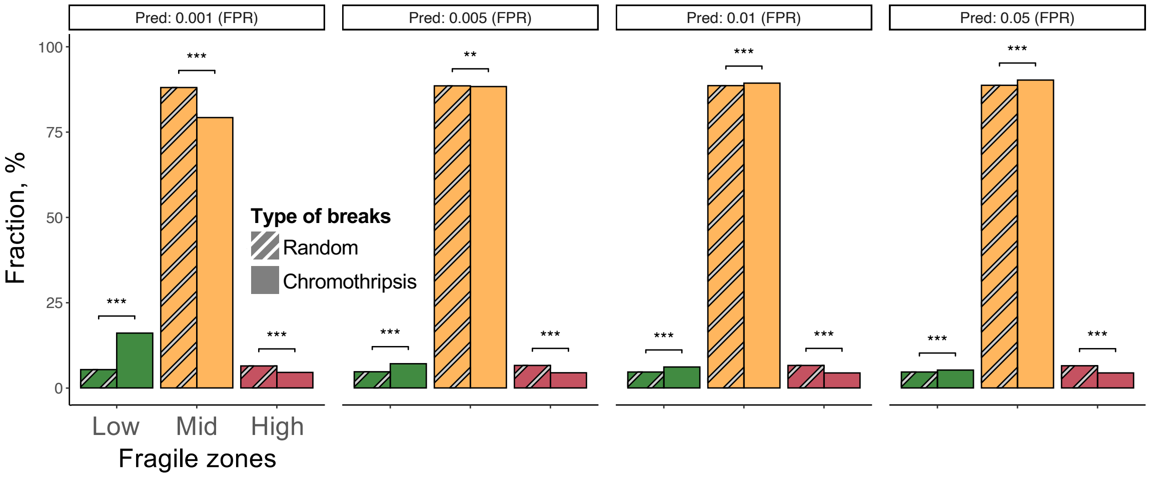

Figure S36. Predicted DNA fragility classified into low, medium, and high fragility obtained from 20 kb-long non-overlapping bins coinciding with chromothripsis breakpoint junctions. *We binned the genome into non-overlapping regions of 20 kb-long intervals and counted the number of strand breaks predicted to be in each bin, separately for each of the four thresholds applied to our model's predictions. From this distribution, we extracted the bins corresponding to the bottom and top 5% of the density of strand breaks. We labelled them as "low" and "high" fragility, respectively, while the remaining were labelled as "medium" fragility, which merely corresponds to the remainder of the distribution. Taking all processed chromothripsis breakpoint positions, we calculated their fractional overlap within the three break zones. We also randomly sampled 1 million genomic positions across all autosomes and repeated the same calculations. We performed a z-test for a difference in the proportions between the chromothripsis breakpoints and randomly sampled negative control breakpoints, separately for each of the four model's thresholds. The statistical significance is annotated on top of the bars (**** $p < 0.001$, **** $p < 0.01$).*

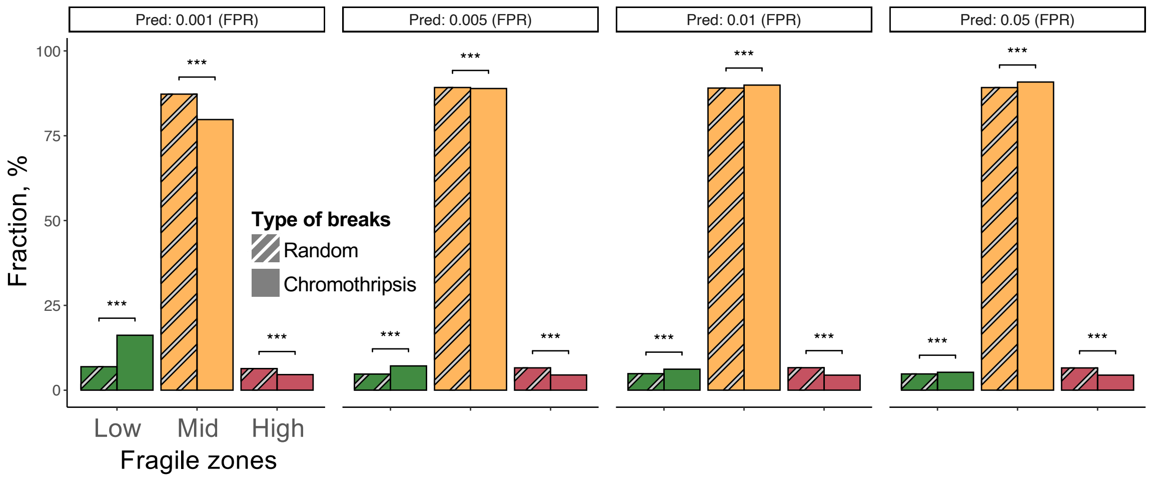

Figure S37. Predicted DNA fragility classified into low, medium, and high fragility obtained from 10 kb-long non-overlapping bins coinciding with chromothripsis breakpoint junctions. *We binned the genome into non-overlapping regions of 10 kb-long intervals and counted the number of strand breaks predicted to be in each bin, separately for each of the four thresholds applied to our model's predictions. From this distribution, we extracted the bins corresponding to the bottom and top 5% of the density of strand breaks. We labelled them as "low" and "high" fragility, respectively, while the remaining were labelled as "medium" fragility, which merely corresponds to the remainder of the distribution. Taking all processed chromothripsis breakpoint positions, we calculated their fractional overlap within the three break zones. We also randomly sampled 1 million genomic positions across all autosomes and repeated the same calculations. We performed a z-test for a difference in the proportions between the chromothripsis breakpoints and randomly sampled negative control breakpoints, separately for each of the four model's thresholds. The statistical significance is annotated on top of the bars (******* $p < 0.001$).*

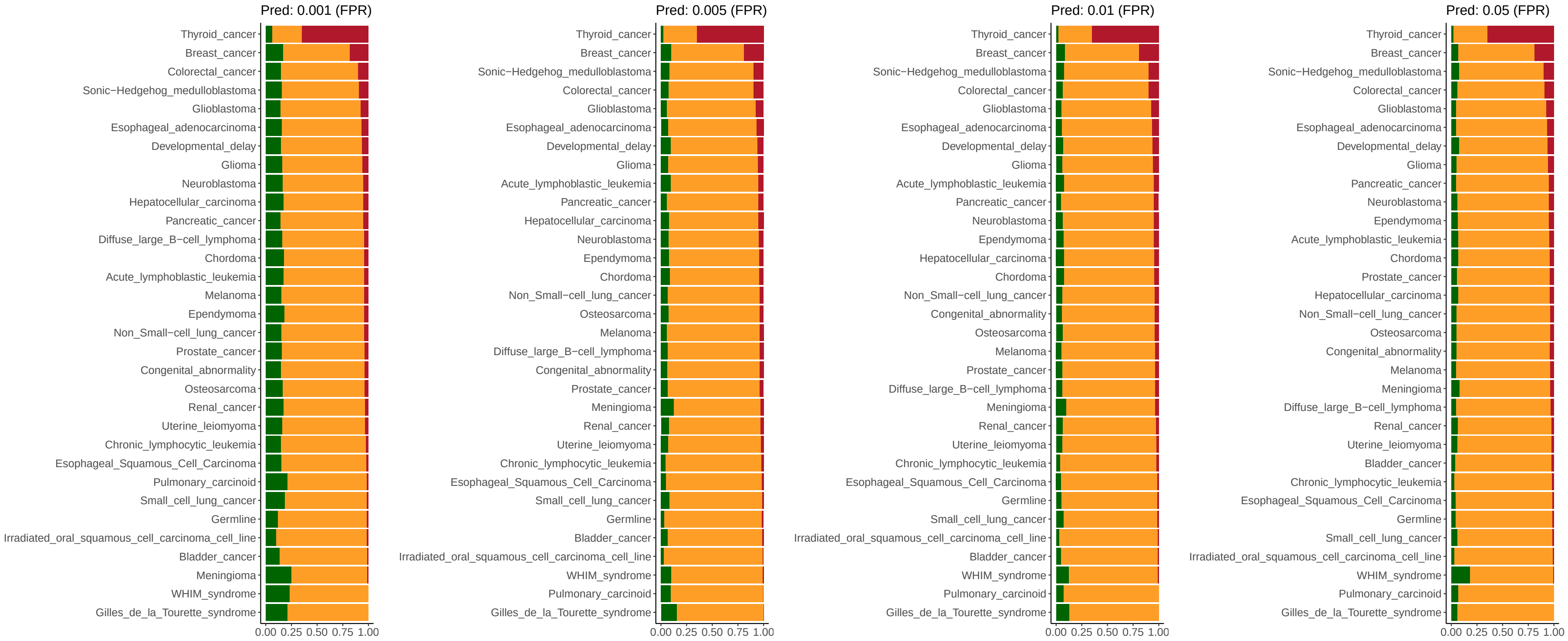

Figure S38. Predicted DNA fragility classified into low, medium, and high fragility obtained from 20 kb non-overlapping bins coinciding with chromothripsis breakpoint junctions. *The x-axis represents the relative fragility, calculated as the total number of predicted DNA strand breaks coinciding with the position of the chromothripsis breakpoint normalised by the total number of breaks for the type of cancer or disease. The stacked barplot shows the percentage of each of the low (green), medium (orange), and high (red) fragility. This is obtained by binning the genome into non-overlapping regions of 20 kb and counting the number of predicted strand breaks in each bin. From this distribution, we extract the bins corresponding to the bottom and top 5% of the density of strand breaks and label them as "low" and "high" fragility, respectively, while the remaining are labelled as "medium" fragility, which merely corresponds to the remainder of the distribution. The genomic features on the y-axis are ordered from the highest to lowest percentage share of highly fragile sites compared to the remaining low and medium fragile sites. The four plots are associated with DNA strand breaks predicted by the LightGBM model, ordered by the increasing rate of false positives, illustrated at the top of each plot.*

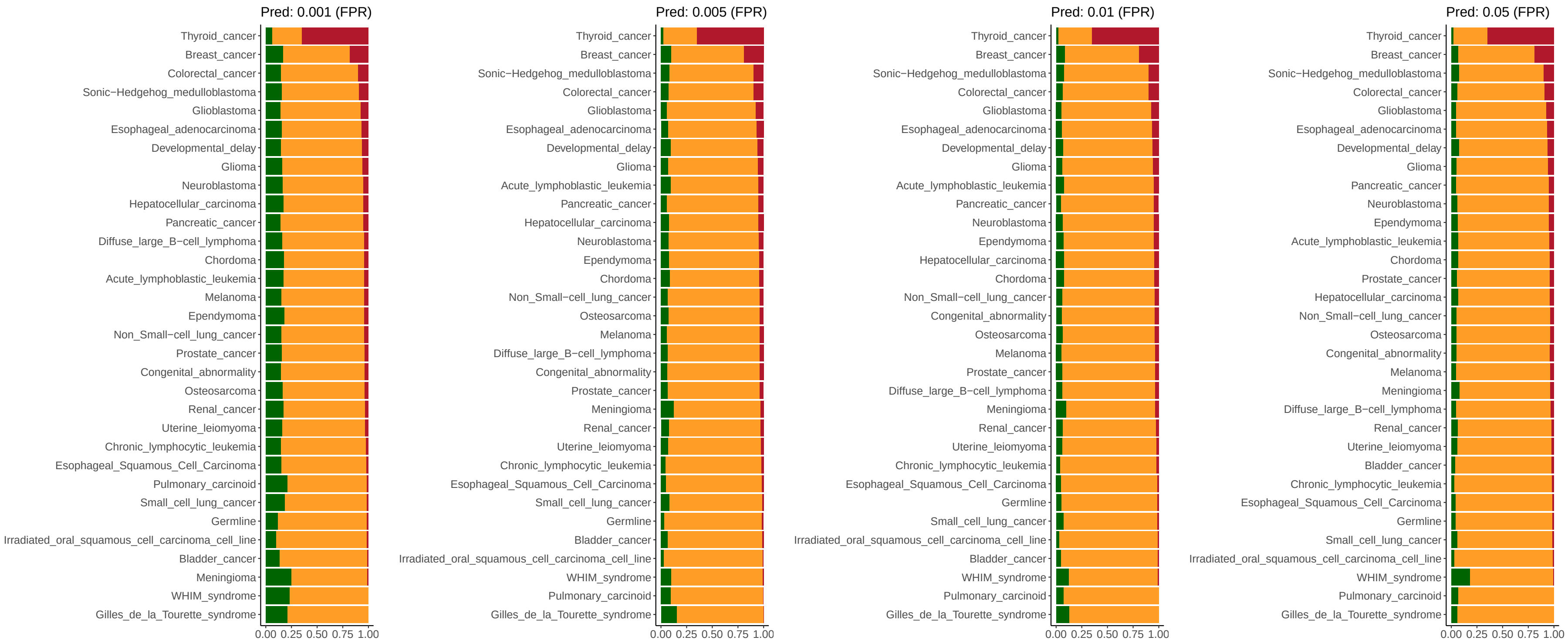

Figure S39. Predicted DNA fragility classified into low, medium, and high fragility obtained from 10 kb non-overlapping bins coinciding with chromothripsis breakpoint junctions. *The x-axis represents the relative fragility, calculated as the total number of predicted DNA strand breaks coinciding with the position of the chromothripsis breakpoint normalised by the total number of breaks for the type of cancer or disease. The stacked barplot shows the percentage of each of the low (green), medium (orange), and high (red) fragility. This is obtained by binning the genome into non-overlapping regions of 10 kb and counting the number of predicted strand breaks in each bin. From this distribution, we extract the bins corresponding to the bottom and top 5% of the density of strand breaks and label them as "low" and "high" fragility, respectively, while the remaining are labelled as "medium" fragility, which merely corresponds to the remainder of the distribution. The genomic features on the y-axis are ordered from the highest to lowest percentage share of highly fragile sites compared to the remaining low and medium fragile sites. The four plots are associated with DNA strand breaks predicted by the LightGBM model, ordered by the increasing rate of false positives, illustrated at the top of each plot.*

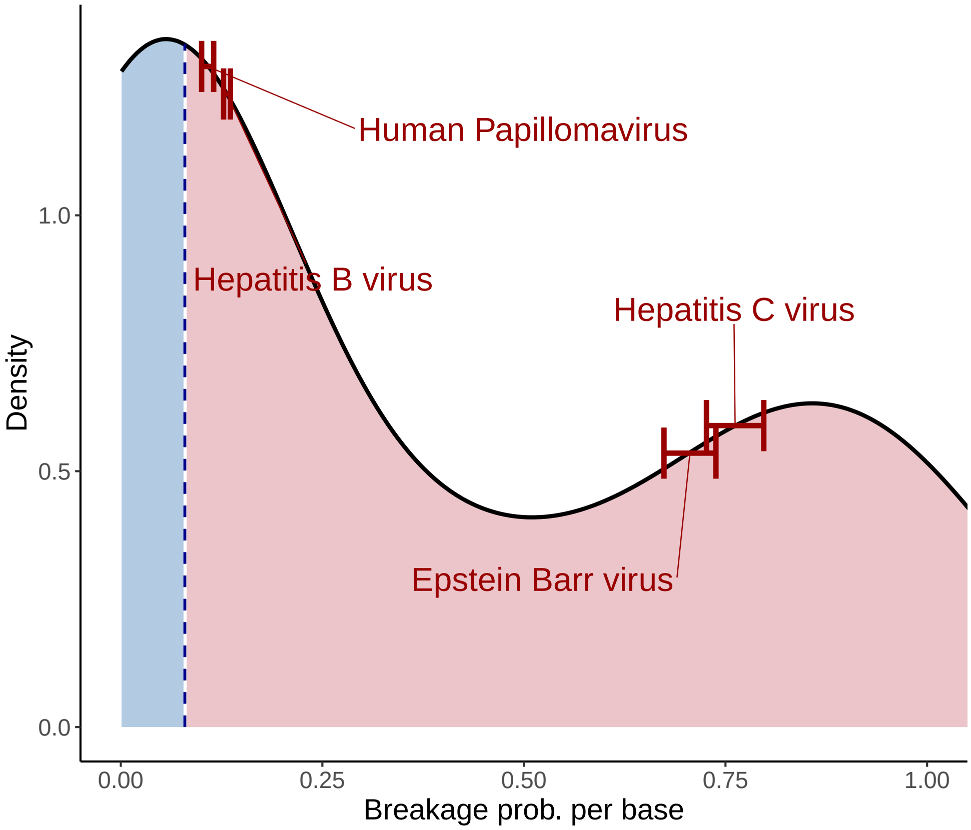

Figure S40. Sequence fragility of DNA virus sequences integrated into a host genome. *We used our model to scan each viral sequence at every basal position to assess its overall fragility. For each virus, we normalised the predicted number of strand breaks by its sequence length to obtain the probability per base metric. The results are presented as a density plot, highlighting the four cancer-associated DNA viruses in red and the average human genome fragility in blue. If multiple strains of the same cancer-associated viral species appear in the dataset, we annotate the mean and standard error range of its sequence fragility.*

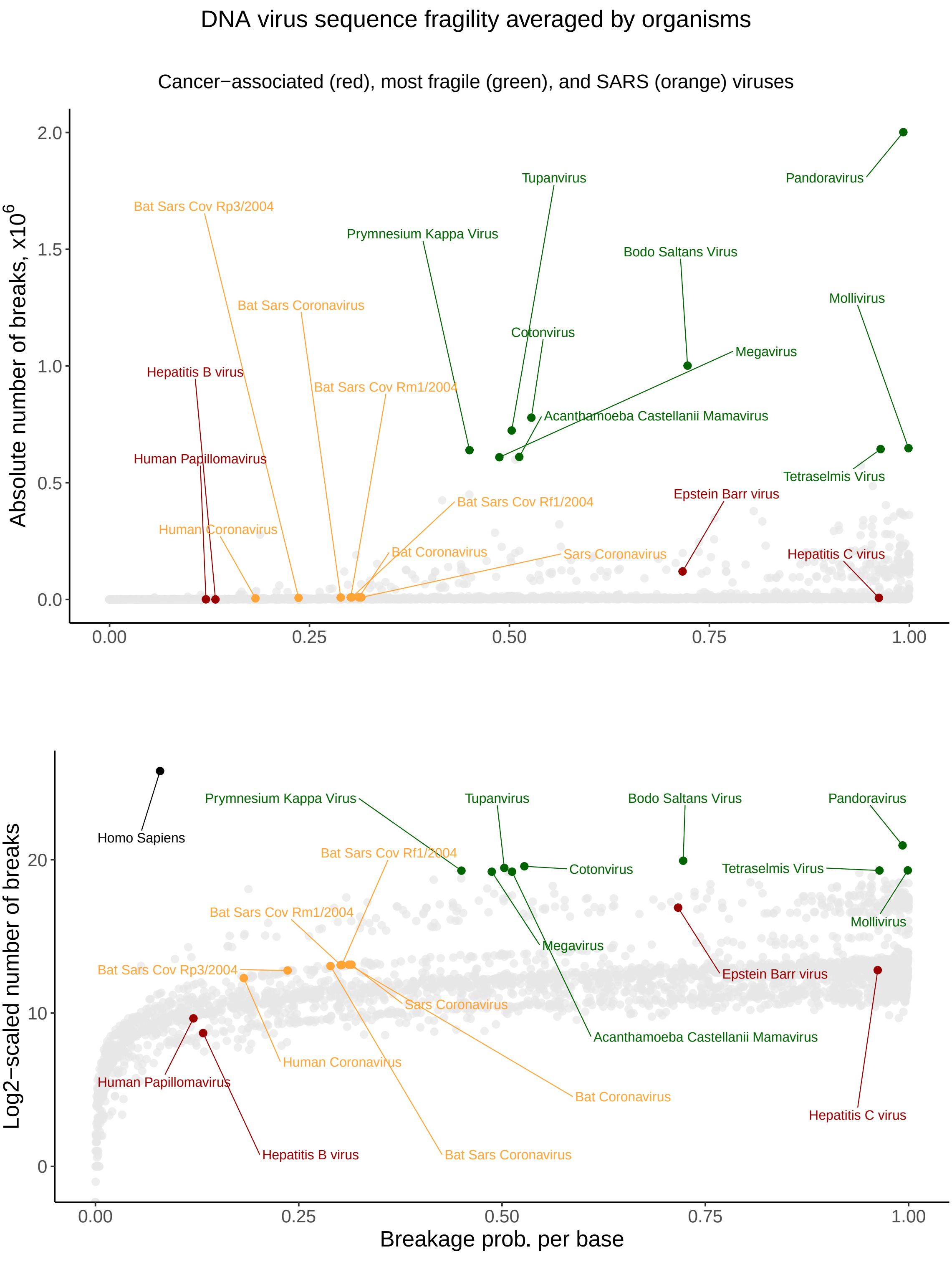

***Figure S41. Absolute and normalised sequence fragility of various DNA virus species**. We used our model to scan each viral sequence at every basal position to assess its overall fragility. For each virus, we normalised the predicted number of strand breaks by its sequence length to obtain the probability per base metric on the x-axis. On the y-axis, we present the absolute number of breaks (**top**) or the $\log_2$-scaled number of breaks (**bottom**). The results are presented as a scatter plot, highlighting the four cancer-associated DNA viruses in red, severe acute respiratory syndrome (SARS) viruses in orange, and some of the most fragile viral species taking into account both their absolute and normalised fragility.*

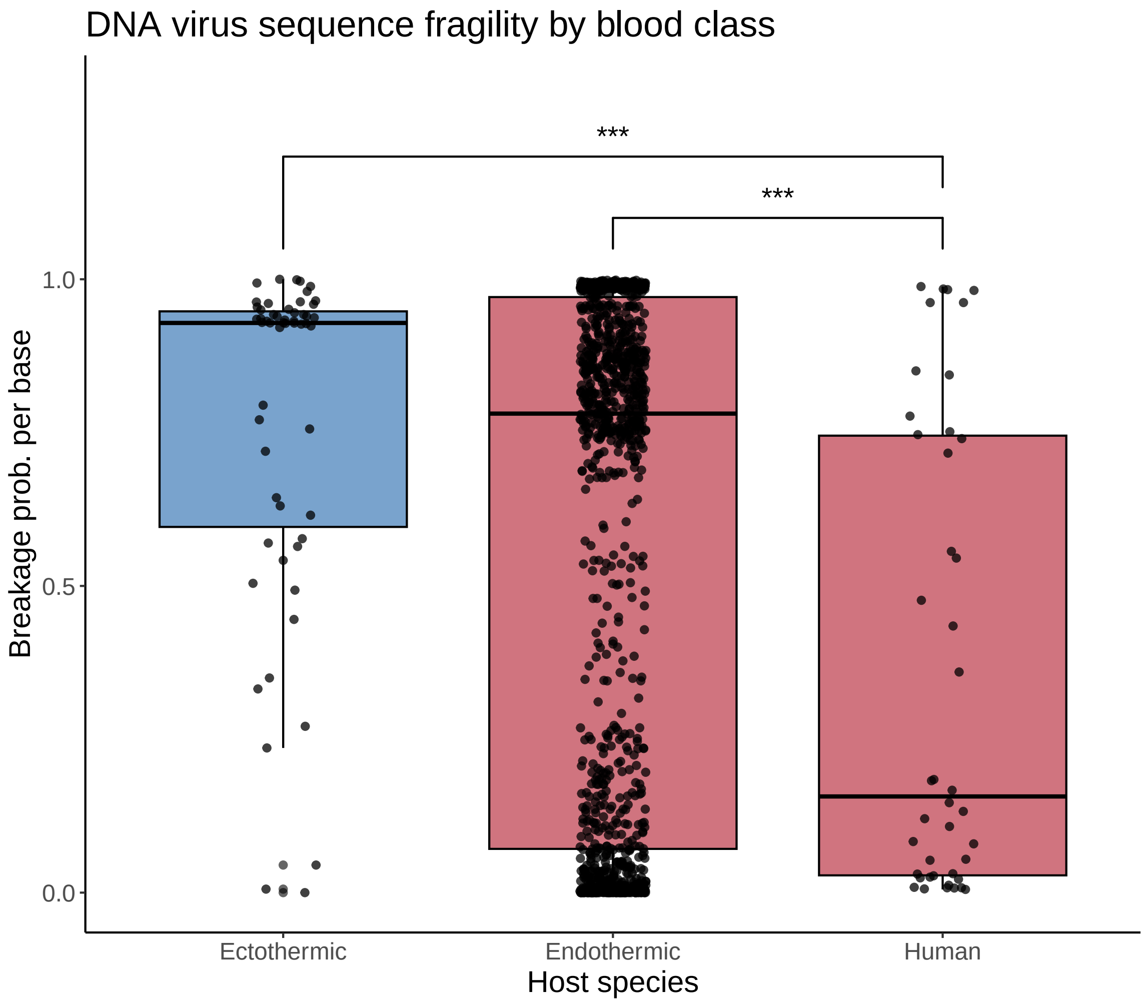

Figure S42. Sequence fragility of DNA viruses infecting the human, ectothermic, and endothermic animals. *We used our model to scan each viral sequence at every basal position to assess its overall fragility. For each virus, we normalised the predicted number of strand breaks by its sequence length to obtain the probability per base metric on the y-axis. Each DNA viral species is presented in a boxplot corresponding to its label as a virus infecting the human, ectothermic or endothermic animals. A two-sample t-test was performed between the virus fragilities in humans (red) versus ectothermic (blue) and endothermic (red) animals. The statistical significance is annotated over the boxplot (**** $p < 0.001$*.)*

Primary Tissue	Primary Cancer	Count
Breast	Carcinoma	338,707
Biliary tract	Carcinoma	323,098
Prostate	Carcinoma	270,816
Haematopoietic and lymphoid-tissue	Lymphoid neoplasm	187,247
Ovary	Carcinoma	124,854
Large intestine	Carcinoma	123,906
Pancreas	Carcinoma	109,151
Stomach	Carcinoma	94,372
Skin	Carcinoma	55,852
Liver	Carcinoma	55,143
Upper aerodigestive tract	Carcinoma	27,961
Central nervous system	Glioma	27,922
Lung	Carcinoma	27,744
Skin malignant	Melanoma	23,383
Haematopoietic and lymphoid tissue	Haematopoietic neoplasm	20,401
Endometrium	Carcinoma	15,659
Soft tissue	Undifferentiated unclassified sarcoma	12,641

Table S1. *Primary tissue and primary cancer pairs that constitute 95% of the COSMIC database-reported cancer-associated DNA strand breaks.*

Threshold	False Positive Rate	True Positive Rate	TPR:FPR Ratio
0.945	0.001	0.135	134.947
0.876	0.005	0.246	49.165
0.826	0.010	0.316	31.586
0.621	0.050	0.556	11.116

Table S2. *The four different prediction thresholds of the model are based on the preferred target false positive rate. All rate and probability values lie between 0 and 1.*

Bibliography

[1] Henrike Johanna Gothe et al. "Spatial Chromosome Folding and Active Transcription Drive DNA Fragility and Formation of Oncogenic MLL Translocations". *Molecular Cell* (July 2019). DOI: 10.1016/j.molcel.2019.05.015.

[2] Matthew W. Snyder et al. "Cell-free DNA Comprises an In Vivo Nucleosome Footprint that Informs Its Tissues-Of-Origin". *Cell* (Jan. 2016). DOI: 10.1016/j.cell.2015.11.050.

[3] Kay Prüfer et al. "The complete genome sequence of a Neanderthal from the Altai Mountains". *Nature* (Jan. 2014). DOI: 10.1038/nature12886.

[4] Stefanie V. Lensing et al. "DSBCapture: in situ capture and sequencing of DNA breaks". *Nature Methods* (Oct. 2016). DOI: 10.1038/nmeth.3960.

[5] Swapan Mallick et al. "The Simons Genome Diversity Project: 300 genomes from 142 diverse populations". *Nature* (Oct. 2016). DOI: 10.1038/nature18964.

[6] Huifen Cao et al. "Novel approach reveals genomic landscapes of single-strand DNA breaks with nucleotide resolution in human cells". *Nature Communications* (Dec. 2019). DOI: 10.1038/s41467-019-13602-7.
